# Supplementary material for: Somatic mutation and selection at population scale
Source: Nature. 2025 Oct 8;647(8089):411–20. doi: 10.1038/s41586-025-09584-w (PMC12611758; doi:10.1038/s41586-025-09584-w)
Supplement: Supplementary file 2 — Reporting Summary [file 41586_2025_9584_MOESM2_ESM.pdf]

nature portfolio

Corresponding author(s): Inigo Martincorena

Last updated by author(s): Oct 30, 2024

Reporting Summary

Nature Portfolio wishes to improve the reproducibility of the work that we publish. This form provides structure for consistency and transparency in reporting. For further information on Nature Portfolio policies, see our Editorial Policies and the Editorial Policy Checklist.

Statistics

For all statistical analyses, confirm that the following items are present in the figure legend, table legend, main text, or Methods section.

n/a	Confirmed	
☐	☒	The exact sample size (*n*) for each experimental group/condition, given as a discrete number and unit of measurement
☐	☒	A statement on whether measurements were taken from distinct samples or whether the same sample was measured repeatedly
☐	☒	The statistical test(s) used AND whether they are one- or two-sided<br>*Only common tests should be described solely by name; describe more complex techniques in the Methods section.*
☐	☒	A description of all covariates tested
☐	☒	A description of any assumptions or corrections, such as tests of normality and adjustment for multiple comparisons
☐	☒	A full description of the statistical parameters including central tendency (e.g. means) or other basic estimates (e.g. regression coefficient) AND variation (e.g. standard deviation) or associated estimates of uncertainty (e.g. confidence intervals)
☐	☒	For null hypothesis testing, the test statistic (e.g. *F*, *t*, *r*) with confidence intervals, effect sizes, degrees of freedom and *P* value noted<br>*Give P values as exact values whenever suitable.*
☒	☐	For Bayesian analysis, information on the choice of priors and Markov chain Monte Carlo settings
☐	☒	For hierarchical and complex designs, identification of the appropriate level for tests and full reporting of outcomes
☐	☒	Estimates of effect sizes (e.g. Cohen's *d*, Pearson's *r*), indicating how they were calculated

Our web collection on statistics for biologists contains articles on many of the points above.

Software and code

Policy information about availability of computer code

Data collection	No software was used for data collection
Data analysis	Nanorate sequencing data analysis relied on the NanoSeq pipeline, available at: https://github.com/cancerit/NanoSeq, version 3.3.0. Targeted methylation analyses were done with R package EpiDish v2.20. Most analyses have been done with bespoke pipelines, provided as an accompanying R markdown in HTML format. Sigfit v_2.1.0 and v_2.0 for signature analysis. Our pipeline makes use of samtools v1.9, bcftools v1.9, bwa v0.7.5a-r405, bedtools v2.29.0, Kraken 2.1.2.

For manuscripts utilizing custom algorithms or software that are central to the research but not yet described in published literature, software must be made available to editors and reviewers. We strongly encourage code deposition in a community repository (e.g. GitHub). See the Nature Portfolio guidelines for submitting code & software for further information.

Data

Policy information about availability of data

All manuscripts must include a data availability statement. This statement should provide the following information, where applicable:

- Accession codes, unique identifiers, or web links for publicly available datasets
- A description of any restrictions on data availability
- For clinical datasets or third party data, please ensure that the statement adheres to our policy

Sequencing data has been deposited in EGA under accession numbers EGAD00001015618 (TwinsUK_TargetedNanoSeq_Buccal), EGAD00001015619 (TwinsUK_TargetedNanoSeq_Blood), EGAD00001015620 (TwinsUK_ExomeNanoSeq_Buccal), EGAD00001015621 (TwinsUK_RENanoSeq_Buccal), EGAD00001015622 (TwinsUK_TargetedEMSeq_Buccal), EGAD00001015623 (TwinsUK_TargetedEMSeq_Blood), and EGAD00001015624 (Sanger_NanoSeq_RandD). Data access for EGAD00001015618, EGAD00001015619, EGAD00001015620, EGAD00001015621, EGAD00001015622 and EGAD00001015623 is managed by TwinsUK (EGAC00001000274) (Supplementary Table 7). Supporting code can be found in https://github.com/cancerit/NanoSeq/, https://github.com/im3sanger/dndscv, and as an accompanying R HTML MarkDown file. Patient metadata is managed by TwinsUK. Anonymised mutational data is available in Supplementary Tables 8 and 9. All analyses have been done with human genome GRCh37.

Research involving human participants, their data, or biological material

Policy information about studies with human participants or human data. See also policy information about sex, gender (identity/presentation), and sexual orientation and race, ethnicity and racism.

Reporting on sex and gender	We only focused on sex, not gender, for regression analyses where we used it as a covariate. For several analyses of twin pairs, such as the heritability analyses, we only used same sex twins.
Reporting on race, ethnicity, or other socially relevant groupings	We don't make any report on ethnicities or other socially relevant groupings
Population characteristics	Our cohort includes 1,047 donors, mean age of 65.2 years, mostly healthy with some donors reporting previous history of cancer, as well as oral health, body mass index, and medication.
Recruitment	From a preselection of 4,800 donors, we invited 1,796 to participate based on several criteria, receiving buccal swabs from 1,236. From the final 1,042 donors in the study, we also selected 380 individuals with available material the the TwinsUK BioBank for sequencing of archival whole-blood DNA.<br>To increase our statistical power to study associations with exposures, risk factors and germline factors, we included all available donors of age 80 or higher (n=230), as many complete twin pairs as possible, smokers, individuals with obesity (BMI > 30), and individuals with available genome-wide genotyping information. We also favoured the selection of males and individuals of non-white ethnicity to reduce some of the demographic biases in the TwinsUK registry compared to the general population. To test for associations between the mutational landscape and medications or clinical histories, we favoured the inclusion of individuals with a history of cancer (including all donors with a history of oral cancer, n=12) or a self-reported treatment history including tamoxifen, immunosuppressants, metformin, aspirin or ibuprofen. 194 samples were excluded from analysis based on several sequencing quality metrics, leaving a total of 1,042 samples in the study. Exclusion criteria included: removal of contaminated samples with either human (n=17) or non-human (n=132) DNA, exclusion of samples with mean duplex coverage lower than 50 (n=79), and exclusion of swabs with genotyping information not matching the pre-existing genotyping information from TwinsUK (n=7)
Ethics oversight	The use of these samples was approved initially by the North West Research Ethics Committee (REC 19/NW/0187) and later renewed by the same committee (REC 24/NW/0106), and informed consent was provided by participants.

Note that full information on the approval of the study protocol must also be provided in the manuscript.

Field-specific reporting

Please select the one below that is the best fit for your research. If you are not sure, read the appropriate sections before making your selection.

[x] Life sciences [ ] Behavioural & social sciences [ ] Ecological, evolutionary & environmental sciences

For a reference copy of the document with all sections, see nature.com/documents/nr-reporting-summary-flat.pdf

Life sciences study design

All studies must disclose on these points even when the disclosure is negative.

Sample size	Information on sample sizes is provided for all analyses. We estimated that, with optical duplicate rates, sequencing coverage translates into effective coverage on a 1/20 ratio. The requested sequencing coverage was chosen based on the amount of DNA for each sample. We aimed at a cohort size of 1000 donors, ending up with a cohort of 1042. This cohort size was chosen based on budgetary considerations.

Data exclusions	194 samples were excluded from analysis based on several sequencing quality metrics, leaving a total of 1,042 samples in the study. Exclusion criteria included: removal of contaminated samples with either human (n=17) or non-human (n=132) DNA, exclusion of samples with mean duplex coverage lower than 50 (n=79), and exclusion of swabs with genotyping information not matching the pre-existing genotyping information from TwinsUK (n=7).
Replication	We haven't conducted explicit replication as this was not applicable for an observational study
Randomization	No randomization was performed as this was not applicable for an observational study
Blinding	No blinding was undertaken because this is an observational study.

Reporting for specific materials, systems and methods

We require information from authors about some types of materials, experimental systems and methods used in many studies. Here, indicate whether each material, system or method listed is relevant to your study. If you are not sure if a list item applies to your research, read the appropriate section before selecting a response.

Materials & experimental systems

n/a	Involved in the study
☒	☐ Antibodies
☒	☐ Eukaryotic cell lines
☒	☐ Palaeontology and archaeology
☒	☐ Animals and other organisms
☒	☐ Clinical data
☒	☐ Dual use research of concern
☒	☐ Plants

Methods

n/a	Involved in the study
☒	☐ ChIP-seq
☒	☐ Flow cytometry
☒	☐ MRI-based neuroimaging

Plants

Seed stocks	*Report on the source of all seed stocks or other plant material used. If applicable, state the seed stock centre and catalogue number. If plant specimens were collected from the field, describe the collection location, date and sampling procedures.*
Novel plant genotypes	*Describe the methods by which all novel plant genotypes were produced. This includes those generated by transgenic approaches, gene editing, chemical/radiation-based mutagenesis and hybridization. For transgenic lines, describe the transformation method, the number of independent lines analyzed and the generation upon which experiments were performed. For gene-edited lines, describe the editor used, the endogenous sequence targeted for editing, the targeting guide RNA sequence (if applicable) and how the editor was applied.*
Authentication	*Describe any authentication procedures for each seed stock used or novel genotype generated. Describe any experiments used to assess the effect of a mutation and, where applicable, how potential secondary effects (e.g. second site T-DNA insertions, mosiacism, off-target gene editing) were examined.*
