# Supplementary material for: Somatic mutation and selection at population scale
Source: Nature. 2025 Oct 8;647(8089):411–20. doi: 10.1038/s41586-025-09584-w (PMC12611758; doi:10.1038/s41586-025-09584-w)
Supplement: Supplementary file 12 — This zipped file contains the Supplementary Code. [file 41586_2025_9584_MOESM12_ESM.zip › Supplementary_code_v23.html]

Somatic mutation and selection at epidemiological scale


Code 

- Show All Code
- Hide All Code

# Somatic mutation and selection at epidemiological scale

#### 24 March, 2025

## 1. Driver discovery: positive selection analyses with dNdScv

### Input files and environment

List of input files used in this script.

```
# Input files
mutations_file = "2023-12-21_TwinsUK_CompleteTNanoSeqCalls_ExcludeSamplesQposArtefact_IndelsFiltered.v3.tsv"
dcpergene_file = "2023-11-10_TwinsUK_CompleteMeanCov_ExcludeSamplesQposArtefact.tsv"
genelist_file = "Sanger_TERT-v4_TE-95148282_hg19_highstringencyfilter_buccal_gene_list.tsv"
metadata_file = "metadata.twinsuk.20241017.tsv"
extended_medications_file = "extended_medications_and_metadata_for_regressions.csv"
extended_cancer_file = "TwinsUK_metadata_drugs_cancer_combined.csv"
diabetes_file = "medications_for_regressions_diabetes.csv"
genome_file = "/Users/im3/Desktop/GRCh37_human_genome.fa"
burdens_file = "burdens.2023-11-21.tsv"
burdensWGS_file = "2024-06-28_RENanoSeq_CombinedBurden.tsv"
essentialgenes_file = "CRISPRInferredCommonEssentials.csv" # Alternatively: "Essential_genes_CEGv2.txt"
tcga_HNSC_calls_file = "TCGA_MC3_HNSC.5col"
excluded_samples_file = "SAMPLES2EXCLUDE.2023-11-15.tsv"
other_excluded_samples_from_regressions = ""
genome_length = 2835673629*2 # Length of a diploid XX genome ungapped length placed in scaffold with chrY subtracted.
#genome_length = 3054815472*2 # Length of a diploid XX genome based on PMID:35357919
dndscv_refdb = "RefCDS_GRCh37_Nanoseq_blacklisted.Rdat"
dndscv_covs = "covariates_20pc_GRCh37-38.epi_strict_outliers.Rdat"
# Blood data
bloodmutations_file = "2024-01-04_TwinsUK_BloodMatchedBuccal_CompleteTNanoSeqCalls_Exc9MismatchedBuccalDonors_ExcludeQposArtefactFilteredIndels.tsv"
bloodburdens_file = "burdens_and_meta_blood.380nonunique.autosomes.IncludingBootstrapping.tsv"
dcpergene_blood_file = "2024-01-04_TwinsUK_BloodMatchedBuccal_CompleteMeanCov_Exc9MismatchedBuccalDonors.tsv"
blooddonorcriteria_file = "2024-02-01_Blood_donor_selection_criteria.tsv"
bloodclones_inbuccal_file = "VAFS_BLOOD_CLONES_IN_BUCCALS.tsv"
# Exome nanoseq buccal data
exomemuts_file = "plate_042_SQPP-22755-I_exome_final_muts.tsv"
exomedc_file = "plate_042_SQPP-22755-I_exome_coverage_info.tsv"
# Signatures and mutational spectra files
signatures_denovo_file = "2023-12-07_TwinsUK_Exposures_DeNovo_2.tsv"
signatures_cosmic_file = "2023-12-07_TwinsUK_Exposures_COSMIC_3.tsv"
signatures_spectra_file = "TwinsUK_Sigs_SBS96_Normalised.tsv"
trinuc_frequencies_sbs96_WG_file = "Genome_Frequencies_SBS96_hg19.tsv" # Whole-genome trinuc counts
trinuc_frequencies_sbs96_TG_file = "TwinsUK_Frequencies_SBS96_Buccals.tsv" # Targeted trinuc counts
# Cosmic mutation files
cosmic_mutation_file = "COSMIC_v99_WholeGenomeExome_dNdSAnnotatedMuts.tsv"
cosmic_samples_file = "COSMIC_v99_WholeGenomeExome_SamplePhenotypeInfo.tsv"
clinvar_files = c(NOTCH1="NOTCH1_withingene_sites_badSplice.tsv", TP53="TP53_withingene_sites.tsv", PPM1D="PPM1D_withingene_sites_badSplice.tsv")
risk_alleles_file = "Cancer_risk_SNPs_combined_genotypes.tsv"
use_indel_sites = F # Choice to use unique indel sites or total number of indels in dNdScv (default is "T" but for TwinsUK we recommend "F")

# Copying all input files into "Input_files" folder
if (0) {
  system("mkdir Input_files")
  files = c(mutations_file, dcpergene_file, genelist_file, metadata_file, extended_medications_file, extended_cancer_file, diabetes_file, burdens_file, burdensWGS_file, essentialgenes_file, tcga_cancer_calls_file, excluded_samples_file, dndscv_refdb, dndscv_covs, bloodmutations_file, bloodburdens_file, dcpergene_blood_file, blooddonorcriteria_file, bloodclones_inbuccal_file, exomemuts_file, exomedc_file, signatures_denovo_file, signatures_cosmic_file, signatures_spectra_file, trinuc_frequencies_sbs96_WG_file, trinuc_frequencies_sbs96_TG_file, cosmic_mutation_file, cosmic_samples_file, as.vector(clinvar_files))
  for (j in 1:length(files)) { system(sprintf("cp %s Input_files/%s", files[j], files[j])) }
}

set.seed(123)
runman = F # Use T when running the code manually to save figures as pdf
```

Loading the data.

```
# Loading the required packages
library(dndscv)
library(Rsamtools)
library(ggvenn)
library(lme4)
library(gplots)
library(RColorBrewer)

# Loading the data
mutations = read.table(mutations_file, header=1, sep="\t", stringsAsFactors=F)
gene2dc = rowSums(read.table(dcpergene_file, header=1, sep="\t", stringsAsFactors=F))
targetgenes = read.table(genelist_file, header=1, sep="\t", stringsAsFactors=F)
targetgenes = targetgenes$gene[targetgenes$target_type!="Hotspot"]
mutations$context = as.vector(scanFa(genome_file, GRanges(mutations$chr, IRanges(mutations$pos-1, mutations$pos+1))))
burdens = read.table(burdens_file, header=1, sep="\t", stringsAsFactors=F)
burdensWGS = read.table(burdensWGS_file, header=1, sep="\t", stringsAsFactors=F)

# Metadata: burden estimates per sample
metadata = read.table(metadata_file, header=1, sep="\t", stringsAsFactors=F)
metadata$duplex_cov = setNames(burdens$mean_duplex_cov,burdens$sample)[metadata$pd]
metadata$burden_subs_passengers = setNames(burdens$burden_subs_passengers,burdens$sample)[metadata$pd]
metadata$burden_indels_passengers = setNames(burdens$burden_indels_passengers,burdens$sample)[metadata$pd]
metadata$dnv_burden = setNames(burdens$dnv_burden,burdens$sample)[metadata$pd]
metadata$noTtoC_burden_passengers = setNames(burdens$noTtoC_burden_passengers,burdens$sample)[metadata$pd]
metadata$TtoC_burden_passengers = setNames(burdens$TtoC_burden_passengers,burdens$sample)[metadata$pd]
metadata$burden_subs_passengers_chrX = setNames(burdens$burden_subs_passengers_chrX,burdens$sample)[metadata$pd]
metadata$pct_selected_bases = setNames(burdens$pct_selected_bases,burdens$sample)[metadata$pd]
metadata$inpanel_f_eff = setNames(burdens$inpanel_f_eff,burdens$sample)[metadata$pd]

# Metadata: signature attributions
s1 = read.table(signatures_denovo_file, header=1, sep="\t", stringsAsFactors = F)
metadata$sig_denovo_sigA = metadata$burden_subs_passengers * setNames(s1[,1],rownames(s1))[metadata$pd]
metadata$sig_denovo_sigB = metadata$burden_subs_passengers * setNames(s1[,2],rownames(s1))[metadata$pd]

s2 = read.table(signatures_cosmic_file, header=1, sep="\t", stringsAsFactors = F)
metadata$sig_cosmic_SBS1 = metadata$burden_subs_passengers * setNames(s2[,1],rownames(s2))[metadata$pd]
metadata$sig_cosmic_SBS5 = metadata$burden_subs_passengers * setNames(s2[,2],rownames(s2))[metadata$pd]
metadata$sig_cosmic_SBS16 = metadata$burden_subs_passengers * setNames(s2[,3],rownames(s2))[metadata$pd]

# Metadata: excluding blacklisted samples
exclsamples = rownames(read.table(excluded_samples_file, header=1, sep="\t", stringsAsFactors=F)) # Vector of excluded samples
mutations = mutations[!(mutations$sampleID %in% exclsamples), ] # Not necessary as the mutation calls are already filtered out
metadata = metadata[!(metadata$pd %in% exclsamples), ]

# Diabetes
t2d = read.table(diabetes_file, header=1, sep=",", stringsAsFactors = F)
metadata$T2DM = setNames(t2d$TUK_T2DM,t2d$PublicID)[metadata$donor]
metadata$DiabetesStatus = setNames(t2d$DiabetesStatus,t2d$PublicID)[metadata$donor]

# Mutation cell fractions assuming heterozygous mutations and correcting for copy number in sex chromosomes for males
males = metadata$pd[metadata$SEX=="M"]
mutations$cellfraction = mutations$duplex_vaf * 2
mutations$cellfraction[mutations$chr %in% c("X","Y") & mutations$sampleID %in% males] = mutations$duplex_vaf[mutations$chr %in% c("X","Y") & mutations$sampleID %in% males]

# Mutation unbiased VAFs bam-cell-fraction
mutations$bam_mut_adj = pmax(0,mutations$bam_mut-mutations$times_called)
mutations$bam_cov_adj = mutations$bam_cov-mutations$duplex_cov
mutations$bam_vaf_adj = mutations$bam_mut_adj / mutations$bam_cov_adj
mutations$bam_adj_cellfraction = mutations$bam_vaf_adj * 2
mutations$bam_adj_cellfraction[mutations$chr %in% c("X","Y") & mutations$sampleID %in% males] = mutations$bam_vaf_adj[mutations$chr %in% c("X","Y") & mutations$sampleID %in% males]

# Cancer
extcancer = read.table(extended_cancer_file, header=1, sep=",", stringsAsFactors = F)
metadata$cancer = setNames(extcancer$cancer_combined, extcancer$PublicID)[metadata$donor]

# Formatting other covariables
metadata$obese = metadata$BMI>=30
metadata$underweight = metadata$BMI<=18.5
metadata$cold_sores_ever_diagnosed_any = !is.na(metadata$cold_sores_ever_diagnosed_first)
metadata$ethnic_white = (metadata$ETHNICITY=="White")
metadata$missingteeth = 3-metadata$num_teeth # New (ordinal) variable representing the increase in missing teeth

# Adding information on additional medications
extended_medications = read.table(extended_medications_file, header=1, sep=",", stringsAsFactors=F)
meds2test = colSums(as.matrix(extended_medications[,-1]))
metadata = metadata[,c("donor",setdiff(colnames(metadata),colnames(extended_medications)))] # We remove previous versions of the columns that we want to add (not necessary if running the code from the start)
metadata = merge(metadata, unique(extended_medications[,c("donor",setdiff(colnames(extended_medications),colnames(metadata)))]), by="donor", all.x=TRUE)

# Extrapolating average alcohol consumption per donor to whole adult life
metadata$drink_years = metadata$avg_total_drink_units / 7 * (metadata$AGE-18) # Drink years (used in PMID:19745021)

# Calculating estimated cigarettes per week during the period of smoking
metadata$collated_cigs_per_week = metadata$cigs.week
metadata$collated_cigs_per_week[which(is.na(metadata$cigs.week))] = 7*metadata$cigs.day[which(is.na(metadata$cigs.week))]
metadata$collated_cigs_per_week[which(metadata$pack_years==0 & is.na(metadata$collated_cigs_per_week))] = 0

# Blood variables: burden and blood sample age
bb = read.table(bloodburdens_file, header=1, sep="\t", stringsAsFactors = F)
bb$burden_subs_passengers[(bb$burden_boot_repl_uci/bb$burden_boot_repl_lci)>5] = NA # excluding blood burdens that are too unreliable due to clonality (as shown by the Poisson bootstrapping confidence intervals)
metadata$burden_subs_passengers_blood = setNames(bb$burden_subs_passengers,substr(bb$sample,1,7))[substr(metadata$pd,1,7)]
bloodages = setNames(bb$AGE, substr(bb$sample,1,7)) # Obtaining the ages of the blood samples (archival samples typically collected a few years before the buccal swabs)
metadata$AGE_blood = bloodages[substr(metadata$pd,1,7)]

# Blood mutations and unbiased bam VAFs
bloodmuts = read.table(bloodmutations_file, header=1, sep="\t", stringsAsFactors=F)
bloodmuts$bam_mut_adj = pmax(0,bloodmuts$bam_mut-bloodmuts$times_called)
bloodmuts$bam_cov_adj = bloodmuts$bam_cov-bloodmuts$duplex_cov
bloodmuts$bam_vaf_adj = bloodmuts$bam_mut_adj / bloodmuts$bam_cov_adj
bloodmuts$bam_adj_cellfraction = bloodmuts$bam_vaf_adj * 2
bloodmuts$bam_adj_cellfraction[bloodmuts$chr %in% c("X","Y") & substr(bloodmuts$sampleID,1,7) %in% substr(males,1,7)] = bloodmuts$bam_vaf_adj[bloodmuts$chr %in% c("X","Y") & substr(bloodmuts$sampleID,1,7) %in% substr(males,1,7)]

# Saving the new metadata file
new_metadata_file = paste(gsub(".tsv","",metadata_file),".output.tsv",sep="")
write.table(metadata, file = new_metadata_file, row.names=F, col.names=T, sep="\t", quote=F)
```

Reporting a few summary metrics about the dataset.

```
message(sprintf("Number of donors: %0.0f", nrow(metadata)))
```

```
## Number of donors: 1042
```

```
message(sprintf("Age: median %0.0f, range %0.0f-%0.0f, IQR %0.0f-%0.0f", median(metadata$AGE), min(metadata$AGE), max(metadata$AGE), quantile(metadata$AGE,0.25), quantile(metadata$AGE,0.75)))
```

```
## Age: median 68, range 21-91, IQR 58-75
```

```
message(sprintf("Smokers: %0.3g%%", 100*mean(metadata$pack_years>0, na.rm=T)))
```

```
## Smokers: 36.7%
```

```
f = table(metadata$twin)
completepair = names(f[f==2])
mzdz = table(metadata$FIXED_ZYGOSITY[metadata$twin %in% completepair])/2
message(sprintf("Twins: %0.0f MZ, %0.0f DZ", mzdz["MZ"], mzdz["DZ"]))
```

```
## Twins: 214 MZ, 118 DZ
```

```
message(sprintf("Buccal: mean duplex coverage %0.2f",mean(metadata$duplex_cov)))
```

```
## Buccal: mean duplex coverage 665.27
```

```
message(sprintf("Blood: mean duplex coverage %0.2f, mean raw coverage %0.2f",mean(bb$mean_duplex_cov), mean(bb$mean_bulk_cov)))
```

```
## Blood: mean duplex coverage 676.41, mean raw coverage 3716.94
```

Plotting the age difference between buccal and blood samples.

```
if (runman) { dev.new(width=4.5, height=5) }
plot(metadata$AGE,metadata$AGE_blood, xlab="Donor age (buccal swab)", ylab="Donor age (archival blood)", xlim=c(18,max(metadata$AGE)), ylim=c(18,max(metadata$AGE)), cex=0.5); abline(a=0, b=1, col="cadetblue")
```

```
if (runman) { dev.copy(pdf,"Age_buccal_vs_blood.pdf",width=4.5,height=5); dev.off() }
```

### Positive selection analyses

Running dNdScv and dNdSloc. Mean duplex depth is used as a correction factor of the offset in the dNdScv model. Genes with evidence of selection on substitutions are excluded from the indel model, for which we run dNdScv twice. By excluding genes under clear selection on substitutions from the background model for indels, the theta (overdispersion) parameter of the indel model increases very significantly, improving the indel model and our ability to detect selection on indels.

By default, dNdScv and dNdSloc perform two-sided tests of selection, where genes can reach significance due to positive or negative selection. This is rarely a problem as most cancer genomics or normal tissue datasets are not powered to detect selection at the level of individual genes. Since the current dataset is powered to detect signals of negative selection in specific genes, we have implemented a new one-sided likelihood ratio tests in dNdScv to separately detect positive and negative selection (new optional argument “onesided=T”).

```
load(dndscv_refdb) # Loads a RefCDS object to be used in dndscv. This object excludes masked sites in Nanoseq from analysis with dNdScv.
load(dndscv_covs); dndscovs = scores # Loads a covariate matrix to be used with the chosen RefCDS.
m = unique(mutations[,1:5]) # Unique mutations per sample (note that the dndscv annotation of the calls could have duplicated some calls)
dndsout = dndscv(m, gene_list = targetgenes, max_muts_per_gene_per_sample = Inf, max_coding_muts_per_sample = Inf, constrain_wnon_wspl = T, mingenecovs = 0, dc = gene2dc/mean(gene2dc), onesided = T, cv = dndscovs, refdb = RefCDS, maxcovs = 10, use_indel_sites = use_indel_sites)
newkc = as.vector(dndsout$sel_loc$gene_name[dndsout$sel_loc$qall_loc<0.01])
dndsout = dndscv(m, gene_list = targetgenes, max_muts_per_gene_per_sample = Inf, max_coding_muts_per_sample = Inf, constrain_wnon_wspl = T, mingenecovs = 0, dc = gene2dc/mean(gene2dc), kc = newkc, outmats = T, onesided = T, cv = dndscovs, refdb = RefCDS, maxcovs = 10, use_indel_sites = use_indel_sites) # Excluding substitution drivers from the indel model

# Writing the dNdScv output table to a file (Extended Data Table)
write.table(dndsout$sel_cv[order(dndsout$sel_cv$psubpos_cv),], file = "EDT3_dNdScv_output.tsv", row.names=F, col.names=T, sep="\t", quote=F)
```

Adding protein-coding annotations to the mutation table.

```
a = dndsout$annotmuts
a$mstr = paste(a$sampleID,a$chr,a$pos,a$mut,sep=":")
mutations$mstr = paste(mutations$sampleID,mutations$chr,mutations$pos,mutations$mut,sep=":")
mutations = merge(mutations, a[,6:ncol(a)], by="mstr", all.x = T)
mutations$mstr = NULL # Removing the mstr field
write.table(mutations, file="TwinsUK_annotated_mutations.tsv", col.names=T, row.names=F, sep="\t", quote=F)
```

We can define drivers using different functions.

```
# Lists of significant genes using the dNdSloc and dNdScv one-sided tests
drivers1 = dndsout$sel_loc[dndsout$sel_loc$qpos_loc<0.01, "gene_name"]
drivers2 = dndsout$sel_cv[dndsout$sel_cv$qglobalpos_cv<0.01, "gene_name"]
drivers3 = dndsout$sel_cv[dndsout$sel_cv$qsubpos_cv<0.01, "gene_name"]
#drivers = base::intersect(base::intersect(drivers1,drivers2),drivers3) 
drivers = drivers3 # As a reference list of core drivers we opt to use qsubpos_cv<0.01 as it is sensitive and more robust to problems with indels (false positives or model violations)
# Venn diagram
if (runman) { dev.new(width=4, height=4) }
d = list(dNdSloc=drivers1, dNdScvglobal=drivers2, dNdScvsubs=drivers3)
ggvenn(d, fill_color = c("#0073C2FF", "#EFC000FF", "#868686FF"), stroke_size = 0.5, set_name_size = 4)
```

```
if (runman) { dev.copy(pdf,"Buccal_drivers_venn_diagram1.pdf",width=4,height=4); dev.off() }
```

We can also calculate one-sided q-values for positive selection on missense mutations using the dndscv output. This can be useful to increase the sensitivity to oncogenes, which tend to be enriched only on missense mutations. The use of global p-values combining the evidence from missense substitutions, truncating (nonsense and splice site) substitutions and indels, can reduce the significance of genes with positive selection specifically acting on missense sites. Those can also be recovered by sitednds or codondnds.

```
# One-sided positive selection for missense mutations
dndsout$sel_cv$pmispos_cv = 1
dndsout$sel_cv$pmispos_cv[dndsout$sel_cv$wmis_cv>1] = dndsout$sel_cv$pmis_cv[dndsout$sel_cv$wmis_cv>1]
dndsout$sel_cv$qmispos_cv = p.adjust(dndsout$sel_cv$pmispos_cv, method="BH")
drivers4 = dndsout$sel_cv[dndsout$sel_cv$qmispos_cv<0.01, "gene_name"]
# Venn diagram
if (runman) { dev.new(width=4, height=4) }
d = list(dNdSloc=drivers1, dNdScvglobal=drivers2, dNdScvsubs=drivers3, dNdScvmis=drivers4)
ggvenn(d, fill_color = c("#0073C2FF", "#EFC000FF", "#868686FF", "#CD534CFF"), stroke_size = 0.5, set_name_size = 4)
```

```
if (runman) { dev.copy(pdf,"Buccal_drivers_venn_diagram2.pdf",width=4,height=4); dev.off() }
```

*Hotspot analyses*: sitednds and codondnds are relatively new functions in the dndscv package that perform enrichment tests for mutations at the level of individual sites or codons. These two methods can be very sensitive to detect selection at the level of single sites or codons within a gene, but they can also be prone to false positives due to recurrent artefacts.

```
dndsout_nondc = dndscv(unique(mutations[,1:5]), gene_list = targetgenes, max_muts_per_gene_per_sample = Inf, max_coding_muts_per_sample = Inf, constrain_wnon_wspl = T, mingenecovs = 0, kc = newkc, outmats = T, onesided = T, cv = dndscovs, refdb = RefCDS, maxcovs = 10, use_indel_sites = use_indel_sites) # Excluding substitution drivers from the indel model
hotspots_allsites = sitednds(dndsout_nondc, gene_list = targetgenes, method = "LNP")
data("knownhotspots_hg19", package="dndscv")
known_hotspots = known_hotspots[sapply(strsplit(known_hotspots, split=":"), function(x) x[5]) %in% targetgenes] # List of known hotspots in the targetgenes
hotspots_siterht = sitednds(dndsout_nondc, site_list = known_hotspots, method = "LNP")

numsites_pergene1 = sort(table(hotspots_allsites$recursites[hotspots_allsites$recursites$qval<0.01 & hotspots_allsites$recursites$impact!="Synonymous","gene"]),descending=T)
numsites_pergene2 = sort(table(hotspots_siterht$recursites[hotspots_siterht$recursites$qval<0.01 & hotspots_siterht$recursites$impact!="Synonymous","gene"]),descending=T)

# Saving sitednds results as files
write.table(hotspots_allsites$recursites[hotspots_allsites$recursites$qval<0.01,], file="Sitednds_noRHT_significant_sites.tsv", 
            col.names=T, row.names=F, sep="\t", quote=F)
write.table(hotspots_siterht$recursites[hotspots_allsites$recursites$qval<0.01,], file="Sitednds_siteRHT_significant_sites.tsv",
            col.names=T, row.names=F, sep="\t", quote=F)
```

### Driver analyses in relevant cancer types

To look for driver mutations in head and neck cancers, we use the MC3 calls from the TCGA HNSC dataset. This analysis could be further restricted to oral cancers, including the buccal mucosa, alveolar ridge, floor of mouth, hard palate, oral cavity, and the anterior two-thirds of the tongue, following the approach used in PMID:34422810 (annotation codes https://www.ncri.ie/html/icdo3sites, oral codes C01-C06). However, the current analysis is already limited by the sparsity of the cancer data. An alternative approach would be to combine multiple datasets from related squamous cancers from TCGA, for example combining the HNSC, ESCA and LUSC TCGA datasets (using the MC3 calls).

```
tcga_mutations = read.table(tcga_HNSC_calls_file, header=1, sep="\t", stringsAsFactors=F)
dndsout_tcga = dndscv(tcga_mutations, gene_list = targetgenes, max_muts_per_gene_per_sample = Inf, max_coding_muts_per_sample = Inf, constrain_wnon_wspl = T, mingenecovs = 0, onesided = T, cv = dndscovs, refdb = RefCDS)
aux = as.vector(dndsout_tcga$sel_cv$gene_name[dndsout_tcga$sel_cv$qallsubs_cv<0.01])
dndsout_tcga = dndscv(tcga_mutations, gene_list = targetgenes, max_muts_per_gene_per_sample = Inf, max_coding_muts_per_sample = Inf, constrain_wnon_wspl = T, mingenecovs = 0, onesided = T, kc = aux, outmats = T, cv = dndscovs, refdb = RefCDS) # Excluding substitution drivers from the indel model
tcga_drivers = dndsout_tcga$sel_cv[dndsout_tcga$sel_cv$qglobalpos_cv<0.01, "gene_name"]

# Venn diagram
if (runman) { dev.new(width=4, height=4) }
d = list(TwinsUK=drivers, TCGA_HNSC=tcga_drivers)
ggvenn(d, fill_color = c("#0073C2FF", "#CD534CFF"), stroke_size = 0.5, set_name_size = 4)
```

```
if (runman) { dev.copy(pdf,"Buccal_drivers_vs_TCGA_HNSC_venn_diagram.pdf",width=4,height=4); dev.off() }
# Differentially selected genes
paste(setdiff(tcga_drivers, drivers), collapse = " ,")
```

```
## [1] "PIK3CA ,HRAS ,CASP8 ,CDKN2A.p16INK4a ,NSD1 ,KMT2D ,CDKN2A.p14arf ,HLA-A ,TGFBR2 ,FBXW7 ,NFE2L2 ,PTEN ,B2M ,SMAD4 ,KEAP1 ,RASA1 ,CYLD ,RB1 ,CTCF"
```

```
paste(setdiff(drivers, tcga_drivers), collapse = " ,")
```

```
## [1] "CHEK2 ,NOTCH2 ,ATM ,DNMT3A ,KDM5C ,TP63 ,TET2 ,ASXL1 ,RBM10 ,KMT2C ,ZFP36L2 ,SETD2 ,ARID1B ,MGA ,FUBP1 ,ARID1A ,PPM1D ,NOTCH3 ,BCORL1 ,ZFP36L1 ,CCND1 ,PAX9 ,SF3B1 ,EIF1AX ,PIK3R1 ,STAG2 ,SMARCB1 ,KLF5 ,FGFR3 ,RARG ,ETV6 ,FOXP1 ,EGFR ,SPOP ,CIC ,ARID5B"
```

As a more sensitive analysis of selection in cancer, we can use RHT on oral epithelium drivers

```
dndsout_tcga_rht = dndsout_tcga$sel_cv[dndsout_tcga$sel_cv$gene_name %in% drivers,]
dndsout_tcga_rht$qglobalpos_rht = p.adjust(dndsout_tcga_rht$pglobalpos_cv, method="BH")
message(sprintf("Genes RHT qval<0.1 in cancer: %s", paste(dndsout_tcga_rht$gene[dndsout_tcga_rht$qglobalpos_rht<0.1], collapse=", ")))
```

```
## Genes RHT qval<0.1 in cancer: TP53, NOTCH1, FAT1, AJUBA, HLA-B, EPHA2, RAC1, EP300, ZNF750, KDM6A, CUL3, RHOA, ARID2, KMT2C, RARG, ASXL1, ZFP36L2, NOTCH2, FGFR3, ARID1A, PIK3R1
```

### Driver analyses in normal blood

Driver discovery in the targeted Nanoseq blood data.

```
gene2dc_blood = rowSums(read.table(dcpergene_blood_file, header=1, sep="\t", stringsAsFactors=F))
m = unique(bloodmuts[,1:5]) # Unique mutations per sample (note that the dndscv annotation of the calls could have duplicated some calls)
dndsout_blood = dndscv(m, gene_list = targetgenes, max_muts_per_gene_per_sample = Inf, max_coding_muts_per_sample = Inf, constrain_wnon_wspl = T, mingenecovs = 0, dc = gene2dc_blood/mean(gene2dc_blood), onesided = T, cv = dndscovs, refdb = RefCDS, maxcovs = 10, use_indel_sites = use_indel_sites)
newkc_blood = as.vector(dndsout_blood$sel_loc$gene_name[dndsout_blood$sel_loc$qall_loc<0.01])
dndsout_blood = dndscv(m, gene_list = targetgenes, max_muts_per_gene_per_sample = Inf, max_coding_muts_per_sample = Inf, constrain_wnon_wspl = T, mingenecovs = 0, dc = gene2dc_blood/mean(gene2dc_blood), kc = newkc_blood, outmats = T, onesided = T, cv = dndscovs, refdb = RefCDS, maxcovs = 10, use_indel_sites = use_indel_sites) # Excluding substitution drivers from the indel model

# Writing the dNdScv output table to a file (Extended Data Table)
write.table(dndsout_blood$sel_cv[order(dndsout_blood$sel_cv$psubpos_cv),], file = "EDT3_dNdScv_output_blood_tab.tsv", row.names=F, col.names=T, sep="\t", quote=F)
```

Adding protein-coding annotations to the blood mutation table.

```
a = dndsout_blood$annotmuts
a$mstr = paste(a$sampleID,a$chr,a$pos,a$mut,sep=":")
bloodmuts = bloodmuts[, setdiff(colnames(bloodmuts),colnames(a)[-(1:5)])]
bloodmuts$mstr = paste(bloodmuts$sampleID,bloodmuts$chr,bloodmuts$pos,bloodmuts$mut,sep=":")
bloodmuts = merge(bloodmuts, a[,6:ncol(a)], by="mstr", all.x = T)
bloodmuts$mstr = NULL # Removing the mstr field
write.table(bloodmuts, file="TwinsUK_annotated_mutations_blood.tsv", col.names=T, row.names=F, sep="\t", quote=F)
```

We can then define blood drivers using the same approach as in the buccal data.

```
# Lists of significant genes using the dNdSloc and dNdScv one-sided tests
drivers1_blood = dndsout_blood$sel_loc[dndsout_blood$sel_loc$qpos_loc<0.01, "gene_name"]
drivers2_blood = dndsout_blood$sel_cv[dndsout_blood$sel_cv$qglobalpos_cv<0.01, "gene_name"]
drivers3_blood = dndsout_blood$sel_cv[dndsout_blood$sel_cv$qsubpos_cv<0.01, "gene_name"]
#drivers_blood = base::intersect(base::intersect(drivers1_blood,drivers2_blood),drivers3_blood)
drivers_blood = drivers3_blood # We opt to use qsubpos_cv<0.01 as the intersection is dominated by dNdSloc, which is underpowered in the blood dataset, given its smaller size.
# Venn diagram
if (runman) { dev.new(width=4, height=4) }
d = list(dNdSloc=drivers1_blood, dNdScvglobal=drivers2_blood, dNdScvsubs=drivers3_blood)
ggvenn(d, fill_color = c("#0073C2FF", "#EFC000FF", "#868686FF"), stroke_size = 0.5, set_name_size = 4)
```

```
if (runman) { dev.copy(pdf,"Blood_drivers_venn_diagram.pdf",width=4,height=4); dev.off() }
```

Hotspot analyses in blood.

```
dndsout_nondc_blood = dndscv(unique(bloodmuts[,1:5]), gene_list = targetgenes, max_muts_per_gene_per_sample = Inf, max_coding_muts_per_sample = Inf, constrain_wnon_wspl = T, mingenecovs = 0, kc = newkc_blood, outmats = T, onesided = T, cv = dndscovs, refdb = RefCDS, maxcovs = 10, use_indel_sites = use_indel_sites) # Excluding substitution drivers from the indel model

hotspots_allsites_blood = sitednds(dndsout_nondc_blood, gene_list = targetgenes, method = "LNP")
hotspots_siterht_blood = sitednds(dndsout_nondc_blood, site_list = known_hotspots, method = "LNP")

numsites_pergene1_blood = sort(table(hotspots_allsites_blood$recursites[hotspots_allsites_blood$recursites$qval<0.01 & hotspots_allsites_blood$recursites$impact!="Synonymous","gene"]),descending=T)
numsites_pergene2_blood = sort(table(hotspots_siterht_blood$recursites[hotspots_siterht_blood$recursites$qval<0.01 & hotspots_siterht_blood$recursites$impact!="Synonymous","gene"]),descending=T)

# Saving sitednds results as files
write.table(hotspots_allsites_blood$recursites[hotspots_allsites_blood$recursites$qval<0.01,], file="Sitednds_noRHT_significant_sites_blood.tsv", 
            col.names=T, row.names=F, sep="\t", quote=F)
write.table(hotspots_siterht_blood$recursites[hotspots_siterht_blood$recursites$qval<0.01,], file="Sitednds_siteRHT_significant_sites_blood.tsv",
            col.names=T, row.names=F, sep="\t", quote=F)
```

### Plotting the gene-level driver results

Subfunction to generate standard driver plots, similar to Martincorena et al, 2015 (PMID: 25999502) and 2018 (PMID: 30337457), and Lawson et al, 2020 (PMID: 33004514).

```
# Driver plot
# The driverdensity_sampleIDs is an optional vector with the IDs of the samples to include for the estimation of driver % (e.g. individuals of a certain age and/or meeting a minimum duplex depth)
# gene2dc allows to input a different coverage vector (such as gene2dc_blood for the blood dataset)
# logscale refers to the driver density plot

driverplot = function(genes2plot, muts, dndsout, dndsout_tcga = NULL, max_genes = Inf, sortbyfreq = T, onlysignifdnds = T, vafdnds = T, driverdensity_sampleIDs = NULL, gene2dc = gene2dc, plotwidth = 10, plotheight = 10, plotfilename = "Driver_plot.pdf", logscale = T, plotmutsperdonor = F, allindels = T) {
  
  if (runman) { dev.new() }
  par(mfrow=c(5,1), mar=c(3,5.5,2,2))
  
  # a. Mutations observed
  nmuts = as.matrix(dndsout$sel_cv[,2:6])
  rownames(nmuts) = dndsout$sel_cv$gene_name
  if (sortbyfreq) {
    genes2plot = names(sort(rowSums(nmuts)[genes2plot],decreasing=T)) # Sorting the genes in decreasing order of their number of mutations
  }
  if (max_genes<length(genes2plot)) {
    genes2plot = head(genes2plot,max_genes) # We select the first N genes
  }
  nmuts = nmuts[genes2plot,]
  subs_per_gene = t(nmuts)
  rownames(subs_per_gene) = c("Synonymous","Missense","Nonsense","Splice","Indels")
  if (allindels) { # We annotate the total number of indels per gene (not just unique indel sites from sel_cv)
    subs_per_gene["Indels",] = setNames(dndsout$geneindels$n_ind, dndsout$geneindels$gene_name)[genes2plot]
  }
  colvec = c("grey70","cadetblue","darkorchid4","darkorchid2","chocolate3")
  pos = barplot(subs_per_gene, las=2, col=colvec, border=NA, ylim=c(0, max(colSums(subs_per_gene))*1.1), ylab="Total mutations")
  legend("topright",y=max(apply(subs_per_gene,2,sum))*1.09,legend=rownames(subs_per_gene),fill=colvec,border=NA,box.col=NA)
  verticalbar = mean(pos[(0:1)+length(drivers)])
  abline(v=verticalbar)
  if (plotmutsperdonor) { # Adding a second axis with the mean number of mutations per donor
    numsamples = length(unique(dndsout$annotmuts$sampleID))
    ticks = pretty(1:max(colSums(subs_per_gene))/numsamples)
    axis(4, at=ticks*numsamples, labels=ticks, las=1)
    #mtext("Mean mutations/donor", side = 4)
  }

  # b. dN/dS ratios from the dNdScv model
  obsw = as.matrix(dndsout$sel_cv[,c("wmis_cv","wnon_cv","wind_cv")])
  rownames(obsw) = dndsout$sel_cv$gene_name
  colnames(obsw) = c("Missense","Nonsense+splice","Indels")
  obsw = obsw[genes2plot,]
  if (onlysignifdnds) {
      obsp = as.matrix(dndsout$sel_cv[,c("pmis_cv","ptrunc_cv","pindpos_cv")]); rownames(obsp) = dndsout$sel_cv$gene_name; obsp = obsp[genes2plot,]; obsw[obsp>0.01] = NA # Masking out P>0.05
  }
  pos = barplot(t(obsw), beside=T, las=2, col=c("cadetblue","darkorchid3","chocolate3"), border=NA, ylim=c(0,max(obsw,na.rm=T)+10), ylab="dN/dS ratios")
  legend("topright",y=max(apply(obsw,2,sum))*1.09,legend=colnames(obsw),fill=c("cadetblue","darkorchid3","chocolate3"),border=NA,box.col=NA)
  abline(h=1, col="grey")
  
  # c. % of mutant cells
  #    This is based on some assumptions:
  #    1. The calculation will be restricted to samples listed in the driverdensity_sampleIDs argument.
  #    2. The values are averages across all donors weighted by duplex coverage.
  #    3. Values are bounded between sum(duplexVAFs) and sum(2*duplexVAFs), as explained in Martincorena et al 2018 for standard VAFs.
  #    4. To account for VAFs (clone sizes), dN/dS ratios for significant genes will be recalculated weighting mutations by their frequency.
  #       This should provide an unbiased point estimate for the purpose of this analysis, but CI95% cannot be calculated with geneci.
  
  if (vafdnds == T)  {
    
    maux = muts[rep(1:nrow(muts), muts$times_called), ] # Mutation table with each mutation represented N times (N = times_called)
    if (length(driverdensity_sampleIDs)>0) {
      maux = maux[which(maux$sampleID %in% driverdensity_sampleIDs), ]
      missing_samples = setdiff(driverdensity_sampleIDs, unique(muts$sampleID)); if (length(missing_samples)>0) { warning(sprintf("Missing samples in the mutation file will be excluded: %s", paste(missing_samples, collapse = ",")))} # Warning about missing samples requested in the optional argument
    }
    maux$sampleID = paste(maux$sampleID, 1:nrow(maux), sep=":!&") # Adding a numeric suffix to sampleID to avoid removal of redundant mutations by dndscv
    
    message("Calculating VAF-weighted dN/dS ratios to estimate the density of driver mutations per cell")
    dndsaux = dndscv(maux, gene_list = targetgenes, max_muts_per_gene_per_sample = Inf, max_coding_muts_per_sample = Inf, constrain_wnon_wspl = T, mingenecovs = 0, dc = gene2dc/mean(gene2dc), kc = newkc, outmats = T, onesided = T, cv = dndscovs, refdb = RefCDS, maxcovs = 10, use_indel_sites = use_indel_sites) # Excluding substitution drivers from the indel model

    # Removing the numeric suffix added above
    dndsaux$annotmuts$sampleID = sapply(strsplit(dndsaux$annotmuts$sampleID, split=":!&"), function(x) x[1])
    maux$sampleID = sapply(strsplit(maux$sampleID, split=":!&"), function(x) x[1])
    dndsaux$annotmuts = unique(dndsaux$annotmuts)
    maux = unique(maux)
    
  } else {
    
    if (length(driverdensity_sampleIDs)>0) {
      maux = maux[which(maux$sampleID %in% driverdensity_sampleIDs), ]
      message("Calculating dN/dS ratios restricted to the samples of interest to estimate the density of driver mutations per cell")
      dndsaux = dndscv(maux, gene_list = targetgenes, max_muts_per_gene_per_sample = Inf, max_coding_muts_per_sample = Inf, constrain_wnon_wspl = T, mingenecovs = 0, dc = gene2dc/mean(gene2dc), kc = newkc, outmats = T, onesided = T, cv = dndscovs, refdb = RefCDS, maxcovs = 10, use_indel_sites = use_indel_sites) # Excluding substitution drivers from the indel model
      missing_samples = setdiff(driverdensity_sampleIDs, unique(muts$sampleID)); if (length(missing_samples)>0) { warning(sprintf("Missing samples in the mutation file will be excluded: %s", paste(missing_samples, collapse = ",")))} # Warning about missing samples requested in the optional argument
    } else {
      dndsaux = dndsout
    }
  }
  
  # Adding duplex VAFs and cell fraction information to the annotmuts object (cell fraction should be =2*duplexVAF for all mutations except X-chr or Y-chr mutations in males)
  duplexvaf = setNames(maux$duplex_vaf, paste(maux$sampleID,maux$chr,maux$pos,maux$mut,sep=":"))
  cf = setNames(maux$cellfraction, paste(maux$sampleID,maux$chr,maux$pos,maux$mut,sep=":"))
  m = unique(dndsaux$annotmuts)
  m$duplex_vaf = duplexvaf[paste(m$sampleID,m$chr,m$pos,m$mut,sep=":")]
  m$cellfraction = cf[paste(m$sampleID,m$chr,m$pos,m$mut,sep=":")]
  
  # We then calculate the sum(cellfractions) for every mutation type across all samples and genes, and multiply each mutation class by the relevant driver fraction (w-1/(w))
  cellfraction = array(NA, dim = c(length(genes2plot),8), dimnames = list(genes2plot, c("mis","non","spl","ind","mislow","nonlow","spllow","indlow")))
  numsamples = length(unique(dndsaux$annotmuts$sampleID)) # This assumes that only samples with mutations should be considered in the calculation (in case some samples in driverdensity_sampleIDs are not in the dataset)
  for (j in 1:length(genes2plot)) {
    aux = m[which(m$gene==genes2plot[j]),c("impact","cellfraction","duplex_vaf")]
    cellfraction[j,c("mis","mislow")] = colSums(aux[aux$impact=="Missense",2:3]) / numsamples
    cellfraction[j,c("non","nonlow")] = colSums(aux[aux$impact=="Nonsense",2:3]) / numsamples
    cellfraction[j,c("spl","spllow")] = colSums(aux[aux$impact=="Essential_Splice",2:3]) / numsamples
    cellfraction[j,c("ind","indlow")] = colSums(aux[aux$impact=="no-SNV",2:3]) / numsamples
  }

  # Approximate fraction per driver gene
  d = dndsaux$sel_cv[which(dndsaux$sel_cv$gene_name %in% genes2plot),]
  driv_fract = (d[,7:10]-1)/d[,7:10]
  rownames(driv_fract) = d$gene_name
  driv_fract[driv_fract<0] = 0
  driv_fract = driv_fract[genes2plot,]

  # Estimated driver density per gene (% of cells with a driver mutation in oral epithelium)
  driverdens = cellfraction * cbind(driv_fract,driv_fract) * 100
  driverdens_bounds = cbind(up=rowSums(driverdens[,1:4]),low=rowSums(driverdens[,5:8]))
  aux = t(cbind(driverdens_bounds[,2],driverdens_bounds[,1]-driverdens_bounds[,2]))
  
  if (logscale == T) {
    pos = barplot(aux, las=2, col=c("white","indianred3"), border=NA, ylab="% cells driver mutation", log="y")
  } else {
    pos = barplot(aux, las=2, col=c("white","indianred3"), border=NA, ylab="% cells driver mutation")
  }

  # d. % of TCGA HNSC tumours with a non-synonymous SNV or indel in each gene
  if (!is.null(dndsout_tcga)) {
    num_tcga = length(unique(dndsout_tcga$annotmuts$sampleID)) # Number of tumours sequenced
    num_NStumours = data.frame(gene=genes2plot, numtum = NA, fract = NA, cilow = NA, cihigh = NA)
    for (j in 1:length(genes2plot)) {
      num_NStumours[j,2] = length(unique(dndsout_tcga$annotmuts$sampleID[which(dndsout_tcga$annotmuts$gene==genes2plot[j] & dndsout_tcga$annotmuts$impact!="Synonymous")])) # Tumours with >=1 nonsyn SNV or indel in the gene
      num_NStumours[j,3] = num_NStumours[j,2] / num_tcga * 100
      num_NStumours[j,4:5] = binom.test(x = num_NStumours[j,2], n = num_tcga)$conf.int * 100
    }
    pos = barplot(num_NStumours$fract, las=2, col=c("grey30"), border=NA, ylab="% mutant tumours", ylim=c(0,max(num_NStumours$cihigh)))
    segments(x0=pos, y0=num_NStumours$cilow, y1=num_NStumours$cihigh)
    points(x=pos, y=driverdens_bounds[,1], col="darkorange")
  }

  # e. Unbiased BAM VAFs for the non-synonymous mutations in each gene
  muts$bam_vaf_corr = (muts$bam_mut-muts$times_called)/(muts$bam_cov-muts$duplex_cov)
  muts$bam_vaf_corr[muts$bam_vaf_corr<0] = NA
  m = muts[which(muts$gene %in% genes2plot & muts$impact %in% c("Missense","Nonsense","Essential_Splice")),]
  #m$bam_vaf_corr_cut = cut(m$bam_vaf_corr, breaks = c(-Inf,0,1e-3,1e-2,0.05,1), include.lowest = T)
  m$bam_vaf_corr_cut = cut(m$bam_vaf_corr, breaks = c(0,1e-4,1e-3,1e-2,0.05,1), include.lowest = T)
  cuts = as.vector(sort(unique(m$bam_vaf_corr_cut[!is.na(m$bam_vaf_corr_cut)]))) # Names of each range of values
  vafdist = array(NA, dim=c(length(genes2plot),length(cuts)), dimnames = list(genes2plot,cuts))
  for (j in 1:length(genes2plot)) {
    f = table(m$bam_vaf_corr_cut[m$gene==genes2plot[j]])[cuts]
    vafdist[j,] = f / sum(f)
  }
  
  colvec = c("#482677FF","#2D708EFF","#29AF7FFF","#B8DE29FF","red")
  #colvec = c("deepskyblue4","deepskyblue1","blueviolet","darkorange","red")
  #colvec = brewer.pal(n = 5, name = "BlOrRd")
  pos = barplot(t(vafdist), las=2, col=colvec, border=NA, ylab="VAFs", xlab="")

  if (runman) { dev.copy(pdf, plotfilename, width=plotwidth, height=plotheight); dev.off() }
  return(driverdens_bounds)
  
  # Additional plot: Ratio of mutation frequency in tumours vs normal cells
  # Notice that this is only an approximation, as we are not correcting the tumour fraction by driver probability 
  # due to the sparsity of cancer data. And we are not calculating CI95% for the driver density in normal epithelium.
  # And we are also taking the heterozygous estimate for the normal epithelium.
  
  if (0) {
    dev.new(width=10,height=4)
    r = num_NStumours[,c("fract","cilow","cihigh")] / driverdens_bounds[,1]
    rownames(r) = num_NStumours$gene
    plot(x=1:nrow(r), y=r$fract, las=2, ylab="Approx ratio tumour/normal", ylim=c(min(r$cilow),max(r$cihigh)), log="y", xaxt = "n", pch=19, xlab="")
    axis(1, at = 1:nrow(r), labels = num_NStumours$gene, las=2)
    segments(x0=1:nrow(r), y0=r$cilow, y1=r$cihigh)
    abline(h=1, col="cadetblue")
    dev.copy(pdf,"Approximate_ratio_tumour_vs_normal.pdf",width=10,height=4); dev.off()
  }
}
```

Driver plots: number of mutations per gene, dN/dS ratios, % of mutant epithelium, comparison to cancer, and VAF distributions.

```
# 1. Driver plot with all driver genes
driverdensity_sampleIDs = metadata$pd[which(metadata$chemo==F & metadata$AGE>=65 & metadata$AGE<=85)]
driverdens_buccal = driverplot(genes2plot=drivers, muts=mutations, dndsout=dndsout, dndsout_tcga=dndsout_tcga, max_genes = Inf, sortbyfreq = T, onlysignifdnds = T, vafdnds = T, driverdensity_sampleIDs = driverdensity_sampleIDs, gene2dc = gene2dc, plotwidth = 10, plotheight = 10, logscale = T, plotfilename = "Fig2_driver_plot_alldrivers.pdf", plotmutsperdonor = T)
```

```
# 2. Driver plot with the top 20 genes
topdrivers = names(sort(driverdens_buccal[,1], decreasing=T))[1:20]
kk = driverplot(genes2plot=topdrivers, muts=mutations, dndsout=dndsout, dndsout_tcga=dndsout_tcga, max_genes = Inf, sortbyfreq = T, onlysignifdnds = T, vafdnds = T, driverdensity_sampleIDs = driverdensity_sampleIDs, gene2dc = gene2dc, plotwidth = 5, plotheight = 10, logscale = F, plotfilename = "Fig2_driver_plot_topdrivers.pdf", plotmutsperdonor = T)
```

Plotting the number of significant sites with sitednds.

```
# Combining the table of significant sites from allsites and siterht
#hot = unique(rbind(hotspots_allsites$recursites[hotspots_allsites$recursites$qval<0.01,c("gene","aachange","impact","freq")], hotspots_siterht$recursites[hotspots_siterht$recursites$qval<0.01,c("gene","aachange","impact","freq")]))

# Using only the gene-wide analysis in sitednds, and collapsing the reporting by unique aminoacid changes
hot = unique(hotspots_allsites$recursites[hotspots_allsites$recursites$qval<0.01,c("gene","aachange","impact","freq")])
genefreq = sort(table(hot$gene), decreasing = T) # Sorting genes by their number of significant sites
genes2plot = names(genefreq[genefreq>=4]) # Plotting genes with at least 5 significant hotspots

if (runman) { dev.new(width=10, height=3) }
par(mfrow=c(1,2))

# a. Number of significant sites
hotimpacts = sapply(split(hot,f=hot$gene), function(x) table(x$impact)[c("Missense","Nonsense","Essential_Splice","Synonymous")])
hotimpacts[is.na(hotimpacts)] = 0
rownames(hotimpacts) = c("Missense","Nonsense","Essential_Splice","Synonymous")
hotimpacts = hotimpacts[,genes2plot]

colvec = c("cadetblue","darkorchid4","darkorchid2","grey70")
h = barplot(hotimpacts, col=colvec, las=2, ylim=c(0,ceiling(max(colSums(hotimpacts))/100)*115), ylab="Number of significant sites", border = NA)
text(x=h, y=colSums(hotimpacts)+15, labels=colSums(hotimpacts), srt=90, adj=c(0,0.5))
legend(x=mean(h), y=max(colSums(hotimpacts)), legend=rownames(hotimpacts), fill=colvec, border=NA, box.col=NA)

# b. Number of mutations observed in significant sites
hot = hot[rep(1:nrow(hot), times=hot$freq),] # Repeating each row as many times as each mutation was observed 
hotimpacts = sapply(split(hot,f=hot$gene), function(x) table(x$impact)[c("Missense","Nonsense","Essential_Splice","Synonymous")])
hotimpacts[is.na(hotimpacts)] = 0
rownames(hotimpacts) = c("Missense","Nonsense","Essential splice","Synonymous")
hotimpacts = hotimpacts[,genes2plot]

colvec = c("cadetblue","darkorchid4","darkorchid2","grey70")
fraction_inhotspot = colSums(hotimpacts)/setNames(rowSums(dndsout$sel_cv[,2:5]), dndsout$sel_cv[,1])[genes2plot] * 100 # % of coding mutations in the gene that occurred in significant sites by sitednds
h = barplot(hotimpacts, col=colvec, las=2, ylim=c(0,ceiling(max(colSums(hotimpacts))/100)*115), ylab="Number of mutations in significant sites", border = NA)
labls = paste(colSums(hotimpacts), " (", round(fraction_inhotspot, digit=0), "%)", sep="")
text(x=h, y=colSums(hotimpacts)+200, labels=labls, srt=90, adj = c(0,0.5))
legend(x=mean(h), y=max(colSums(hotimpacts)), legend=rownames(hotimpacts), fill=colvec, border=NA, box.col=NA)
```

```
if (runman) { dev.copy(pdf,"Sitednds_number_significant_hotspots.pdf",width=10,height=3); dev.off() }
```

Heatmap of the number of mutations per gene per donor.

```
# Table of non-synonymous mutations per gene per donor in selected driver genes
genes2plot = topdrivers
s = unique(mutations$sampleID)
aux = mutations[which(mutations$gene %in% topdrivers & mutations$impact!="Synonymous"),]
aux = split(aux, f=aux$sampleID)
nmuts_ns = t(sapply(aux, function(x) table(x$gene)[genes2plot]))
nmuts_ns[is.na(nmuts_ns)] = 0
colnames(nmuts_ns) = topdrivers

# Heatmap of all samples with >=150 non-synonymous mutations in the selected driver genes
x = nmuts_ns[rowSums(nmuts_ns)>=150,]
x = x[order(rowSums(x), decreasing = T),]
x[x>100] = 100
if (runman) { dev.new(width=5, height=7) }
colpal = colorRampPalette(c("#482677FF","#2D708EFF","#29AF7FFF","#B8DE29FF","orange","darkorange","indianred","indianred4"))(n = 101)
heatmap.2(x=x, col=colpal, density.info="none", trace="none", margins = c(6,7), scale = "none", dendrogram="none", Colv="NA", cexCol = 0.5, cexRow = 0.4, keysize = 1.5, lhei = c(1,5), key.title = "", key.xlab = "Number non-synonymous mutations", key.par=list(mar=c(4,2,4,2), cex=0.6, cex.lab=0.6, cex.axis=0.6))
```

```
if (runman) { dev.copy(pdf, file = "Heatmap_nonsyn_muts_per_gene_per_donor.pdf", width=5, height=7); dev.off() }
```

Ranked dot plot of the number of mutations per gene per donor.

```
# Ranked dot plot
selected_samples = metadata$pd[metadata$duplex_cov>=1000 & metadata$AGE>=65 & metadata$AGE<=85]
if (runman) { dev.new(width=5, height=7) }
colvec = c("hotpink4","darkorchid3","chocolate","darkslategray3","bisque3","darkolivegreen4")
plot(1:length(selected_samples), sort(nmuts_ns[selected_samples,1]), col=colvec[1], xlab="Sample rank", ylab="Non-synonymous per gene per donor", log="y", pch=16, las=2, ylim=c(1,max(nmuts_ns[selected_samples,1:6])))
points(1:length(selected_samples), sort(nmuts_ns[selected_samples,2]), col=colvec[2], pch=16)
points(1:length(selected_samples), sort(nmuts_ns[selected_samples,3]), col=colvec[3], pch=16)
points(1:length(selected_samples), sort(nmuts_ns[selected_samples,4]), col=colvec[4], pch=16)
points(1:length(selected_samples), sort(nmuts_ns[selected_samples,5]), col=colvec[5], pch=16)
points(1:length(selected_samples), sort(nmuts_ns[selected_samples,6]), col=colvec[6], pch=16)
legend(x=1, y=max(max(nmuts_ns[selected_samples,1:6])), legend=colnames(nmuts_ns)[1:6], pch=16, border=NA, box.col=NA, col=colvec, cex=0.7, bg="transparent")
```

```
if (runman) { dev.copy(pdf, file = "Dotplot_muts_per_gene_per_donor.pdf", width=4, height=4); dev.off() }
```

### Exome-wide dN/dS results

Running dNdScv on the whole-exome Nanoseq data.

```
# Driver discovery
exomemuts = read.table(exomemuts_file, header=1, sep="\t", stringsAsFactors=F)
exomedc = read.table(exomedc_file, header=1, sep="\t", stringsAsFactors=F)
gene2dc_exome = setNames(exomedc$dplx_cov_mean, exomedc$gene)
exome_genes = intersect(exomedc$gene, sapply(RefCDS, function(x) x$gene_name)) # List of genes in the RefCDS with coverage information

m = unique(exomemuts[,1:5]) # Unique mutations per sample (note that the dndscv annotation of the calls could have duplicated some calls)
dndsout_exome = dndscv(m, gene_list = exome_genes, max_muts_per_gene_per_sample = Inf, max_coding_muts_per_sample = Inf, constrain_wnon_wspl = T, mingenecovs = 0, dc = gene2dc_exome/mean(gene2dc_exome), onesided = T, kc = newkc, cv = dndscovs, refdb = RefCDS)

# Global dN/dS for passenger genes using the whole-exome data
dndsout_exome_passengers = dndscv(m, gene_list = setdiff(exome_genes, drivers), max_muts_per_gene_per_sample = Inf, max_coding_muts_per_sample = Inf, constrain_wnon_wspl = T, mingenecovs = 0, dc = gene2dc_exome/mean(gene2dc_exome), onesided = T, kc = newkc, outp=1, cv = dndscovs, refdb = RefCDS)

# Global dN/dS for driver genes using the targeted data
dndsout_drivers = dndscv(unique(mutations[,1:5]), gene_list = drivers, max_muts_per_gene_per_sample = Inf, max_coding_muts_per_sample = Inf, constrain_wnon_wspl = T, mingenecovs = 0, dc = gene2dc/mean(gene2dc), onesided = T, kc = newkc, outp=1, cv = dndscovs, refdb = RefCDS)

# Global dN/dS for essential genes using the targeted data: we define essential genes as those classified as essential by CRISPR that are in the targeted panel and NOT in the list of significant drivers
essential = read.table(essentialgenes_file, header=1, sep="\t", stringsAsFactors = F)[,1]
essential = sapply(strsplit(essential, split=" "), function(x) x[1])
essential_passengers = setdiff(intersect(targetgenes, essential), drivers)
dndsout_essential = dndscv(unique(mutations[,1:5]), gene_list = essential_passengers, max_muts_per_gene_per_sample = Inf, max_coding_muts_per_sample = Inf, constrain_wnon_wspl = T, mingenecovs = 0, dc = gene2dc/mean(gene2dc), onesided = T, kc = newkc, outp=1, cv = dndscovs, refdb = RefCDS)
```

Plotting the global dN/dS estimates for drivers, putative passengers, and essential genes.

```
# Plotting the global dN/dS results
g1 = dndsout_exome_passengers$globaldnds[c("wmis","wnon"),-1]
g2 = dndsout_drivers$globaldnds[c("wmis","wnon"),-1]
g3 = dndsout_essential$globaldnds[c("wmis","wnon"),-1]

if (runman) { dev.new(width=3.5, height=4) }
colvec = c("cadetblue","darkorchid4")
h = barplot(cbind(g1[,1],g2[,1],g3[,1]), beside = T, ylim=c(0,max(g2)), las=1, names.arg = c("Exome-wide\npassengers","Drivers","Essential genes\n(targeted)"), col=colvec, border=NA, ylab = "dN/dS ratios")
segments(x0=h, y0=cbind(g1[,2],g2[,2],g3[,2]), y1=cbind(g1[,3],g2[,3],g3[,3]))
legend(x = min(h), y = max(g2), legend = c("Missense","Nonsense"), border=NA, box.col=NA, pch=15, col=colvec)
abline(h=1, lty=1)
```

```
if (runman) { dev.copy(pdf, file = "Global_dNdS_passengers_drivers_essential.pdf", width=3.5, height=4); dev.off() }
```

Estimating the total number of driver mutations in the targeted dataset. We do so here by using the excess of non-synonymous mutations and indels in 2 ways:

1. Calculating global dN/dS ratios for all genes in the panel (excluding positively selected genes from the indel model)
2. Calculating global dN/dS ratios only for the genes found under significant positive selection.

```
# SNVs option 1. Using the global dN/dS ratios for substitutions
nvec = colSums(dndsout$genemuts[, c("n_mis","n_non","n_spl")])
w = dndsout$globaldnds[1:3,2:4]
est_snv_drivers = colSums((w-1)/w * nvec)

# SNVs option 2. Using the obs and exp number of mutations per gene for the list of positively-selected genes
nvec = colSums(dndsout$genemuts[dndsout$genemuts$gene_name %in% drivers, c("n_mis","n_non","n_spl")])
rateadj = dndsout$genemuts$exp_syn_cv / dndsout$genemuts$exp_syn
expvec = colSums((dndsout$genemuts[,c("exp_mis","exp_non","exp_spl")] * rateadj)[dndsout$genemuts$gene_name %in% drivers,])
est_snv_drivers2 = sum(nvec-expvec)

# Indels
vec = colSums(dndsout$geneindels[dndsout$genemuts$gene_name %in% drivers, c("n_indused","exp_indcv")])
est_ind_drivers = vec[1]-vec[2]

message(sprintf("Estimated number of driver mutations: %0.1f", est_snv_drivers2 + est_ind_drivers))
```

```
## Estimated number of driver mutations: 62286.7
```

### Negative selection analyses

One-sided negative selection tests from dNdScv.

```
negsel1 = dndsout$sel_loc[dndsout$sel_loc$qneg_loc<0.05, ]
negsel2 = dndsout$sel_cv[dndsout$sel_cv$qsubneg_cv<0.05, ]
print(negsel2[,c(1:10,15,17,22,28)])
```

```
##     gene_name n_syn n_mis n_non n_spl n_ind  wmis_cv   wnon_cv   wspl_cv
## 204     SF3B1   149   715     5     5    37 1.449856 0.1891829 0.1891829
## 130     KMT2B   290   727     5     9    54 1.024727 0.2531626 0.2531626
## 46       CHD4   264   818    17    17    82 1.081028 0.3900096 0.3900096
## 220      TERT   133   165     4     1     7 0.603562 0.2661251 0.2661251
## 77      FBXW7   104   354     5     2    45 1.266317 0.2019979 0.2019979
## 142     MED12   479  1186    48    27    54 1.001640 0.5461710 0.5461710
## 174    PIK3CA   149   529    16     6    58 1.123530 0.3711461 0.3711461
## 75       FAT2   756  1940    59    12   228 1.053457 0.5784833 0.5784833
##       wind_cv   psubneg_cv pindneg_cv   qsubneg_cv qglobalneg_cv
## 204 0.4415757 3.458234e-10 0.09572849 8.265179e-08  1.988424e-07
## 130 0.5028886 2.941933e-09 0.13308674 3.515610e-07  1.060263e-06
## 46  0.7172078 3.512168e-08 0.31174330 2.798027e-06  1.686093e-05
## 220 0.2633792 1.107017e-06 0.03463777 4.409620e-05  4.141525e-05
## 77  1.0419969 9.159586e-07 0.60374602 4.409620e-05  3.394065e-04
## 142 0.4959998 1.011306e-06 0.12811636 4.409620e-05  1.044117e-04
## 174 0.9024992 4.762778e-06 0.48610213 1.626148e-04  1.104768e-03
## 75  0.7996655 1.170226e-05 0.38346750 3.496051e-04  1.784935e-03
```

We can also boost our power to detect negative selection using RHT on essential genes from CRISPR screens.

```
# RHT for negative selection one-sided tests using non-driver essential genes
ess_selcv = dndsout$sel_cv[which(dndsout$sel_cv$gene_name %in% essential_passengers),]
ess_selcv$qval_essrht = p.adjust(ess_selcv$psubneg_cv, method="BH")
print(ess_selcv[ess_selcv$qval_essrht<0.05,c(1:10,15,17,22,28,31)])
```

```
##     gene_name n_syn n_mis n_non n_spl n_ind   wmis_cv   wnon_cv   wspl_cv
## 46       CHD4   264   818    17    17    82 1.0810281 0.3900096 0.3900096
## 174    PIK3CA   149   529    16     6    58 1.1235300 0.3711461 0.3711461
## 38       CDK4    62   107     5     3    15 0.6672832 0.4291227 0.4291227
##       wind_cv   psubneg_cv pindneg_cv   qsubneg_cv qglobalneg_cv  qval_essrht
## 46  0.7172078 3.512168e-08  0.3117433 2.798027e-06  1.686093e-05 5.970685e-07
## 174 0.9024992 4.762778e-06  0.4861021 1.626148e-04  1.104768e-03 4.048361e-05
## 38  0.8548530 4.600650e-03  0.4710032 1.221728e-01  3.442974e-01 2.607035e-02
```

Plotting the negative selection results.

```
# Genes to plot
negsel_genes = unique(c(negsel1$gene_name, ess_selcv$gene_name[ess_selcv$qval_essrht<0.05])) # Union of significant and RHT-significant
negsel = dndsout$sel_cv[dndsout$sel_cv$gene_name %in% negsel_genes, ]
negsel = negsel[order(negsel$psubneg_cv), ]
genes2plot = negsel$gene_name
ci = geneci(dndsout, gene_list = negsel_genes)
rownames(ci) = ci$gene
ci = ci[genes2plot, ]

if (runman) { dev.new(width=6.5, height=6) }
par(mfrow=c(2,1), mar=c(3,5.5,2,2))

# a. Mutations observed
nmuts = as.matrix(negsel[,2:5])
rownames(nmuts) = negsel$gene_name
nmuts = nmuts[genes2plot,]
subs_per_gene = t(nmuts)
rownames(subs_per_gene) = c("Synonymous","Missense","Nonsense","Splice")
colvec = c("grey70","cadetblue","darkorchid4","darkorchid2")
pos = barplot(subs_per_gene, las=2, col=colvec, border=NA, ylim=c(0, 3000), ylab="Total mutations")
legend(x=0, y=max(rowSums(nmuts))*1.15, legend=rownames(subs_per_gene), fill=colvec, border=NA, box.col=NA, bg="transparent")
verticalbar = mean(pos[(0:1)+length(drivers)])
abline(v=verticalbar)

# b. dN/dS ratios from the dNdScv model
pos = barplot(t(ci[,2:3]), beside=T, las=2, col=c("cadetblue","darkorchid3"), border=NA, ylim=c(0,2), ylab="dN/dS ratios")
segments(x0=pos, y0=t(ci[,4:5]), y1=t(ci[,6:7]))
legend(x=0, y=2.1, legend=c("Missense","Nonsense"), fill=c("cadetblue","darkorchid3"), border=NA, box.col=NA, bg="transparent")
abline(h=1, col="grey")
```

```
if (runman) { dev.copy(pdf,"Genes_under_negative_selection.pdf",width=6.5,height=6); dev.off() }
```

## 2. Mutational epidemiology: regression models on mutation landscapes

To study the impact of major epidemiological variables and risk factors on the mutation landscape, including on mutation rates, signatures and drivers, we first use multivariate mixed-effect linear regression models.

To study how different epidemiological factors explain variation in the frequency of driver mutations, we want to focus on genes with a large enough signal of selection. We can use dN/dS ratios to estimate the fraction of mutations in a gene that are drivers. The code below implements two alternative strategies:

1. Option 1: Selecting genes with a minimum fraction of driver mutations, considering all non-synonymous mutations together.
2. Option 2: Using only mutation classes with a minimum driver probability.

```
# Approximate fraction and number of driver mutations per driver gene
d = dndsout$sel_cv[which(dndsout$sel_cv$gene_name %in% drivers),]
driv_fract = (d[,7:10]-1)/d[,7:10]
rownames(driv_fract) = d$gene_name
driv_fract[driv_fract<0] = 0
numdrivers = driv_fract * d[,3:6]

driv_option = 2

if (driv_option == 1) {
  
  # To perform the regressions we select genes with a considerable number of mutations (eg >400) and with a high-enough driver fraction (eg >=30%).
  driv_fract_all = rowSums(numdrivers)/rowSums(d[,3:6]) # Fraction of all non-synonymous mutations that are predicted to be drivers
  genes2test = names(which(rowSums(numdrivers)>=400 & driv_fract_all>=0.30))
  putativedrivmuts = mutations[which((mutations$gene %in% genes2test) & (mutations$impact!="Synonymous")), ] # Non-synonymous mutations in the selected driver genes

} else if (driv_option == 2) {

  min_driver_fraction = 0.8 # Minimum driver fraction to annotate a class of mutations as a putative driver
  min_numdrivers = 1000 # Minimum number of driver mutations estimated in the high-driver-probability classes
  
  num_putative_drivers = driv_fract * d[,3:6] * (driv_fract>=min_driver_fraction) # This only considers mutation classes with a minimum dN/dS value
  driv_classes = (driv_fract>=min_driver_fraction)[drivers,]
  colnames(driv_classes) = c("Missense","Nonsense","Essential_Splice","no-SNV")

  putativedrivmuts = NULL
  for (j in 1:nrow(driv_classes)) {
    putativedrivmuts = rbind(putativedrivmuts, mutations[which(mutations$gene==rownames(driv_classes)[j] & mutations$impact %in% colnames(driv_classes)[driv_classes[j,]]), ])
  }
  
  # Genes to test individually in the regression models
  genes2test = names(which(sort(rowSums(num_putative_drivers),decreasing = T)>=min_numdrivers))
}

drivperdonor = split(putativedrivmuts, f=putativedrivmuts$sampleID) # Separating mutations per donor to calculate the driver density per donor
genes2test_ref = genes2test

for (j in 1:length(genes2test)) {
  vec = sapply(drivperdonor, function(x) sum(x$cellfraction[x$gene==genes2test[j] & x$impact!="Synonymous"], na.rm=T))
  metadata[,genes2test[j]] = vec[metadata$pd]
}

# Saving the metadata table
write.table(metadata, file = new_metadata_file, row.names=F, col.names=T, sep="\t", quote=F)

print(driv_classes[rowSums(driv_classes)>0,]) # Showing the mutation types considered high-confidence drivers
```

```
##         Missense Nonsense Essential_Splice no-SNV
## TP53        TRUE     TRUE             TRUE   TRUE
## CHEK2       TRUE     TRUE             TRUE   TRUE
## NOTCH1      TRUE     TRUE             TRUE   TRUE
## NOTCH2     FALSE     TRUE             TRUE   TRUE
## ATM        FALSE     TRUE             TRUE  FALSE
## FAT1       FALSE     TRUE             TRUE   TRUE
## RHOA       FALSE     TRUE             TRUE  FALSE
## RAC1        TRUE    FALSE            FALSE  FALSE
## KDM5C      FALSE     TRUE             TRUE  FALSE
## TET2       FALSE     TRUE             TRUE  FALSE
## ASXL1      FALSE     TRUE             TRUE   TRUE
## AJUBA      FALSE     TRUE             TRUE   TRUE
## EPHA2      FALSE     TRUE             TRUE  FALSE
## ZFP36L2    FALSE     TRUE             TRUE   TRUE
## ARID2      FALSE     TRUE             TRUE  FALSE
## MGA        FALSE     TRUE             TRUE  FALSE
## ARID1A     FALSE     TRUE             TRUE   TRUE
## PPM1D      FALSE     TRUE             TRUE   TRUE
## BCORL1     FALSE     TRUE             TRUE   TRUE
## ZFP36L1    FALSE    FALSE            FALSE   TRUE
## CCND1      FALSE     TRUE             TRUE  FALSE
## HLA-B      FALSE     TRUE             TRUE   TRUE
## PAX9       FALSE    FALSE            FALSE   TRUE
```

We can also calculate an aggregate measure of the driver density per donor.

```
# All driver genes (in option 2 above, this will sum all the mutations considered as high driver probability across all driver genes)
vec = sapply(drivperdonor, function(x) sum(x$cellfraction[x$impact!="Synonymous"], na.rm=T))
metadata$alldrivers = vec[metadata$pd]
exclude_genes = c("NOTCH1")
vec = sapply(drivperdonor, function(x) sum(x$cellfraction[!(x$gene %in% exclude_genes) & x$impact!="Synonymous"], na.rm=T))
metadata$alldrivers_noNOTCH1 = vec[metadata$pd]
```

We then perform epidemiological regressions. To facilitate testing different models, we use the function below that takes as input a dataset, a list of outcome variables, predictors, interactions (optional), random effects (optional), and normalisation choices (none, z-score or INT). To account for the sibling structure in the dataset, we can use (1|twins) as a random effect in a mixed-effect regression model.

```
# regfun function
# zscore = T performs Z-score normalisation (little to no impact to results but can help with interaction terms)
# int = T performs INT scaling (avoids excessive influence of outliers and normality violations but loses linearity)
# w = weights for the regression (NULL or numeric vector)
regfun = function(regdata, outcomes, predictors, interactions, random_effects = NULL, zscore = F, int = F, w = NULL, vars2scale = NULL) {
  
  pvals = coeffs = numeric() # Initialising
  
  # Normalising (scaling) variables (all variables by default, unless a specific set is requested using vars2scale)
  if (is.null(vars2scale)) {
    vars2scale = unique(c(outcomes,predictors))
  }
  if (zscore==T | int==T) {
    for (j in 1:length(vars2scale)) {
      if (is.numeric(regdata[,vars2scale[j]])) {
        if (zscore==T) {
          regdata[,vars2scale[j]] = scale(regdata[,vars2scale[j]], center=TRUE, scale=TRUE) # Z-scores
        } else {
          regdata[,vars2scale[j]] = qnorm((rank(regdata[,vars2scale[j]])-0.5)/nrow(regdata)) # INT normalisation
        }
      }
    }
  }
  
  # Reorganising the interaction terms (variables need to appear in the order provided as independent predictors)
  if (length(interactions)>0) {
    interactions = sapply(strsplit(interactions, split=":"), function(x) if (sum(predictors %in% x)==2) { paste(predictors[predictors %in% x], collapse=":") } else { stop("Input interactions between two existing variables ") } )
    if (zscore==F & int==F) {
      warning("Normalisation of the variables is recommended when using interaction terms (consider using zscore=T or int=T)")
    }
  }
  
  # If there are no random effects, we use a glm and Wald p-values. If there are random effects, we use lmer and LRT p-values.
  if (length(random_effects)==0) {
    
    for (j in 1:length(outcomes)) {
      f = as.formula(paste(outcomes[j], paste(c(predictors, interactions), collapse=" + "), sep = " ~ "))
      if (is.null(w)) {
        model = glm(f, data = regdata)
      } else {
        model = glm(f, data = regdata, weights = w)
      }
      pvals = cbind(pvals, coefficients(summary(model))[-1,4])
      coeffs = cbind(coeffs, coefficients(summary(model))[-1,1])
    }
    
  } else {
    
    for (j in 1:length(outcomes)) {
      f = as.formula(paste(outcomes[j], paste(c(predictors, interactions, random_effects), collapse=" + "), sep = " ~ "))
      if (is.null(w)) {
        model = lme4::lmer(f, data = regdata, REML=F)
      } else {
        model = lme4::lmer(f, data = regdata, REML=F, weights = w)
      }
      lrts = drop1(model, test="Chisq", scope = c(predictors,interactions))
      pvals = cbind(pvals, lrts[c(predictors,interactions),4])
      coeffs = cbind(coeffs, summary(model)$coefficients[-1,1])
      message(sprintf("lmer on %s",outcomes[j]))
    }
    rownames(pvals) = c(predictors,interactions)
    if (!all(substr(rownames(coeffs),1,3)==substr(rownames(pvals),1,3))) { stop("The row order of the pval and coeff output matrices does not seem to match") }
  }
  
  colnames(pvals) = colnames(coeffs) = outcomes
  qvals = array(p.adjust(pvals, method="BH"), dim=dim(pvals), dimnames = dimnames(pvals))
  
  return(list(formula = f, pvals=pvals, coeffs=coeffs, qvals=qvals, model=model))
}
```

To facilitate the visualisation of the regression results, we use the heatmap plotting function below.

```
# Plotting function
regression_heatmap = function(pvals, qvals, coeffs, filename = "heatmap.pdf", maxval = 10, plotwidth = 6, plotheight = 10) {
  
  require("gplots")
  require("RColorBrewer")
  
  logp = -log10(pvals) # -log10 transformation
  logp[logp>maxval] = maxval
  logp[coeffs<0] = -logp[coeffs<0]
  
  # Cell labels based on significance
  cellnote = array("", dim=dim(logp))
  cellnote[pvals<0.05] = "."
  cellnote[qvals<0.05] = "*"
  cellnote[qvals<0.01] = "**"
  cellnote[qvals<0.001] = "***"
  cellnote[qvals<0.0001] = "****"

  my_palette = colorRampPalette(c("steelblue4","steelblue2","white","indianred2","indianred4"))(n = 101)
  col_breaks = seq(-maxval,maxval,length.out=length(my_palette)+1)
  
  if (runman) { dev.new(width=plotwidth, height=plotheight) }
  logp = t(logp) # rotating the input matrix to have outcome variables as rows and rounding the values to improve the visualisation of the colorbar in pdf
  cellnote = t(cellnote)           # rotating the input matrix to have outcome variables as rows
  
  heatmap.2(logp,
            cellnote = cellnote,  # text labels for the cells
            notecex = 0.8,        # font size
            main = "",            # heat map title
            asp = 1,              # square cells
            notecol="black",      # change font color of cell labels to black
            density.info="none",  # turns off density plot inside color legend
            trace="none",         # turns off trace lines inside the heat map
            margins=c(4,4),       # widens margins around plot
            col=my_palette,       # use on color palette defined earlier 
            Colv="NA",            # turn off column clustering
            Rowv="NA",            # turn off row clustering
            dendrogram="none",    # no dendrogram
            breaks=col_breaks,    # fixing my own breaks
            scale="none",         # do not scale the values provided
            keysize = 0.5,        # size of the key bar
            key.title = "",       # do not plot a title for the key bar
            key.xlab = "log10(pval) (sign reflects direction of effect)",
            lmat=rbind(c(2,4,3),c(0,1,0),c(0,0,0)),
            lwid=c(plotwidth*0.1,plotwidth*0.7,plotwidth*0.2),
            lhei=c(1.2,(plotheight-1.2)*0.8,(plotheight-1.2)*0.2),
            cexRow=1, cexCol=1)            
  if (runman) { dev.copy(pdf, file = filename, width=plotwidth, height=plotheight); dev.off() }
}
```

We can now test for associations between epidemiological factors and the mutational landscape using different regression models.

```
min_duplexcov = 200 # We will only include samples that meet this criterium
min_age = 0 # We will only include samples that meet this criterium
burdens2test = c("burden_subs_passengers", "sig_denovo_sigA", "sig_denovo_sigB", "burden_indels_passengers", "dnv_burden")
outcomes = c(burdens2test, "alldrivers", genes2test_ref)
```

### Regression models

In this section we run a series of multivariate regression models to identify associations between multiple variables (including major risk factors for oral cancer) and different aspects of the mutational landscape, including mutation rates, signatures and driver densities. In addition to the main models discussed in the main text, we run a series of additional models to test the robustness of the associations against removal of outliers, INT normalisation or weighted regressions.

GLM on major epidemiological factors.

```
predictors = c("AGE","SEX","pack_years","drink_years","T2DM","BMI","missingteeth","ipaq_score","cancer")
inds = which(metadata$duplex_cov>=min_duplexcov & metadata$AGE>=min_age & !(metadata$donor %in% other_excluded_samples_from_regressions) 
             & metadata$hpv==0 & metadata$chemo==0 & rowSums(is.na(metadata[,c(outcomes,predictors)]))==0) # Samples to be considered in the model

regout = regfun(regdata=metadata[inds,], outcomes=outcomes, predictors=predictors, interactions=NULL, random_effects = NULL)
regression_heatmap(pvals=regout$pvals, qvals=regout$qvals, coeffs=regout$coeffs, filename = "Epidemiological_regressions_glm.pdf", maxval = 10, plotwidth = 4, plotheight = 6.5)
```

LMER on major epidemiological factors. This is the main analysis shown in the main text of the paper.

```
regout = regfun(regdata=metadata[inds,], outcomes=outcomes, predictors=predictors, interactions=NULL, random_effects = "(1|twin)")
regression_heatmap(pvals=regout$pvals, qvals=regout$qvals, coeffs=regout$coeffs, filename = "Epidemiological_regressions_lmer.pdf", maxval = 10, plotwidth = 4, plotheight = 6.5)
```

```
# Printing the coefficients for the impact of major variables on SNV burden
model = regfun(regdata=metadata[inds,], outcomes="burden_subs_passengers", predictors=predictors, interactions=NULL, random_effects = "(1|twin)")$model
modelcoeff = coefficients(summary(model))[,1]*genome_length # Coefficients scaled by diploid genome length
modelci = confint(model)*genome_length
modelout = cbind(coeff=modelcoeff,modelci[names(modelcoeff),])
print(modelout)
```

```
##                    coeff       2.5 %      97.5 %
## (Intercept)  166.6560594  19.1501186 314.3416977
## AGE           15.2203767  13.8105061  16.6257557
## SEXM         -12.2602645 -59.5753086  35.0179245
## pack_years     5.3835282   3.9668335   6.8006229
## drink_years    0.7982263   0.5985911   0.9978084
## T2DM           5.3730392 -47.3536167  58.0977262
## BMI            1.0199105  -2.2618538   4.3014663
## missingteeth  47.9729464  22.6571156  73.2982628
## ipaq_score     1.3565981 -23.8188679  26.5322915
## cancer        13.0232249 -25.6043915  51.6454807
```

As we explain in the manuscript, the estimates above suggest that an additional year of life seems considerably more mutagenic than 1 pack-year and much more mutagenic than 1 drink-year. This is explained and caveated in Supplementary Note 7. The code below provides the confidence intervals cited in this section of the manuscript.

```
aux = coefficients(summary(model))[,1:2] * genome_length # Estimates and standard errors
fieller_method = function(coeff1, se1, coeff2, se2, conf.level = 0.95) {
  r = coeff1 / coeff2 # Ratio of both coefficients
  se_r = sqrt((se1^2 / coeff2^2) + ((coeff1^2 * se2^2) / coeff2^4))
  z_alpha = qt(1 - (1 - conf.level) / 2, df = 100)
  ci_r = c(r - z_alpha * se_r, r + z_alpha * se_r)
  return(c(ratio=r, cilow=ci_r[1], cihigh=ci_r[2]))
}
print(fieller_method(aux["AGE",1],aux["AGE",2],aux["pack_years",1],aux["pack_years",2]))
```

```
##    ratio    cilow   cihigh 
## 2.827212 2.030304 3.624120
```

```
print(fieller_method(aux["AGE",1],aux["AGE",2],aux["drink_years",1],aux["drink_years",2]))
```

```
##    ratio    cilow   cihigh 
## 19.06775 13.93041 24.20509
```

LMER on major epidemiological factors using INT normalisation of outcome and predictor variables to reduce the influence of outliers.

```
regout = regfun(regdata=metadata[inds,], outcomes=outcomes, predictors=predictors, interactions=NULL, random_effects = "(1|twin)", int = T, vars2scale = c("alldrivers", genes2test_ref))
regression_heatmap(pvals=regout$pvals, qvals=regout$qvals, coeffs=regout$coeffs, filename = "Epidemiological_regressions_lmer_INTnorm.pdf", maxval = 10, plotwidth = 4, plotheight = 6.5)
```

As an alternative approach to reduce the impact of outlier donors for driver frequency, we use LMER on major epidemiological factors excluding outliers for driver frequencies.

```
excl_outliers = NULL
vars = c("alldrivers", genes2test_ref)
for (j in 1:length(vars)) {
  #cutoff = mean(metadata[,vars[j]],na.rm=T) + sd(metadata[,vars[j]],na.rm=T)*3 # Outliers: mean + 3*SD
  cutoff = quantile(metadata[,vars[j]], 0.75, na.rm=T) + IQR(metadata[,vars[j]],na.rm=T)*3 # Outliers: Q3 + IQR*3
  excl_outliers = c(excl_outliers, which(metadata[,vars[j]]>cutoff))
}
inds_exclout = setdiff(inds,excl_outliers)
regout = regfun(regdata=metadata[inds_exclout,], outcomes=outcomes, predictors=predictors, interactions=NULL, random_effects = "(1|twin)")
regression_heatmap(pvals=regout$pvals, qvals=regout$qvals, coeffs=regout$coeffs, filename = "Epidemiological_regressions_lmer_excldriveroutliers.pdf", maxval = 10, plotwidth = 4, plotheight = 6.5)
```

LMER on major epidemiological factors using mean duplex coverage per donor as a weight in the regression.

```
w = metadata[inds,"duplex_cov"] / mean(metadata[inds,"duplex_cov"])
regout = regfun(regdata=metadata[inds,], outcomes=outcomes, predictors=predictors, interactions=NULL, random_effects = "(1|twin)", w = w)
regression_heatmap(pvals=regout$pvals, qvals=regout$qvals, coeffs=regout$coeffs, filename = "Epidemiological_regressions_lmer_dcweights.pdf", maxval = 10, plotwidth = 4, plotheight = 6.5)
```

The association between the number of missing teeth and mutation rates (particularly signature A) is interesting but needs to be interpreted with caution. Whereas this could be a causal relationship between oral health and somatic mutations (e.g. through inflammation, microbiota, etc), it could also be due to indirect effects. For example, oral health is associated with deprivation and smoking, and incomplete metadata or inaccurate smoking histories could lead to indirect associations being found in our analyses. The tests below highlight the correlation between smoking and alcohol consumption, as well as between smoking and missing teeth. This emphasises the risk of indirect associations between these variables.

- https://www.ons.gov.uk/peoplepopulationandcommunity/healthandsocialcare/drugusealcoholandsmoking/bulletins/deprivationandtheimpactonsmokingprevalenceenglandandwales/2017to2021
- https://www.ncbi.nlm.nih.gov/pmc/articles/PMC6414580/

```
kruskal.test(pack_years~missingteeth, data = metadata[inds,])
```

```
## 
##  Kruskal-Wallis rank sum test
## 
## data:  pack_years by missingteeth
## Kruskal-Wallis chi-squared = 25.177, df = 3, p-value = 1.418e-05
```

```
kruskal.test(drink_years~missingteeth, data = metadata[inds,])
```

```
## 
##  Kruskal-Wallis rank sum test
## 
## data:  drink_years by missingteeth
## Kruskal-Wallis chi-squared = 3.4524, df = 3, p-value = 0.327
```

```
cor.test(metadata$pack_years[inds], metadata$drink_years[inds], method="spearman")
```

```
## 
##  Spearman's rank correlation rho
## 
## data:  metadata$pack_years[inds] and metadata$drink_years[inds]
## S = 53783534, p-value = 6.667e-08
## alternative hypothesis: true rho is not equal to 0
## sample estimates:
##       rho 
## 0.1971539
```

In the analyses above, we included only some of the most important epidemiological variables, including age, sex, BMI and the main risk factors of oral cancer for which we have some information. Below, we add a set of common medications to the regression analysis to look at their potential associations with mutation rates and driver densities. This analysis should be treated with caution, however, as the available medication metadata is incomplete (self-reported) and we do not have reliably information on duration of treatment or dosages. Also, associations are likely to not be causal, reflecting associations with the diseases treated by these medications or other co-morbidities or correlated lifestyle factors or exposures.

```
exclude_meds = NULL # We can choose to exclude metformin from analysis as it is too co-linear with T2DM.
core_vars = c("AGE","SEX","pack_years","drink_years","BMI","missingteeth","ipaq_score","cancer")
ext_vars = names(meds2test)

# Restricting the regressions to variables with a minimum number of informative donors
min_num_donors = 25 
donor_counts = setNames(rep(Inf,length(ext_vars)), ext_vars)
for (j in 1:length(ext_vars)) {
  vec = table(metadata[inds,ext_vars[j]])
  if (length(vec)==2 & any(names(vec) %in% c("1","TRUE"))) {
    donor_counts[j] = sum(vec[c("1","TRUE")], na.rm=T) # Counting the number of "1" or "TRUE" donors for YES/NO variables
  } else if (length(vec)==1) {
    donor_counts[j] = 0
  } else if (length(vec)>2) {
    donor_counts[j] = sum(vec)
  }
}
informative_variables = setdiff(names(donor_counts[donor_counts>=min_num_donors]), exclude_meds)

# 1. Regression on extended medications (T prefix)
vars = informative_variables[which(substr(informative_variables,1,1)=="T")] # Medications
vars = c(core_vars,sort(vars)) # Ordering covariates for the regression model
inds_ext = intersect(inds, which(rowSums(is.na(metadata[,vars]))==0))

regout = regfun(regdata=metadata[inds_ext,], outcomes=outcomes, predictors=vars, interactions=NULL, random_effects = "(1|twin)")
regression_heatmap(pvals=regout$pvals, qvals=regout$qvals, coeffs=regout$coeffs, filename = "Epidemiological_regressions_lmer_medications.pdf", maxval = 10, plotwidth = 10, plotheight = 5.5)
```

To ensure that the associations with drivers are not caused by outlier donors, we repeat the medication analysis using INT normalisation. This comes at the cost of losing linear dose-responses, so we apply INT normalisation only to drivers.

```
# LMER on major epidemiological factors and an extended list of common medications
regout = regfun(regdata=metadata[inds_ext,], outcomes=outcomes, predictors=vars, interactions=NULL, random_effects = "(1|twin)", int = T, vars2scale = c("alldrivers", genes2test_ref))
regression_heatmap(pvals=regout$pvals, qvals=regout$qvals, coeffs=regout$coeffs, filename = "Epidemiological_regressions_lmer_medications_INTnorm.pdf", maxval = 10, plotwidth = 10, plotheight = 5.5)
```

Alternatively, we exclude outlier donors from the regression.

```
inds_ext_out = setdiff(inds_ext,excl_outliers)
regout = regfun(regdata=metadata[inds_ext_out,], outcomes=outcomes, predictors=vars, interactions=NULL, random_effects = "(1|twin)")
regression_heatmap(pvals=regout$pvals, qvals=regout$qvals, coeffs=regout$coeffs, filename = "Epidemiological_regressions_lmer_medications_excldriveroutliers.pdf", maxval = 10, plotwidth = 10, plotheight = 5.5)
```

Beyond the extended medications, we can also test some additional metadata, such as exposure to heavy metals. This reveals a statistically very significant correlation between levels of lead and Signature B (SBS16). Whereas this is interesting, it could simply reflect the fact that alcohol consumption is known to be strongly correlated with lead levels in blood (a correlation that is also evident in our data) So, rather than a direct effect, this could reflect a residual effect of alcohol, not fully accounted for by correcting for drink\_years (possibly due to drink-years being an inaccurate estimate based on recent consumption).

```
# 2. Regression on heavy metals
vars = informative_variables[which(substr(informative_variables,1,1)=="E")] # Additional metadata
vars = c(core_vars,sort(vars)) # Ordering covariates for the regression model
inds_ext = intersect(inds, which(rowSums(is.na(metadata[,vars]))==0))
inds_ext_out = setdiff(inds_ext,excl_outliers)
regout = regfun(regdata=metadata[inds_ext_out,], outcomes=outcomes, predictors=vars, interactions=NULL, random_effects = "(1|twin)")
regression_heatmap(pvals=regout$pvals, qvals=regout$qvals, coeffs=regout$coeffs, filename = "Epidemiological_regressions_lmer_heavy_metals.pdf", maxval = 10, plotwidth = 6, plotheight = 5.5)
```

Here, to address a reviewer’s request and motivated by previous observations in clonal haematopoiesis, we explore whether age of menopause is associated with buccal mutation rates or clonal landscapes. No associations significant after multiple testing were found.

```
# 1. Observed age of menopause
vars = "A_menoage"
inds_ext = which(!is.na(metadata[,vars])) # Donors with information on the age of menopause
regout = regfun(regdata=metadata[inds_ext,], outcomes=outcomes, predictors=vars, interactions=NULL, random_effects = "(1|twin)")
print(regout$pvals["A_menoage",])
```

```
##   burden_subs_passengers          sig_denovo_sigA          sig_denovo_sigB 
##               0.69220868               0.80553010               0.72432464 
## burden_indels_passengers               dnv_burden               alldrivers 
##               0.75985606               0.08729599               0.61078953 
##                   NOTCH1                     TP53                     FAT1 
##               0.65123672               0.09751635               0.54882495 
##                    CHEK2                    PPM1D                    ASXL1 
##               0.71049476               0.56293080               0.10126380 
##                   NOTCH2                  ZFP36L2                     RAC1 
##               0.71387535               0.31252137               0.01486050 
##                   BCORL1 
##               0.51554899
```

```
#regout = regfun(regdata=metadata[inds_ext,], outcomes=c("burden_subs_passengers_blood","DNMT3A_blood","TET2_blood"), predictors=vars, interactions=NULL, random_effects = "(1|twin)")
#print(regout$pvals["A_menoage",])

# 2. PGS for age of menopause
vars = "PGS_ANM"
inds_ext = which(!is.na(metadata[,vars])) # Donors with information on the age of menopause
regout = regfun(regdata=metadata[inds_ext,], outcomes=outcomes, predictors=vars, interactions=NULL, random_effects = "(1|twin)")
print(regout$pvals["PGS_ANM",])
```

```
##   burden_subs_passengers          sig_denovo_sigA          sig_denovo_sigB 
##               0.46194326               0.44452809               0.78260930 
## burden_indels_passengers               dnv_burden               alldrivers 
##               0.45905056               0.89483654               0.11545307 
##                   NOTCH1                     TP53                     FAT1 
##               0.48411436               0.08749418               0.02349227 
##                    CHEK2                    PPM1D                    ASXL1 
##               0.49117821               0.03782985               0.54390428 
##                   NOTCH2                  ZFP36L2                     RAC1 
##               0.10686889               0.99406532               0.20524939 
##                   BCORL1 
##               0.70060593
```

Of particular interest is the overall effect of age, smoking and alcohol on the overall mutation burden. The code below prints out this regression model, which provides the coefficients needed to compare the impact on mutation burden of an additional year of life, an additional pack year, and an additional drink year.

```
predictors = c("AGE","SEX","pack_years","drink_years","T2DM","BMI","missingteeth","ipaq_score","cancer")
inds = which(metadata$duplex_cov>=min_duplexcov & metadata$AGE>=min_age & !(metadata$donor %in% other_excluded_samples_from_regressions) 
             & metadata$hpv==0 & metadata$chemo==0 & rowSums(is.na(metadata[,c(outcomes,predictors)]))==0) # Samples to be considered in the model
regout = regfun(regdata=metadata[inds,], outcomes="burden_subs_passengers", predictors=predictors, interactions=NULL, random_effects = "(1|twin)")
print(summary(regout$model))
```

```
## Linear mixed model fit by maximum likelihood  ['lmerMod']
## Formula: burden_subs_passengers ~ AGE + SEX + pack_years + drink_years +  
##     T2DM + BMI + missingteeth + ipaq_score + cancer + (1 | twin)
##    Data: regdata
## 
##      AIC      BIC   logLik deviance df.resid 
## -23116.6 -23061.3  11570.3 -23140.6      726 
## 
## Scaled residuals: 
##     Min      1Q  Median      3Q     Max 
## -3.3081 -0.5302 -0.0498  0.4250  8.1990 
## 
## Random effects:
##  Groups   Name        Variance  Std.Dev. 
##  twin     (Intercept) 3.488e-16 1.868e-08
##  Residual             1.086e-15 3.296e-08
## Number of obs: 738, groups:  twin, 544
## 
## Fixed effects:
##                Estimate Std. Error t value
## (Intercept)   2.939e-08  1.325e-08   2.218
## AGE           2.684e-09  1.262e-10  21.264
## SEXM         -2.162e-09  4.247e-09  -0.509
## pack_years    9.493e-10  1.273e-10   7.459
## drink_years   1.408e-10  1.793e-11   7.849
## T2DM          9.474e-10  4.736e-09   0.200
## BMI           1.798e-10  2.948e-10   0.610
## missingteeth  8.459e-09  2.274e-09   3.720
## ipaq_score    2.392e-10  2.262e-09   0.106
## cancer        2.296e-09  3.470e-09   0.662
## 
## Correlation of Fixed Effects:
##             (Intr) AGE    SEXM   pck_yr drnk_y T2DM   BMI    mssngt ipq_sc
## AGE         -0.625                                                        
## SEXM        -0.121  0.120                                                 
## pack_years  -0.035  0.004 -0.015                                          
## drink_years  0.033 -0.157 -0.177 -0.173                                   
## T2DM         0.189 -0.107 -0.080 -0.065  0.055                            
## BMI         -0.693  0.014  0.054 -0.003  0.033 -0.262                     
## missingteth  0.171 -0.260 -0.029 -0.196  0.080 -0.051 -0.087              
## ipaq_score  -0.542  0.056 -0.054  0.044 -0.076  0.014  0.269 -0.027       
## cancer      -0.051 -0.124  0.084  0.007 -0.019 -0.067  0.103 -0.049  0.050
```

Finally, we test as an additional a predictor a risk SNP for oral cancer (rs4767364) that showed evidence of a nominally significant association with SigB in the GWAS analysis. The SNP did not reach genome-wide significance to be considered a GWAS hit, but reached sufficient significance in the analysis of 68 a priori risk alleles for cancer or clonal haematopoiesis. Genome-wide genotypes for the GWAS analyses were available for only 590 of the buccal swab donors. However, genotyping of our targeted NanoSeq data recovered rs4767364 genotypes for 835 donors. Here we explore the associations of this SNP with the somatic mutation landscape.

```
risk_alleles = read.table(risk_alleles_file, header=1, sep="\t", stringsAsFactors = F)
rs4767364 = setNames(risk_alleles$rs4767364.12.112521448.G.A.ALDH2, risk_alleles$Donor)
metadata$rs4767364 = rs4767364[metadata$pd]
predictors = c("AGE","SEX","pack_years","drink_years","T2DM","BMI","missingteeth","ipaq_score","cancer","rs4767364")
inds = which(metadata$duplex_cov>=min_duplexcov & metadata$AGE>=min_age & !(metadata$donor %in% other_excluded_samples_from_regressions) 
             & metadata$hpv==0 & metadata$chemo==0 & rowSums(is.na(metadata[,c(outcomes,predictors)]))==0) # Samples to be considered in the model
regout = regfun(regdata=metadata[inds,], outcomes="sig_denovo_sigB", predictors=predictors, interactions=NULL, random_effects = "(1|twin)")
print(summary(regout$model))
```

```
## Linear mixed model fit by maximum likelihood  ['lmerMod']
## Formula: sig_denovo_sigB ~ AGE + SEX + pack_years + drink_years + T2DM +  
##     BMI + missingteeth + ipaq_score + cancer + rs4767364 + (1 |      twin)
##    Data: regdata
## 
##      AIC      BIC   logLik deviance df.resid 
## -20054.5 -19996.6  10040.2 -20080.5      618 
## 
## Scaled residuals: 
##     Min      1Q  Median      3Q     Max 
## -3.5721 -0.3580 -0.0888  0.2066 11.3383 
## 
## Random effects:
##  Groups   Name        Variance  Std.Dev. 
##  twin     (Intercept) 2.567e-16 1.602e-08
##  Residual             6.475e-16 2.545e-08
## Number of obs: 631, groups:  twin, 469
## 
## Fixed effects:
##                Estimate Std. Error t value
## (Intercept)   9.819e-09  1.265e-08   0.776
## AGE           1.436e-10  1.195e-10   1.201
## SEXM         -2.519e-10  4.160e-09  -0.061
## pack_years    4.694e-10  1.061e-10   4.424
## drink_years   1.378e-10  1.509e-11   9.131
## T2DM         -2.961e-09  3.983e-09  -0.743
## BMI           1.012e-10  2.629e-10   0.385
## missingteeth  1.984e-09  1.946e-09   1.019
## ipaq_score    1.223e-10  1.946e-09   0.063
## cancer       -7.250e-10  2.901e-09  -0.250
## rs4767364     5.032e-09  1.843e-09   2.730
## 
## Correlation of Fixed Effects:
##             (Intr) AGE    SEXM   pck_yr drnk_y T2DM   BMI    mssngt ipq_sc
## AGE         -0.684                                                        
## SEXM        -0.027  0.041                                                 
## pack_years  -0.048  0.024 -0.025                                          
## drink_years -0.016 -0.085 -0.181 -0.190                                   
## T2DM         0.211 -0.118 -0.036 -0.089  0.027                            
## BMI         -0.702  0.092 -0.004 -0.005  0.068 -0.297                     
## missingteth  0.176 -0.250  0.021 -0.180  0.040 -0.011 -0.104              
## ipaq_score  -0.508  0.062 -0.039  0.050 -0.092  0.011  0.284 -0.034       
## cancer      -0.047 -0.118  0.052  0.026 -0.015 -0.071  0.110 -0.053  0.049
## rs4767364   -0.118  0.018 -0.085  0.039 -0.012 -0.019  0.017  0.013  0.012
##             cancer
## AGE               
## SEXM              
## pack_years        
## drink_years       
## T2DM              
## BMI               
## missingteth       
## ipaq_score        
## cancer            
## rs4767364    0.001
```

### Mutagens vs selectogens

An approximately linear increase in driver density is expected with an increase in mutation rates. This assumption is valid under a diversity of models of clonal growth (see Supplementary Notes). For example, as long as most cells do not carry a driver mutation and competition between clones does not dominate, doubling the mutation rate is expected to double the fraction of cells with driver mutations. This is the case if driver mutations do not cause clonal expansions until the full complement of driver mutations is acquired (Armitage & Doll, 1954), but also if driver mutations lead to exponential, quadratic, or logistic clonal expansions, among other models. However, this assumption may not be valid under certain circumstances, such as increases in mutation rate occurring non-uniformly throughout life but concentrating early or late in life, as this will leave more or less time for clonal expansions. This is expected to be more relevant for continuous models of clonal growth (e.g. exponential or quadratic) than for constrained models (e.g. logistic). Given that the increase in driver density with age in the oral epithelium is approximately linear, with a small intercept, it is reasonable to approach the analysis of selectogenesis by normalising the driver density by the mutation burden, as we do below.

```
# Calculating the ratio between the driver fraction and the mutation burden
metadata$alldrivers_burdencorr = metadata$alldrivers / metadata$burden_subs_exons_passengers_observed
for (j in 1:length(genes2test)) {
  metadata[,sprintf("%s_burdencorr",genes2test[j])] = metadata[,genes2test[j]] / metadata$burden_subs_exons_passengers_observed
}
## Regressions
genes2test_corr = paste(genes2test, "_burdencorr", sep="")
regout = regfun(regdata=metadata[inds,], outcomes=c("alldrivers_burdencorr", genes2test_corr), predictors=predictors, interactions=NULL, random_effects = "(1|twin)") # No normalisation
regression_heatmap(pvals=regout$pvals, qvals=regout$qvals, coeffs=regout$coeffs, filename = "Epidemiological_regressions_lmer_driverperburden.pdf", maxval = 10, plotwidth = 4, plotheight = 4.5)
```

We then explore the impact of INT normalisation to avoid the risk of some associations being driven by outliers.

```
regout = regfun(regdata=metadata[inds,], outcomes=c("alldrivers_burdencorr", genes2test_corr), predictors=predictors, interactions=NULL, random_effects = "(1|twin)", int = T, vars2scale = c("alldrivers_burdencorr", genes2test_corr))
# Alternatively, the line below avoids outlier effects by excluding outliers from the regression
#regout = regfun(regdata=metadata[inds_exclout,], outcomes=c("alldrivers_burdencorr", genes2test_corr), predictors=predictors, interactions=NULL, random_effects = "(1|twin)", int = T, vars2scale = c("alldrivers_burdencorr", genes2test_corr))
regression_heatmap(pvals=regout$pvals, qvals=regout$qvals, coeffs=regout$coeffs, filename = "Epidemiological_regressions_lmer_driverperburden_INTnorm.pdf", maxval = 10, plotwidth = 4, plotheight = 4.5)
```

As an alternative approach to dividing driver density by mutation burden, we can include mutation burden as a covariate. This has some advantages (e.g. accounting for an intercept to consider a higher mutation burden before birth) and some disadvantages (e.g. possible risk of overcorrection), but the results are reassuringly similar. INT normalisation is not applied here to retain the linearity of the burden correction.

```
regout = regfun(regdata=metadata[inds,], outcomes=c("alldrivers", genes2test), predictors=c("burden_subs_exons_passengers_observed",predictors), interactions=NULL, random_effects = "(1|twin)")
regression_heatmap(pvals=regout$pvals, qvals=regout$qvals, coeffs=regout$coeffs, filename = "Epidemiological_regressions_lmer_burden_regressedout.pdf", maxval = 10, plotwidth = 4, plotheight = 4.5)
```

As a complementary way to study selectogenic effects, we can use dNdScv to estimate the neutral mutation rate of each gene in each donor. We note that this alternative analysis yields qualitatively similar results to using mean exonic burden across genes as a correction factor. However, the rate estimates per gene per donor are too noisy to be suitable correction factors in the current dataset as they rely on a low number of synonymous mutations per donor (fewer than 20 synonymous mutations were detected in ~37% of donors), resulting in a considerable loss of power. Although we do not recommend to use the code below in the current dataset, this is included here for completion and we anticipate the using dN/dS-like ratios as outcome variables should be possible in future studies with either larger gene panels or higher duplex coverage per gene.

```
if (file.exists("Estimated_nonsynrate_pergene_persample_dndscv.tsv")) {
  
  nsrate_persample = read.table("Estimated_nonsynrate_pergene_persample_dndscv.tsv", stringsAsFactors = F)

} else { # If the rate file does not exist, we run dndscv on every donor to generate it

  gene2dc_persample = read.table(dcpergene_file, header=1, sep="\t", stringsAsFactors=F) # Duplex coverage per sample
  mspl = split(mutations, f=mutations$sampleID)
  nsrate_persample = array(NA, dim = c(length(mspl),length(targetgenes)), dimnames = list(names(mspl),sort(targetgenes)))
  for (j in 1:length(mspl)) {
    try({
      g2dc = setNames(gene2dc_persample[,names(mspl)[j]],rownames(gene2dc_persample)) # Duplex coverage for the target genes for sample j 
      g2dc = pmax(g2dc,1e-6) # Using a minimum bound to be able to run dndscv
      d = dndscv(mspl[[j]], gene_list = targetgenes, max_muts_per_gene_per_sample = Inf, max_coding_muts_per_sample = Inf, constrain_wnon_wspl = T, mingenecovs = 500, dc = g2dc/mean(g2dc), kc = newkc, outmats = T, onesided = T, cv = dndscovs, refdb = RefCDS) # Without covariates
      nsrate_persample[j,] = setNames(rowSums(d$genemuts[,c("exp_mis","exp_non","exp_spl")]), d$genemuts$gene_name)[colnames(nsrate_persample)]
    }, silent = TRUE)
    if (round(j/10)==(j/10)) { message(sprintf('    %0.3g%% ...', round(j/length(mspl),2)*100)) }
  }
  write.table(nsrate_persample, file = "Estimated_nonsynrate_pergene_persample_dndscv.tsv", row.names=T, col.names=T, sep="\t", quote=F)
}

# Adding these background rate estimates to the metadata object
for (j in 1:length(genes2test)) {
  metadata[,sprintf("%s_burdencorrdnds",genes2test[j])] = metadata[,genes2test[j]] / nsrate_persample[metadata$pd,genes2test[j]]
}

# Regression
genes2test_corrdnds = paste(genes2test, "_burdencorrdnds", sep="")
regout = regfun(regdata=metadata[inds,], outcomes=genes2test_corrdnds, predictors=predictors, interactions=NULL, random_effects = "(1|twin)", int = T, vars2scale = genes2test_corrdnds)
# Alternatively, the line below avoids outlier effects by excluding outliers from the regression
#regout = regfun(regdata=metadata[inds_exclout,], outcomes=c("alldrivers_burdencorrdnds", genes2test_corrdnds), predictors=predictors, interactions=NULL, random_effects = "(1|twin)")
regression_heatmap(pvals=regout$pvals, qvals=regout$qvals, coeffs=regout$coeffs, filename = "Epidemiological_regressions_lmer_driverperdNdSburden_INTnorm.pdf", maxval = 10, plotwidth = 4, plotheight = 4.5)
```

As a complementary way to quantify whether some risk factors, and specifically smoking, may act as selectogens/promoters (ie. altering selection pressure and clonal expansions), we compare dN/dS ratios for NOTCH1 between smokers and non-smokers. We restrict the analysis to donors >=50 years old to minimise inter-group age differences.

```
# Groups and genes to plot
#donorgroups = list("0" = metadata$pd[which(metadata$pack_years==0 & metadata$AGE>=50)],
#                   "1-20" = metadata$pd[which(metadata$pack_years>=1 & metadata$pack_years<20 & metadata$AGE>=50)],
#                   "20-40" = metadata$pd[which(metadata$pack_years>=20 & metadata$pack_years<40 & metadata$AGE>=50)],
#                   "40+" = metadata$pd[which(metadata$pack_years>=40 & metadata$AGE>=50)]); f = "Pack years"

donorgroups = list("0" = metadata$pd[which(metadata$pack_years==0 & metadata$AGE>=50)],
                   "1-40" = metadata$pd[which(metadata$pack_years>=1 & metadata$pack_years<40 & metadata$AGE>=50)],
                   "40+" = metadata$pd[which(metadata$pack_years>=40 & metadata$AGE>=50)]); f = "Pack years"

donorgroups_dnds = NULL
donorgroups_burden = array(NA, dim=c(length(donorgroups),3), dimnames = list(names(donorgroups),c("mean","cilow","cihigh")))

# Calculating the differences in burden and selection between the 3 groups
for (j in 1:length(donorgroups)) {

  # Burden
  x = metadata$burden_subs_passengers[metadata$pd %in% donorgroups[[j]]] * genome_length
  #x = metadata$burden_subs_exons_passengers_observed[metadata$pd %in% donorgroups[[j]]] * genome_length
  donorgroups_burden[j,] = c(mean(x), as.vector(t.test(x)$conf.int)) # mean and CI95%
  
  # dN/dS
  maux = mutations[mutations$sampleID %in% donorgroups[[j]], 1:5]
  dndsout_j = dndscv(mutations = maux, gene_list = targetgenes, max_muts_per_gene_per_sample = Inf, max_coding_muts_per_sample = Inf, constrain_wnon_wspl = T, mingenecovs = 0, dc = gene2dc/mean(gene2dc), kc = newkc, outmats = T, onesided = T, cv = dndscovs, refdb = RefCDS, maxcovs = 10, use_indel_sites = use_indel_sites)
  ci_j = geneci(dndsout_j, gene_list = c("TP53","NOTCH1","CHEK2"))
  ci_j$exposure = j
  donorgroups_dnds = rbind(donorgroups_dnds, ci_j)
}

# Plots
genes = c("NOTCH1","TP53")

if (runman) { dev.new(width=12, height=5) }
par(mfrow=c(1,length(genes)*2+1))

h = barplot(donorgroups_burden[,1], ylim=c(0,max(donorgroups_burden)*1.1), las=2, col="grey60", border=NA, ylab = "Mean burden per diploid genome", main = "SNV burden", xlab = f)
segments(x0=h, y0=donorgroups_burden[,2], y1=donorgroups_burden[,3])

for (j in 1:length(genes)) {
  dnds = donorgroups_dnds[donorgroups_dnds$gene==genes[j],]
  # wmis
  h = barplot(dnds[,"mis_mle"], ylim=c(0,max(dnds[,"mis_high"])), las=2, col="cadetblue", border=NA, ylab = "Missense dN/dS", main = genes[j], names.arg = names(donorgroups), xlab = f)
  segments(x0=h, y0=dnds[,"mis_low"], y1=dnds[,"mis_high"])
  # wtru
  h = barplot(dnds[,"tru_mle"], ylim=c(0,max(dnds[,"tru_high"])), las=2, col="darkorchid4", border=NA, ylab = "Truncating dN/dS", main = genes[j], names.arg = names(donorgroups), xlab = f)
  segments(x0=h, y0=dnds[,"tru_low"], y1=dnds[,"tru_high"])
}
```

```
if (runman) { dev.copy(pdf, file = "Selectogens_smoking_NOTCH1_dNdS.pdf", width=12, height=5); dev.off() }
```

Finally, the analysis below explores the potential of using regression models on dN/dS ratios. Specifically, here we explore negative binomial regression models using the observed number of non-synonymous SNVs per gene per sample as the outcome variable, and the neutrally-expected rates (from dndscv) as offset, which equates to performing a regression on dN/dS ratios per gene per sample. Below, we run a standard negative binomial regression (MASS::glm.nb), and a mixed-effect negative binomial regression (lme4::glmer.nb) to account for twin pairs. Overall, these analyses yield comparable results to the analyses above using 2\*sum(duplexVAFs) as the outcome variable, identifying a highly-significant increase in NOTCH1 dN/dS ratios as a function of pack years. The analyses also identify a weaker putatively-selectogenic association between smoking and TP53, although caution should be exercised when interpreted modest associations given the assumptions of the models and the number of tests performed.

```
# Adding the number of observed and neutrally-expected non-synonymous mutations per gene to the metadata object
for (j in 1:length(genes2test)) {
  n = table(mutations$sampleID[which(mutations$gene==genes2test[j] & mutations$impact %in% c("Missense","Nonsense","Essential_Splice"))])[metadata$pd]; n[is.na(n)] = 0 # Number of non-syn mutations per sample
  metadata[,sprintf("%s_obs",genes2test[j])] = as.numeric(n)
  metadata[,sprintf("%s_exp",genes2test[j])] = nsrate_persample[metadata$pd,genes2test[j]]
}

# Negative binomial regressions for NOTCH1 and TP53 to test for changes in selection (dN/dS ratios).
# Below we used both a glm.nb model and a mixed-effect regression model accounting for twins as random effects (glmer.nb)
# NOTCH1
inds = intersect(which(rowSums(is.na(metadata[,c("NOTCH1_obs","NOTCH1_exp","AGE","SEX","pack_years","drink_years","missingteeth","cancer")]))==0),
                 which(metadata$NOTCH1_exp>0))
model_glmnb = MASS::glm.nb(NOTCH1_obs ~ offset(log(NOTCH1_exp)) + AGE + SEX + pack_years + drink_years + missingteeth + cancer, data=metadata[inds,])
model_glmernb = lme4::glmer.nb(NOTCH1_obs ~ offset(log(NOTCH1_exp)) + AGE + SEX + pack_years + drink_years + missingteeth + cancer + (1|twin), data=metadata[inds,])
print(summary(model_glmnb))
```

```
## 
## Call:
## MASS::glm.nb(formula = NOTCH1_obs ~ offset(log(NOTCH1_exp)) + 
##     AGE + SEX + pack_years + drink_years + missingteeth + cancer, 
##     data = metadata[inds, ], init.theta = 6.289758877, link = log)
## 
## Coefficients:
##                Estimate Std. Error z value Pr(>|z|)    
## (Intercept)   1.324e+00  9.982e-02  13.269  < 2e-16 ***
## AGE           2.120e-02  1.486e-03  14.271  < 2e-16 ***
## SEXM          3.446e-02  4.464e-02   0.772    0.440    
## pack_years    7.046e-03  1.408e-03   5.004 5.62e-07 ***
## drink_years   5.905e-05  1.925e-04   0.307    0.759    
## missingteeth  8.222e-03  2.588e-02   0.318    0.751    
## cancer       -2.277e-02  4.093e-02  -0.556    0.578    
## ---
## Signif. codes:  0 '***' 0.001 '**' 0.01 '*' 0.05 '.' 0.1 ' ' 1
## 
## (Dispersion parameter for Negative Binomial(6.2898) family taken to be 1)
## 
##     Null deviance: 1317.4  on 948  degrees of freedom
## Residual deviance: 1050.3  on 942  degrees of freedom
## AIC: 5833.1
## 
## Number of Fisher Scoring iterations: 1
## 
## 
##               Theta:  6.290 
##           Std. Err.:  0.468 
## 
##  2 x log-likelihood:  -5817.064
```

```
print(summary(model_glmernb))
```

```
## Generalized linear mixed model fit by maximum likelihood (Laplace
##   Approximation) [glmerMod]
##  Family: Negative Binomial(463.4152)  ( log )
## Formula: NOTCH1_obs ~ offset(log(NOTCH1_exp)) + AGE + SEX + pack_years +  
##     drink_years + missingteeth + cancer + (1 | twin)
##    Data: metadata[inds, ]
## 
##      AIC      BIC   logLik deviance df.resid 
##   5877.5   5921.2  -2929.8   5859.5      940 
## 
## Scaled residuals: 
##     Min      1Q  Median      3Q     Max 
## -2.4168 -0.6128 -0.0351  0.4744  5.6790 
## 
## Random effects:
##  Groups Name        Variance Std.Dev.
##  twin   (Intercept) 0.1361   0.3689  
## Number of obs: 949, groups:  twin, 664
## 
## Fixed effects:
##                Estimate Std. Error z value Pr(>|z|)    
## (Intercept)   1.2968951  0.1074823  12.066  < 2e-16 ***
## AGE           0.0203319  0.0015741  12.916  < 2e-16 ***
## SEXM          0.0157488  0.0467476   0.337    0.736    
## pack_years    0.0065782  0.0012528   5.251 1.52e-07 ***
## drink_years   0.0002403  0.0001699   1.414    0.157    
## missingteeth  0.0011830  0.0225767   0.052    0.958    
## cancer       -0.0258485  0.0313041  -0.826    0.409    
## ---
## Signif. codes:  0 '***' 0.001 '**' 0.01 '*' 0.05 '.' 0.1 ' ' 1
## 
## Correlation of Fixed Effects:
##             (Intr) AGE    SEXM   pck_yr drnk_y mssngt
## AGE         -0.971                                   
## SEXM        -0.193  0.126                            
## pack_years  -0.042  0.006 -0.007                     
## drink_years -0.001 -0.089 -0.155 -0.113              
## missingteth  0.143 -0.219 -0.006 -0.197  0.046       
## cancer       0.033 -0.099  0.067 -0.036 -0.009 -0.021
## optimizer (Nelder_Mead) convergence code: 0 (OK)
## Model failed to converge with max|grad| = 0.0051071 (tol = 0.002, component 1)
## Model is nearly unidentifiable: very large eigenvalue
##  - Rescale variables?
```

```
# TP53
inds = intersect(which(rowSums(is.na(metadata[,c("TP53_obs","TP53_exp","AGE","SEX","pack_years","drink_years","missingteeth","cancer")]))==0),
                 which(metadata$TP53_exp>0))
model_glmnb = MASS::glm.nb(TP53_obs ~ offset(log(TP53_exp)) + AGE + SEX + pack_years + drink_years + missingteeth + cancer, data=metadata[inds,])
model_glmernb = lme4::glmer.nb(TP53_obs ~ offset(log(TP53_exp)) + AGE + SEX + pack_years + drink_years + missingteeth + cancer + (1|twin), data=metadata[inds,])
print(summary(model_glmnb))
```

```
## 
## Call:
## MASS::glm.nb(formula = TP53_obs ~ offset(log(TP53_exp)) + AGE + 
##     SEX + pack_years + drink_years + missingteeth + cancer, data = metadata[inds, 
##     ], init.theta = 4.09057047, link = log)
## 
## Coefficients:
##                Estimate Std. Error z value Pr(>|z|)    
## (Intercept)   2.6938403  0.1319198  20.420  < 2e-16 ***
## AGE           0.0154018  0.0019673   7.829 4.93e-15 ***
## SEXM         -0.0207269  0.0597691  -0.347   0.7288    
## pack_years    0.0037376  0.0018657   2.003   0.0451 *  
## drink_years   0.0002008  0.0002532   0.793   0.4277    
## missingteeth  0.0202415  0.0341023   0.594   0.5528    
## cancer        0.0752082  0.0536667   1.401   0.1611    
## ---
## Signif. codes:  0 '***' 0.001 '**' 0.01 '*' 0.05 '.' 0.1 ' ' 1
## 
## (Dispersion parameter for Negative Binomial(4.0906) family taken to be 1)
## 
##     Null deviance: 1139.3  on 948  degrees of freedom
## Residual deviance: 1052.8  on 942  degrees of freedom
## AIC: 4787.8
## 
## Number of Fisher Scoring iterations: 1
## 
## 
##               Theta:  4.091 
##           Std. Err.:  0.344 
## 
##  2 x log-likelihood:  -4771.752
```

```
print(summary(model_glmernb))
```

```
## Generalized linear mixed model fit by maximum likelihood (Laplace
##   Approximation) [glmerMod]
##  Family: Negative Binomial(147.617)  ( log )
## Formula: 
## TP53_obs ~ offset(log(TP53_exp)) + AGE + SEX + pack_years + drink_years +  
##     missingteeth + cancer + (1 | twin)
##    Data: metadata[inds, ]
## 
##      AIC      BIC   logLik deviance df.resid 
##   4809.7   4853.4  -2395.9   4791.7      940 
## 
## Scaled residuals: 
##     Min      1Q  Median      3Q     Max 
## -2.4960 -0.6338 -0.0545  0.5076  5.8573 
## 
## Random effects:
##  Groups Name        Variance Std.Dev.
##  twin   (Intercept) 0.206    0.4539  
## Number of obs: 949, groups:  twin, 664
## 
## Fixed effects:
##                Estimate Std. Error z value Pr(>|z|)    
## (Intercept)   2.5998613  0.1432223  18.153  < 2e-16 ***
## AGE           0.0150182  0.0021045   7.136 9.59e-13 ***
## SEXM         -0.0314013  0.0631109  -0.498   0.6188    
## pack_years    0.0038345  0.0017572   2.182   0.0291 *  
## drink_years   0.0002024  0.0002277   0.889   0.3741    
## missingteeth  0.0163868  0.0309302   0.530   0.5963    
## cancer        0.1006707  0.0442334   2.276   0.0229 *  
## ---
## Signif. codes:  0 '***' 0.001 '**' 0.01 '*' 0.05 '.' 0.1 ' ' 1
## 
## Correlation of Fixed Effects:
##             (Intr) AGE    SEXM   pck_yr drnk_y mssngt
## AGE         -0.971                                   
## SEXM        -0.173  0.107                            
## pack_years  -0.041  0.003 -0.002                     
## drink_years  0.005 -0.095 -0.158 -0.115              
## missingteth  0.154 -0.228 -0.002 -0.200  0.031       
## cancer       0.025 -0.103  0.061 -0.016  0.025 -0.031
## optimizer (Nelder_Mead) convergence code: 0 (OK)
## Model is nearly unidentifiable: very large eigenvalue
##  - Rescale variables?
```

```
# CHEK2
inds = intersect(which(rowSums(is.na(metadata[,c("CHEK2_obs","CHEK2_exp","AGE","SEX","pack_years","drink_years","missingteeth","cancer")]))==0),
                 which(metadata$CHEK2_exp>0))
model_glmnb = MASS::glm.nb(CHEK2_obs ~ offset(log(CHEK2_exp)) + AGE + SEX + pack_years + drink_years + missingteeth + cancer, data=metadata[inds,])
model_glmernb = lme4::glmer.nb(CHEK2_obs ~ offset(log(CHEK2_exp)) + AGE + SEX + pack_years + drink_years + missingteeth + cancer + (1|twin), data=metadata[inds,])
print(summary(model_glmnb))
```

```
## 
## Call:
## MASS::glm.nb(formula = CHEK2_obs ~ offset(log(CHEK2_exp)) + AGE + 
##     SEX + pack_years + drink_years + missingteeth + cancer, data = metadata[inds, 
##     ], init.theta = 5.205140665, link = log)
## 
## Coefficients:
##               Estimate Std. Error z value Pr(>|z|)    
## (Intercept)  1.644e+00  1.760e-01   9.338  < 2e-16 ***
## AGE          1.438e-02  2.586e-03   5.560 2.69e-08 ***
## SEXM         2.896e-03  7.539e-02   0.038   0.9694    
## pack_years   3.840e-03  2.212e-03   1.736   0.0825 .  
## drink_years  6.849e-05  3.029e-04   0.226   0.8211    
## missingteeth 8.412e-02  4.057e-02   2.074   0.0381 *  
## cancer       5.618e-02  6.576e-02   0.854   0.3930    
## ---
## Signif. codes:  0 '***' 0.001 '**' 0.01 '*' 0.05 '.' 0.1 ' ' 1
## 
## (Dispersion parameter for Negative Binomial(5.2051) family taken to be 1)
## 
##     Null deviance: 1098.2  on 948  degrees of freedom
## Residual deviance: 1041.6  on 942  degrees of freedom
## AIC: 3273.1
## 
## Number of Fisher Scoring iterations: 1
## 
## 
##               Theta:  5.205 
##           Std. Err.:  0.836 
## 
##  2 x log-likelihood:  -3257.132
```

```
print(summary(model_glmernb))
```

```
## Generalized linear mixed model fit by maximum likelihood (Laplace
##   Approximation) [glmerMod]
##  Family: Negative Binomial(76.9808)  ( log )
## Formula: CHEK2_obs ~ offset(log(CHEK2_exp)) + AGE + SEX + pack_years +  
##     drink_years + missingteeth + cancer + (1 | twin)
##    Data: metadata[inds, ]
## 
##      AIC      BIC   logLik deviance df.resid 
##   3284.9   3328.5  -1633.4   3266.9      940 
## 
## Scaled residuals: 
##     Min      1Q  Median      3Q     Max 
## -2.0200 -0.7043 -0.1909  0.5813  4.1832 
## 
## Random effects:
##  Groups Name        Variance Std.Dev.
##  twin   (Intercept) 0.1392   0.3731  
## Number of obs: 949, groups:  twin, 664
## 
## Fixed effects:
##               Estimate Std. Error z value Pr(>|z|)    
## (Intercept)  1.572e+00  1.819e-01   8.642  < 2e-16 ***
## AGE          1.451e-02  2.646e-03   5.483 4.19e-08 ***
## SEXM         1.357e-02  7.627e-02   0.178   0.8588    
## pack_years   3.369e-03  2.171e-03   1.552   0.1207    
## drink_years  9.518e-05  2.873e-04   0.331   0.7404    
## missingteeth 6.737e-02  3.959e-02   1.701   0.0889 .  
## cancer       3.358e-02  6.178e-02   0.544   0.5868    
## ---
## Signif. codes:  0 '***' 0.001 '**' 0.01 '*' 0.05 '.' 0.1 ' ' 1
## 
## Correlation of Fixed Effects:
##             (Intr) AGE    SEXM   pck_yr drnk_y mssngt
## AGE         -0.971                                   
## SEXM        -0.168  0.110                            
## pack_years  -0.053  0.015  0.005                     
## drink_years -0.009 -0.084 -0.173 -0.108              
## missingteth  0.164 -0.242 -0.011 -0.224  0.036       
## cancer       0.047 -0.126  0.061 -0.006  0.010 -0.039
## optimizer (Nelder_Mead) convergence code: 0 (OK)
## Model failed to converge with max|grad| = 0.00248642 (tol = 0.002, component 1)
## Model is nearly unidentifiable: very large eigenvalue
##  - Rescale variables?
```

### Some additional regressions

Additional regression analyses on specific mutations within some driver genes:

1. Hotspot mutations in PIK3CA: relevant test given Hems et al 2024 (PMID:39169259) and given the general background of negative selection in PIK3CA confounding a gene-wide analysis.

```
# 1. PIK3CA hotspots
data("knownhotspots_hg19", package="dndscv")
pik3ca_known_hotspots = unlist(sapply(strsplit(known_hotspots, split=":"), function(x) { if (x[5]=="PIK3CA") { x[6] } }))
aux = split(mutations, f=mutations$sampleID) # Separating mutations per donor to calculate the driver density per donor
vec = sapply(aux, function(x) sum(x$cellfraction[x$gene=="PIK3CA" & x$aachange %in% pik3ca_known_hotspots], na.rm=T))
metadata$PIK3CA_hot = vec[metadata$pd]
predictors = c("AGE","SEX","pack_years","drink_years","T_metformin","BMI","missingteeth")
#predictors = c("AGE","SEX","pack_years","drink_years","T2D","BMI","missingteeth")
outcomes = c("PIK3CA_hot")
inds = which(metadata$duplex_cov>=min_duplexcov & metadata$AGE>=min_age & !(metadata$donor %in% other_excluded_samples_from_regressions) 
             & metadata$hpv==0 & metadata$chemo==0 & rowSums(is.na(metadata[,c(outcomes,predictors)]))==0) # Samples to be considered in the model
regout = regfun(regdata=metadata[inds,], outcomes=outcomes, predictors=predictors, interactions=NULL, random_effects = "(1|twin)")
outsummary = data.frame(predictor=rownames(regout$coeffs), coeffs=as.vector(regout$coeffs), pvals=as.vector(regout$pvals), qvals=as.vector(regout$qvals))
print(outsummary)
```

```
##      predictor        coeffs        pvals       qvals
## 1          AGE  1.323809e-05 0.0007460493 0.005222345
## 2         SEXM  2.350971e-05 0.8607288439 0.860728844
## 3   pack_years  1.288313e-06 0.7578964615 0.860728844
## 4  drink_years -3.073964e-07 0.5982566906 0.860728844
## 5  T_metformin -1.689995e-04 0.4178228223 0.860728844
## 6          BMI -6.592698e-06 0.4699205681 0.860728844
## 7 missingteeth  2.741662e-05 0.7125686300 0.860728844
```

2. Ratio of missense mutations in the DNA binding domain of TP53 vs truncating mutations in TP53.

```
# 2. TP53 missense vs truncating
tp53_DBDmis = sapply(aux, function(x) sum(x$cellfraction[which(x$gene=="TP53" & x$impact=="Missense" & as.numeric(substr(x$aachange,2,nchar(x$aachange)-1))>=95 & as.numeric(substr(x$aachange,2,nchar(x$aachange)-1))<=288)], na.rm=T))
tp53_trunc = sapply(aux, function(x) sum(x$cellfraction[which(x$gene=="TP53" & x$impact %in% c("Nonsense","Essential_Splice","no-SNV","Stop_loss"))], na.rm=T))
tp53_drivertype = tp53_trunc/(tp53_trunc+tp53_DBDmis)
metadata$tp53_drivertype = tp53_drivertype[metadata$pd]
outcomes = c("tp53_drivertype")
inds = which(metadata$duplex_cov>=min_duplexcov & metadata$AGE>=min_age & !(metadata$donor %in% other_excluded_samples_from_regressions) 
             & metadata$hpv==0 & metadata$chemo==0 & rowSums(is.na(metadata[,c(outcomes,predictors)]))==0) # Samples to be considered in the model
regout = regfun(regdata=metadata[inds,], outcomes=outcomes, predictors=predictors, interactions=NULL, random_effects = "(1|twin)")
outsummary = data.frame(predictor=rownames(regout$coeffs), coeffs=as.vector(regout$coeffs), pvals=as.vector(regout$pvals), qvals=as.vector(regout$qvals))
print(outsummary)
```

```
##      predictor        coeffs     pvals     qvals
## 1          AGE  1.239701e-04 0.8790571 0.9285545
## 2         SEXM -8.293264e-03 0.7564666 0.9285545
## 3   pack_years -7.173453e-05 0.9265452 0.9285545
## 4  drink_years  9.844300e-06 0.9285545 0.9285545
## 5  T_metformin -1.357627e-02 0.7321194 0.9285545
## 6          BMI  7.332267e-04 0.6744588 0.9285545
## 7 missingteeth  1.306782e-02 0.3472127 0.9285545
```

### Increase in driver fractions and dN/dS with age

The increase in driver density with age is an important measure, as it is a consequence of the changes in selection and clonal dynamics with age. For example, since mutations occur roughly linearly with age, if selection is age-independent and clonal growth is unconstrained (exponential), the increase in overall driver density would be predicted to be slightly faster than exponential. However, the pattern we observe in the buccal swabs is roughly linear o even sublinear. More careful analyses of the increase in driver density with age may inform about the mode of clonal growth (eg exponential vs quadratic vs logistic), the extent to which clonal competition slows down driver accumulation, etc. This is in turn relevant to understand the role of age (time) and of tissue “ageing” (potentially leading to changes in selection with age) in explaining the geometric increase in cancer risk with age.

```
genes2plot = c("NOTCH1","TP53","CHEK2","FAT1","PPM1D")

# Mutations by decade
ages = setNames(metadata$AGE, metadata$pd)[unique(mutations$sampleID)]
decades = setNames(cut(ages, breaks = seq(20,90,by=10), right = F), names(ages))
decades = decades[!is.na(decades)]
m = mutations
m$decade = decades[m$sampleID]
mutations_bydecade = split(m, f = m$decade)

# Initialising
drivage_stats = array(NA, dim=c(length(mutations_bydecade), length(genes2plot), 9), 
  dimnames = list(names(mutations_bydecade), genes2plot, c("mean", "low", "high", "largestclone", "largestclonelow", "largestclonehigh", "dndsmean", "dndslow", "dndshigh")))
drivage_dndsglobal = array(NA, dim=c(length(mutations_bydecade), 6), dimnames = list(names(mutations_bydecade), c("wmis","wmislow","wmishigh","wnon","wnonlow","wnonhigh")))
decade_meanage = array(NA, length(mutations_bydecade))

# Mean and CI95% using non-parametric bootstrapping
meanci = function(y, n=10000) {
    boot.samples = matrix(sample(y, size = length(y) * n, replace = TRUE), n, length(y))
    boot.statistics = apply(boot.samples, 1, mean)
    return(c(mean=mean(y), ci=quantile(boot.statistics, c(0.025,0.975))))
}

# Function maxorzero returns max(x) or 0 when x is empty
maxorzero = function(x, na.rm=T) {
  if (na.rm) { x = x[!is.na(x)] }
  if (length(x)==0) { 
    return(0)
  } else {
    return(max(x))
  }
}

for (d in 1:length(mutations_bydecade)) {

  decade_meanage[d] = mean(metadata$AGE[metadata$pd %in% unique(mutations_bydecade[[d]]$sampleID)])
  
  # Running dN/dS on individuals of that age
  dout = dndscv(mutations = mutations_bydecade[[d]], gene_list = targetgenes, max_muts_per_gene_per_sample = Inf, max_coding_muts_per_sample = Inf, constrain_wnon_wspl = T, mingenecovs = 0, dc = gene2dc/mean(gene2dc), kc = newkc, outmats = T, onesided = T, cv = dndscovs, refdb = RefCDS, maxcovs = 10, use_indel_sites = use_indel_sites)
  ci = geneci(dout, gene_list = genes2plot)
  drivage_dndsglobal[d, 1:3] = as.numeric(dout$globaldnds[1,2:4])
  drivage_dndsglobal[d, 4:6] = as.numeric(dout$globaldnds[2,2:4])
  
  # Calculating summary metrics on driver frequency: (1) mean estimated driver fraction, (2) mean size of the largest clone per donor.
  maux = split(mutations_bydecade[[d]], f = mutations_bydecade[[d]]$sampleID)
  for (g in 1:length(genes2plot)) {
    drivfrac_vec = sapply(maux, function(x) sum(x$cellfraction[x$gene==genes2plot[g] & x$impact!="Synonymous"], na.rm=T))
    drivage_stats[d, g, 1:3] = meanci(drivfrac_vec)
    largestclone_vec = sapply(maux, function(x) maxorzero(x$bam_adj_cellfraction[x$gene==genes2plot[g] & x$impact!="Synonymous"], na.rm=T))
    drivage_stats[d, g, 4:6] = meanci(largestclone_vec)
    drivage_stats[d, g, 7:9] = as.numeric(ci[ci$gene==genes2plot[g],c(3,5,7)]) # MLEs and CI95% for wtru for each gene
  }
}

# Barplots of the increase in driver frequency with age
if (runman) { dev.new(width=7, height=7) }
par(mfrow=c(2,2))

b = barplot(drivage_stats[,,1]*100, beside=T, las=2, ylab="Estimated % mutant cells", col = hcl.colors(length(mutations_bydecade), "Sunset"), ylim=c(0,max(drivage_stats[,,3]*100)))
segments(x0=b, y0=drivage_stats[,,2]*100, y1=drivage_stats[,,3]*100)
legend(x = mean(b)*1.4, y = max(drivage_stats[,,3]*100), legend = names(mutations_bydecade), col = hcl.colors(length(mutations_bydecade), "Sunset"), pch=15, box.col="white")

b = barplot(drivage_stats[,,7], beside=T, las=2, ylab="dN/dS truncating", col = hcl.colors(length(mutations_bydecade), "Sunset"), ylim=c(0,max(drivage_stats[,,9])))
segments(x0=b, y0=drivage_stats[,,8], y1=drivage_stats[,,9])

b = barplot(t(drivage_stats[,,1]*100), las=2, ylab="Estimated % mutant cells", col = hcl.colors(length(genes2plot), "Temps"))
legend(x = 0, y = 25, legend = genes2plot, col = hcl.colors(length(genes2plot), "Temp"), pch=15, box.col=NA)

b = barplot(t(drivage_dndsglobal[,c(1,4)]), beside=T, las=2, col=c("cadetblue","darkorchid"), border=NA, ylim=c(0,max(drivage_dndsglobal[,6])), ylab="Global dN/dS")
abline(h=1)
segments(x0=b, y0=t(drivage_dndsglobal[,c(2,5)]), y1=t(drivage_dndsglobal[,c(3,6)]))
legend(x = 1, y = max(drivage_dndsglobal[,6]), legend = c("Missense","Nonsense"), col = c("cadetblue","darkorchid"), pch=15, box.col=NA)
```

```
if (runman) { dev.copy(pdf, "Increase_driver_density_age.pdf", width=7, height=7); dev.off() }
```

Line plots of the increase in driver frequency with age, plotting each gene separately. The increase with age of the largest PPM1D clones is likely caused by PPM1D clones in blood, contaminating the buccal samples.

```
if (runman) { dev.new(width=length(genes2plot)*2, height=2.5) }
par(mfrow=c(1,length(genes2plot)))

for (j in 1:length(genes2plot)) {
  plot(x=decade_meanage, y=drivage_stats[,j,1], pch=19, col="lightslategrey", ylim=c(0,max(drivage_stats[,j,3])), main=genes2plot[j], las=1, xlab="Age",ylab="Estimated cell fraction", cex=0.8, xlim=c(20,90)) # Estimated driver fraction
  lines(x=decade_meanage, y=drivage_stats[,j,1], col="lightslategrey") # Connecting line
  segments(x0=decade_meanage, y0=drivage_stats[,j,2], y1=drivage_stats[,j,3], col="lightslategrey") # CI95%
  
  points(x=decade_meanage, y=drivage_stats[,j,4], pch=19, col="lightsalmon2") # Largest clone
  lines(x=decade_meanage, y=drivage_stats[,j,4], col="lightsalmon2") # Connecting line
  segments(x0=decade_meanage, y0=drivage_stats[,j,5], y1=drivage_stats[,j,6], col="lightsalmon2") # Largest clone CI95%
}
```

```
if (runman) { dev.copy(pdf, "Increase_driver_density_age_lineplot.pdf", width=length(genes2plot)*2, height=2.5); dev.off() }
```

### Plots of associations between risk factors and mutational/clonal landscapes

Plotting the increase in mutation burden as a function of pack years, regressing out the effect of other confounders.

```
inds = which(metadata$duplex_cov>=min_duplexcov & metadata$AGE>=min_age & !(metadata$donor %in% other_excluded_samples_from_regressions) 
             & metadata$hpv==0 & metadata$chemo==0 & rowSums(is.na(metadata[,c(outcomes,predictors)]))==0) # Samples to be considered in the model
vec = seq(0,50,by=5) # breaks of pack years
metadata$pack_years_bins = cut(metadata$pack_years, breaks = c(-1,vec), include.lowest = F)
model = glm(metadata[inds,"burden_subs_passengers"] ~ AGE + SEX + pack_years_bins + drink_years + T2DM + num_teeth, data = metadata[inds,])
model2 = glm(metadata[inds,"burden_subs_passengers"] ~ AGE + SEX + pack_years + drink_years + T2DM + num_teeth, data = metadata[inds,])

packbins = cbind(coeff=coefficients(model), confint.default(model))
packbins = packbins[substr(rownames(packbins),1,4)=="pack",]

if (runman) { dev.new(width=3.5, height=4) }
xmids = vec[-1]-(diff(vec)/2) # mid point of each pack year bracket
plot(xmids, packbins[,1], ylim=c(0,max(packbins)), xlim=c(0,max(vec)), las=1, xlab="Smoking (pack years)", ylab="Added burden (regressing out confounders)")
segments(x0=xmids, y0=packbins[,2], y1=packbins[,3])
abline(a=0, b=coefficients(model2)["pack_years"], col="grey")
```

```
if (runman) { dev.copy(pdf,"Epidemiology_burden_vs_pack_years.pdf",width=4.5,height=5); dev.off() }
```

Plotting the impact of smoking on Signature B.

```
model = glm(metadata[inds,"sig_denovo_sigB"] ~ AGE + SEX + pack_years_bins + drink_years + T2DM + num_teeth + total_consumption, data = metadata[inds,])
model2 = glm(metadata[inds,"sig_denovo_sigB"] ~ AGE + SEX + pack_years + drink_years + T2DM + num_teeth + total_consumption, data = metadata[inds,])

packbins = cbind(coeff=coefficients(model), confint.default(model))
packbins = packbins[substr(rownames(packbins),1,4)=="pack",]

if (runman) { dev.new(width=3.5, height=4) }
xmids = vec[-1]-(diff(vec)/2) # mid point of each pack year bracket
plot(xmids, packbins[,1], ylim=c(min(packbins),max(packbins)), xlim=c(0,max(vec)), las=1, xlab="Smoking (pack years)", ylab="Added sigB burden (regressing out confounders)")
segments(x0=xmids, y0=packbins[,2], y1=packbins[,3])
abline(a=0, b=coefficients(model2)["pack_years"], col="grey")
```

```
if (runman) { dev.copy(pdf,"Epidemiology_sigB_burden_vs_pack_years.pdf",width=4.5,height=5); dev.off() }
```

Plotting the increase in signature B burden as a function of drink years, regressing out the effect of other confounders.

```
#vec = c(seq(0,350,by=70),600) # breaks of pack years
vec = seq(0,350,by=70) # breaks of pack years
metadata$drink_years_bins = cut(metadata$drink_years, breaks = c(-1,vec), include.lowest = F)
model = glm(metadata[inds,"sig_denovo_sigB"] ~ AGE + SEX + drink_years_bins + pack_years + T2DM + num_teeth, data = metadata[inds,])
model2 = glm(metadata[inds,"sig_denovo_sigB"] ~ AGE + SEX + drink_years + pack_years + T2DM + num_teeth, data = metadata[inds,])

drinkbins = cbind(coeff=coefficients(model), confint.default(model))
drinkbins = drinkbins[substr(rownames(drinkbins),1,4)=="drin",]

if (runman) { dev.new(width=3.5, height=4) }
xmids = vec[-1]-(diff(vec)/2) # mid point of each pack year bracket
plot(xmids, drinkbins[,1], ylim=c(0,max(drinkbins)), xlim=c(0,max(vec)), las=1, xlab="Alcohol (drink years)", ylab="Added sigB burden (regressing out confounders)")
segments(x0=xmids, y0=drinkbins[,2], y1=drinkbins[,3])
abline(a=0, b=coefficients(model2)["drink_years"], col="grey")
```

```
if (runman) { dev.copy(pdf,"Epidemiology_sigB_burden_vs_drink_years.pdf",width=4.5,height=5); dev.off() }
```

Scatter plots of the increase in burden with age:

1. Age vs signature A.
2. Age vs signature B, colouring heavy drinkers.

```
if (runman) { dev.new(width=7.5, height=4) }
par(mfrow = c(1,2))

# Age vs signature A
min_duplexcov = 200
inds = which(metadata$duplex_cov>=min_duplexcov & !(metadata$donor %in% other_excluded_samples_from_regressions) & metadata$hpv==0 & metadata$chemo==0) 
plot(metadata$AGE[inds], metadata$sig_denovo_sigA[inds]*genome_length, cex=0.5, pch=16, las=1, col="grey20", xlab="Age", ylab="Signature A burden")
ct = cor.test(metadata$AGE[inds],metadata$sig_denovo_sigA[inds])

model = glm((sig_denovo_sigA*genome_length)~AGE, data=metadata[inds,])
abline(model, col="darkslategray4")
cimod = confint(model)
```

```
## Waiting for profiling to be done...
```

```
leg = sprintf("Intercept: %0.1f (%0.1f-%0.1f)\nSlope: %0.1f (%0.1f-%0.1f)\nR2: %0.2f, P=%0.2g",
              model$coefficients[1], cimod[1,1], cimod[1,2], model$coefficients[2], cimod[2,1], cimod[2,2],
              ct$estimate^2, ct$p.value)
text(x=min(metadata$AGE), y=max(metadata$sig_denovo_sigA[inds],na.rm=T)*genome_length, labels=leg, adj=c(0,1))

# Age vs signature B
min_duplexcov = 200
inds = which(metadata$duplex_cov>=min_duplexcov & !(metadata$donor %in% other_excluded_samples_from_regressions) & metadata$hpv==0 & metadata$chemo==0) 
plot(metadata$AGE[inds], metadata$sig_denovo_sigB[inds]*genome_length, cex=0.5, pch=16, las=1, col="grey20", xlab="Age", ylab="Signature B burden")
inds2 = intersect(inds, which(metadata$drink_years>=50)) # Moderate or heavy drinker
points(metadata$AGE[inds2], metadata$sig_denovo_sigB[inds2]*genome_length, cex=0.5, pch=16, col="darkorchid")
abline(lm(metadata$sig_denovo_sigB[inds]*genome_length~metadata$AGE[inds]), col="darkslategray4")
legend(x=20, y=max(metadata$sig_denovo_sigB[inds]*genome_length), legend = c("Drink years >= 50"), col = "darkorchid", box.col=NA, pch=16)
```

```
if (runman) { dev.copy(pdf,"Epidemiology_some_associations.pdf",width=7.5,height=4); dev.off() }
```

Scatter plots of the increase in SNVs with age using whole-genome NanoSeq (restriction enzyme protocol).

```
if (runman) { dev.new(width=4, height=4.5) }

# SNVs vs age
plot(burdensWGS$age[burdensWGS$inclusion_reason=="Age_Range"], burdensWGS$re_corrected_burden[burdensWGS$inclusion_reason=="Age_Range"]*genome_length, cex=0.8, pch=16, las=1, col="grey20", xlab="Age", ylab="SNV burden per cell", ylim=c(0,7500), xlim=c(0,90))
segments(x0=burdensWGS$age[burdensWGS$inclusion_reason=="Age_Range"], y0=burdensWGS$re_corrected_burden_lci[burdensWGS$inclusion_reason=="Age_Range"]*genome_length,
         y1=burdensWGS$re_corrected_burden_uci[burdensWGS$inclusion_reason=="Age_Range"]*genome_length, col="grey20")
points(burdensWGS$age[burdensWGS$inclusion_reason=="High_SigB"], burdensWGS$re_corrected_burden[burdensWGS$inclusion_reason=="High_SigB"]*genome_length, cex=0.8, pch=16, col="grey70")
segments(x0=burdensWGS$age[burdensWGS$inclusion_reason=="High_SigB"], y0=burdensWGS$re_corrected_burden_lci[burdensWGS$inclusion_reason=="High_SigB"]*genome_length,
         y1=burdensWGS$re_corrected_burden_uci[burdensWGS$inclusion_reason=="High_SigB"]*genome_length, col="grey70")
points(burdensWGS$age[burdensWGS$inclusion_reason=="CHOP"], burdensWGS$re_corrected_burden[burdensWGS$inclusion_reason=="CHOP"]*genome_length, cex=0.8, pch=16, col="darkorange")
segments(x0=burdensWGS$age[burdensWGS$inclusion_reason=="CHOP"], y0=burdensWGS$re_corrected_burden_lci[burdensWGS$inclusion_reason=="CHOP"]*genome_length,
         y1=burdensWGS$re_corrected_burden_uci[burdensWGS$inclusion_reason=="CHOP"]*genome_length, col="darkorange")

ct = cor.test(burdensWGS$age[burdensWGS$inclusion_reason=="Age_Range"], burdensWGS$re_corrected_burden[burdensWGS$inclusion_reason=="Age_Range"]*genome_length)

model = glm((burdensWGS$re_corrected_burden[burdensWGS$inclusion_reason=="Age_Range"]*genome_length)~burdensWGS$age[burdensWGS$inclusion_reason=="Age_Range"])
abline(model, col="grey20", lty=2)
cimod = confint(model)
```

```
## Waiting for profiling to be done...
```

```
leg = sprintf("Intercept: %0.1f (%0.1f-%0.1f)\nSlope: %0.1f (%0.1f-%0.1f)\nR2: %0.2f, P=%0.2g",
              model$coefficients[1], cimod[1,1], cimod[1,2], model$coefficients[2], cimod[2,1], cimod[2,2],
              ct$estimate^2, ct$p.value)
text(x=1, y=5000, labels=leg, adj=c(0,1))
```

```
if (runman) { dev.copy(pdf,"Whole_genome_NanoSeq_burdens.pdf",width=4,height=4.5); dev.off() }
```

### Age and epidemiological associations for blood

Plotting the increase in blood mutation burden with age.

```
use_ci95 = T

if (runman) { dev.new(width=5, height=6) }
if (use_ci95==F) {
  plot(bb$AGE, bb$burden_subs_passengers*genome_length, las=1, xlab="Age", ylab="Blood passenger burden (SNVs/diploid cell)", pch=16, ylim=c(0,max(bb$burden_subs_passengers, na.rm=T)*genome_length))
} else {
  x = bb$AGE + runif(nrow(bb),-0.5,0.5)
  #plot(x, bb$burden_subs_passengers*genome_length, las=1, xlab="Age", ylab="Blood passenger burden (SNVs/diploid cell)", pch=16, cex=0.6, ylim=c(0,max(bb$burden_boot_repl_uci[!is.na(bb$burden_subs_passengers)])*genome_length))
  plot(x, bb$burden_subs_passengers*genome_length, las=1, xlab="Age", ylab="Blood passenger burden (SNVs/diploid cell)", pch=16, cex=0.6, ylim=c(0,2600))
  segments(x0=x[!is.na(bb$burden_subs_passengers)], y0=bb$burden_boot_repl_lci[!is.na(bb$burden_subs_passengers)]*genome_length, y1=bb$burden_boot_repl_uci[!is.na(bb$burden_subs_passengers)]*genome_length, col="grey", lwd=0.5)
  points(x, bb$burden_subs_passengers*genome_length, las=1, pch=16)
}
model = lm(burden_subs_passengers*genome_length~AGE, data=bb[!is.na(bb$burden_subs_passengers), ])
abline(model)
cimod = confint(model)
leg = sprintf("Intercept: %0.2f (%0.2f-%0.2f)\nSlope: %0.2f (%0.2f-%0.2f)",
              model$coefficients[1],cimod[1,1],cimod[1,2], model$coefficients[2],cimod[2,1],cimod[2,2])
text(x=min(bb$AGE), y=max(bb$burden_subs_passengers,na.rm=T)*genome_length, labels=leg, adj=c(0,1))
```

```
if (runman) { dev.copy(pdf,"Blood_burden_vs_age.pdf",width=5,height=6); dev.off() }
```

Calculating the driver density of major blood drivers per donor from the blood data using the same approach used for buccal swabs.

```
# Mutation cell fractions assuming heterozygous mutations and correcting for copy number in sex chromosomes for males
males = metadata$pd[metadata$SEX=="M"]
bloodmuts$cellfraction = bloodmuts$duplex_vaf * 2
bloodmuts$cellfraction[bloodmuts$chr %in% c("X","Y") & bloodmuts$sampleID %in% males] = bloodmuts$duplex_vaf[bloodmuts$chr %in% c("X","Y") & bloodmuts$sampleID %in% males]

# Approximate fraction and number of driver mutations per driver gene
d = dndsout_blood$sel_cv[which(dndsout_blood$sel_cv$gene_name %in% drivers_blood),]
driv_fract = (d[,7:10]-1)/d[,7:10]
rownames(driv_fract) = d$gene_name
driv_fract[driv_fract<0] = 0
numdrivers = driv_fract * d[,3:6]

genes2test = c("DNMT3A","TET2","CHEK2","PPM1D") # For blood, we restrict the tests to these 4 main genes based on their frequency and prior associations
driv_option = 2

if (driv_option == 1) {
  
  driv_fract_all = rowSums(numdrivers)/rowSums(d[,3:6]) # Fraction of all non-synonymous mutations that are predicted to be drivers
  #genes2test = names(which(rowSums(numdrivers)>=100 & driv_fract_all>=0.30))
  putativedrivmuts_blood = bloodmuts[which((bloodmuts$gene %in% genes2test) & (bloodmuts$impact!="Synonymous")), ] # Non-synonymous mutations in the selected driver genes

} else if (driv_option == 2) {

  min_driver_fraction = 0.8 # Minimum driver fraction to annotate a class of mutations as a putative driver
  min_numdrivers = 50 # Minimum number of driver mutations estimated in the high-driver-probability classes
  
  num_putative_drivers = driv_fract * d[,3:6] * (driv_fract>=min_driver_fraction) # This only considers mutation classes with a minimum dN/dS value
  driv_classes = (driv_fract>=min_driver_fraction)[drivers_blood,]
  colnames(driv_classes) = c("Missense","Nonsense","Essential_Splice","no-SNV")

  putativedrivmuts_blood = NULL
  for (j in 1:nrow(driv_classes)) {
    putativedrivmuts_blood = rbind(putativedrivmuts_blood, bloodmuts[which(bloodmuts$gene==rownames(driv_classes)[j] & bloodmuts$impact %in% colnames(driv_classes)[driv_classes[j,]]), ])
  }
  # Genes to test individually in the regression models
  #genes2test = names(which(sort(rowSums(num_putative_drivers),decreasing = T)>=min_numdrivers))
}

genes2test_blood = genes2test # Saving this variable
drivperdonor = split(putativedrivmuts_blood, f=putativedrivmuts_blood$sampleID) # Separating mutations per donor to calculate the driver density per donor
blood_samples = unique(bloodmuts$sampleID)

for (j in 1:length(genes2test)) {
  vec = sapply(drivperdonor, function(x) sum(x$cellfraction[x$gene==genes2test[j] & x$impact!="Synonymous"], na.rm=T))
  vec = vec[blood_samples]; names(vec) = substr(blood_samples,1,7); vec[is.na(vec)] = 0 # Adding 0s to samples that were sequenced but did not have a mutation in these genes
  metadata[,sprintf("%s_blood",genes2test[j])] = vec[substr(metadata$pd,1,7)]
}

# All driver genes (in option 2 above, this will sum all the mutations considered as high driver probability across all driver genes)
vec = sapply(drivperdonor, function(x) sum(x$cellfraction[x$impact!="Synonymous"], na.rm=T))
vec = vec[blood_samples]; names(vec) = substr(blood_samples,1,7); vec[is.na(vec)] = 0 # Adding 0s to samples that were sequenced but did not have a mutation in these genes
metadata$alldrivers_blood = vec[substr(metadata$pd,1,7)]

# Saving the new metadata table
write.table(metadata, file = new_metadata_file, row.names=F, col.names=T, sep="\t", quote=F)

print(driv_classes[rowSums(driv_classes)>0,]) # Showing the mutation types considered high-confidence drivers
```

```
##        Missense Nonsense Essential_Splice no-SNV
## DNMT3A     TRUE     TRUE             TRUE   TRUE
## CHEK2      TRUE     TRUE             TRUE  FALSE
## TET2      FALSE     TRUE             TRUE   TRUE
## PPM1D     FALSE     TRUE             TRUE   TRUE
## ASXL1     FALSE     TRUE             TRUE  FALSE
## CDKN1B    FALSE     TRUE             TRUE   TRUE
## FOXP1     FALSE     TRUE             TRUE  FALSE
```

Regression models on the blood data.

```
burdens2test = c("burden_subs_passengers_blood")
outcomes = c(burdens2test, "alldrivers_blood", paste(genes2test_blood,"_blood",sep=""))
predictors = c("AGE","SEX","pack_years","drink_years","T2DM","BMI","cancer")

inds = which(!is.na(metadata$burden_subs_passengers_blood) & !(metadata$donor %in% other_excluded_samples_from_regressions) 
             & metadata$hpv==0 & metadata$chemo==0 & rowSums(is.na(metadata[,c(outcomes,predictors)]))==0) # Samples to be considered in the model

# Blood LMER on major epidemiological factors
regout = regfun(regdata=metadata[inds,], outcomes=outcomes, predictors=predictors, interactions=NULL, random_effects = "(1|twin)")
regression_heatmap(pvals=regout$pvals, qvals=regout$qvals, coeffs=regout$coeffs, filename = "Epidemiological_regressions_lmer_blood.pdf", maxval = 10, plotwidth = 4, plotheight = 4.5)
```

Plotting the increase in driver density and clone sizes with age.

```
genes2plot = c("DNMT3A","TET2")

# Mutations by decade
ages = setNames(metadata$AGE, substr(metadata$pd,1,7))[substr(unique(bloodmuts$sampleID),1,7)]
decades = setNames(cut(ages, breaks = seq(20,90,by=10), right = F), names(ages))
decades = decades[!is.na(decades)]
m = bloodmuts
m$decade = decades[substr(m$sampleID,1,7)]
bloodmuts_bydecade = split(m, f = m$decade)

# Initialising
drivage_stats = array(NA, dim=c(length(bloodmuts_bydecade), length(genes2plot), 9), 
                      dimnames = list(names(bloodmuts_bydecade), genes2plot, c("mean", "low", "high", "largestclone", "largestclonelow", "largestclonehigh", "dndsmean", "dndslow", "dndshigh")))
drivage_dndsglobal = array(NA, dim=c(length(bloodmuts_bydecade), 6), dimnames = list(names(bloodmuts_bydecade), c("wmis","wmislow","wmishigh","wnon","wnonlow","wnonhigh")))
decade_meanage = array(NA, length(bloodmuts_bydecade))

for (d in 1:length(bloodmuts_bydecade)) {
  
  decade_meanage[d] = mean(metadata$AGE[substr(metadata$pd,1,7) %in% substr(unique(bloodmuts_bydecade[[d]]$sampleID),1,7)])
  
  # Running dN/dS on individuals of that age
  dout = dndscv(mutations = bloodmuts_bydecade[[d]], gene_list = targetgenes, max_muts_per_gene_per_sample = Inf, max_coding_muts_per_sample = Inf, constrain_wnon_wspl = T, mingenecovs = 0, dc = gene2dc/mean(gene2dc), kc = newkc, outmats = T, onesided = T, cv = dndscovs, refdb = RefCDS, maxcovs = 10, use_indel_sites = use_indel_sites)
  ci = geneci(dout, gene_list = genes2plot)
  drivage_dndsglobal[d, 1:3] = as.numeric(dout$globaldnds[1,2:4])
  drivage_dndsglobal[d, 4:6] = as.numeric(dout$globaldnds[2,2:4])
  
  # Calculating summary metrics on driver frequency: (1) mean estimated driver fraction, (2) mean size of the largest clone per donor.
  maux = split(bloodmuts_bydecade[[d]], f = bloodmuts_bydecade[[d]]$sampleID)
  for (g in 1:length(genes2plot)) {
    drivfrac_vec = sapply(maux, function(x) sum(x$cellfraction[x$gene==genes2plot[g] & x$impact!="Synonymous"], na.rm=T))
    drivage_stats[d, g, 1:3] = meanci(drivfrac_vec)
    largestclone_vec = sapply(maux, function(x) maxorzero(x$bam_adj_cellfraction[x$gene==genes2plot[g] & x$impact!="Synonymous"], na.rm=T))
    drivage_stats[d, g, 4:6] = meanci(largestclone_vec)
    drivage_stats[d, g, 7:9] = as.numeric(ci[ci$gene==genes2plot[g],c(3,5,7)]) # MLEs and CI95% for wtru for each gene
  }
}

# Barplots of the increase in driver frequency with age
if (runman) { dev.new(width=7, height=7) }
par(mfrow=c(2,2))

b = barplot(drivage_stats[,,1]*100, beside=T, las=2, ylab="Estimated % mutant cells", col = hcl.colors(length(bloodmuts_bydecade), "Sunset"), ylim=c(0,max(drivage_stats[,,3]*100)))
segments(x0=b, y0=drivage_stats[,,2]*100, y1=drivage_stats[,,3]*100)
legend(x = mean(b)*1.4, y = max(drivage_stats[,,3]*100), legend = names(bloodmuts_bydecade), col = hcl.colors(length(bloodmuts_bydecade), "Sunset"), pch=15, box.col="white")

b = barplot(drivage_stats[,,7], beside=T, las=2, ylab="dN/dS truncating", col = hcl.colors(length(bloodmuts_bydecade), "Sunset"), ylim=c(0,max(drivage_stats[,,9])))
segments(x0=b, y0=drivage_stats[,,8], y1=drivage_stats[,,9])

b = barplot(t(drivage_stats[,,1]*100), las=2, ylab="Estimated % mutant cells", col = hcl.colors(length(genes2plot), "Temps"))
legend(x = 0, y = 25, legend = genes2plot, col = hcl.colors(length(genes2plot), "Temp"), pch=15, box.col=NA)

b = barplot(t(drivage_dndsglobal[,c(1,4)]), beside=T, las=2, col=c("cadetblue","darkorchid"), border=NA, ylim=c(0,max(drivage_dndsglobal[,6])), ylab="Global dN/dS")
abline(h=1)
segments(x0=b, y0=t(drivage_dndsglobal[,c(2,5)]), y1=t(drivage_dndsglobal[,c(3,6)]))
legend(x = 1, y = max(drivage_dndsglobal[,6]), legend = c("Missense","Nonsense"), col = c("cadetblue","darkorchid"), pch=15, box.col=NA)
```

```
if (runman) { dev.copy(pdf, "Increase_driver_density_age_blood.pdf", width=7, height=7); dev.off() }
```

Line plots of the increase in driver frequency with age, plotting each gene separately.

```
if (runman) { dev.new(width=length(genes2plot)*3.1, height=3.5) }
par(mfrow=c(1,length(genes2plot)))

for (j in 1:length(genes2plot)) {
  plot(x=decade_meanage, y=drivage_stats[,j,1], pch=19, col="lightslategrey", ylim=c(0,max(drivage_stats[,j,3])), main=genes2plot[j], las=1, xlab="Age",ylab="Estimated cell fraction", cex=0.8, xlim=c(20,90)) # Estimated driver fraction
  lines(x=decade_meanage, y=drivage_stats[,j,1], col="lightslategrey") # Connecting line
  segments(x0=decade_meanage, y0=drivage_stats[,j,2], y1=drivage_stats[,j,3], col="lightslategrey") # CI95%
  
  points(x=decade_meanage, y=drivage_stats[,j,4], pch=19, col="lightsalmon2") # Largest clone
  lines(x=decade_meanage, y=drivage_stats[,j,4], col="lightsalmon2") # Connecting line
  segments(x0=decade_meanage, y0=drivage_stats[,j,5], y1=drivage_stats[,j,6], col="lightsalmon2") # Largest clone CI95%
}
```

```
if (runman) { dev.copy(pdf, "Increase_driver_density_age_blood_lineplot.pdf", width=length(genes2plot)*3.1, height=3.5); dev.off() }
```

### VAF-based driver analyses

We can use VAFs to study whether driver mutations are associated with larger clones. This is expected for positively-selected mutations but the signal can be weak or undetectable when the clones are very small (very low true VAFs) compared to the available coverage. Nevertheless, we can check for evidence of higher VAFs for driver mutations using at least two different approaches:

1. Negative binomial regression on mutant reads vs coverage for putative driver and passenger mutations per gene.
2. Permutation test on VAFs, which can give more weight to the largest clones.

We restrict the analyses to coding SNVs, excluding essential splice sites and indels to minimise the risk of confounding effects from lower duplex coverage and underestimation of VAFs, respectively.

```
# Object with putative drivers and synonymous mutations to be used for the VAF analyses
genes2test = c(genes2test_ref,c("DNMT3A","TET2"))
cols = c("gene","chr","pos","mut","impact","duplex_vaf","bam_vaf","times_called","duplex_cov","bam_mut","bam_cov")
d = putativedrivmuts[which(putativedrivmuts$gene %in% genes2test & putativedrivmuts$impact %in% c("Missense","Nonsense","Essential_Splice")), cols]
d = rbind(d, mutations[which(mutations$gene %in% genes2test & mutations$impact=="Synonymous"), cols])
d = rbind(d, mutations[mutations$gene %in% c("DNMT3A","TET2") & mutations$impact %in% c("Missense","Nonsense","Essential_Splice","Synonymous"), cols]); d = unique(d) # Adding clonal haem drivers (we annotate any Miss or Non mutation as putative driver for this analysis as they do not always meet the criteria for inclusion)
d$isdriv = (d$impact!="Synonymous")
d$mstr = paste(d$chr, d$pos, d$mut, sep=":")
d$bam_mut_adj = pmax(0,d$bam_mut-d$times_called)
d$bam_cov_adj = d$bam_cov-d$duplex_cov
d$bam_vaf_adj = d$bam_mut_adj / d$bam_cov_adj
min_cov = 500; d = d[d$bam_cov_adj>=min_cov,]

# Excluding synonymous drivers
h = hotspots_allsites$recursites[hotspots_allsites$recursites$gene %in% genes2test & hotspots_allsites$recursites$impact=="Synonymous" & hotspots_allsites$recursites$qval<0.05,] 
h$mstr = paste(h$chr,h$pos,h$mut,sep=":")
d = d[!(d$mstr %in% h$mstr),] # Removing syn drivers
dsplit = split(d, f = d$gene)

# 1. Negative binomial regression test per gene: it seems to be more sensitive but it is based on stronger underlying assumptions that can be violated by the nature of the clonal expansions. We recommend the permutation test below as a more robust approach.
vafselection_glmnb = data.frame(gene=names(dsplit), glm_coeff=NA, glm_stderr=NA, glm_pval=NA)
for (j in 1:length(dsplit)) {
  coeffs = summary(MASS::glm.nb(bam_mut_adj ~ offset(log(bam_cov_adj)) + isdriv, data=dsplit[[j]]))$coefficients # Negative binomial regression
  #coeffs = summary(glm(bam_mut_adj ~ offset(log(bam_cov_adj)) + isdriv, data=dsplit[[j]], family = poisson()))$coefficients # Poisson regression
  vafselection_glmnb[j,2:4] = c(exp(coeffs[2,1:2]), coeffs[2,4])
}

# 2. Permutation test per gene

# Subfunction: based on Lawson et al, 2020 (PMID:33004514)
p.test = function(x, y, n=10000) {
  # Permutation test: ratio of mean VAFs
  st = mean(y)/mean(x)
  stvec = rep(NA,n); for (j in 1:n) { s = sample(c(x,y)); stvec[j] = mean(s[1:length(y)])/mean(s[(length(y)+1):length(s)]) }
  
  # Permutation test: ratio of Q3 VAFs
  #q = 0.75
  #st = quantile(y,q)/quantile(x,q)
  #stvec = rep(NA,n); for (j in 1:n) { s = sample(c(x,y)); stvec[j] = quantile(s[1:length(y)],q)/quantile(s[(length(y)+1):length(s)],q) }
  
  p = (sum(stvec>=st)+1)/(n+1)
  return(list(pval=p, st=st, randst95=quantile(stvec, c(0.025,0.975))))
}

vafselection_perm = data.frame(gene=names(dsplit), perm_mean=NA, perm_cilow=NA, perm_cihigh=NA, perm_pval=NA)
for (j in 1:length(dsplit)) {
  pass = dsplit[[j]]$bam_vaf_adj[dsplit[[j]]$isdriv==F]
  driv = dsplit[[j]]$bam_vaf_adj[dsplit[[j]]$isdriv==T]
  out = p.test(x=pass, y=driv, n=10000)
  vafselection_perm[j,2:5] = c(out$st, out$randst95, out$pval)
}

vafsel = cbind(vafselection_glmnb,vafselection_perm[,-1])
vafsel = vafsel[order(vafsel$perm_pval, vafsel$glm_pval), ]; rownames(vafsel) = NULL
print(vafsel)
```

```
##       gene glm_coeff glm_stderr     glm_pval perm_mean perm_cilow perm_cihigh
## 1   DNMT3A  4.253912   1.263985 6.401176e-10  4.121062  0.5964961    1.887203
## 2    ASXL1  1.891262   1.130794 2.169381e-07  1.797057  0.7430749    1.356823
## 3     TET2  2.661475   1.154319 9.044182e-12  2.634322  0.5572535    1.811186
## 4     FAT1  1.263892   1.064753 1.895071e-04  1.248543  0.8542779    1.171394
## 5     TP53  1.824004   1.208340 1.493628e-03  1.731463  0.6053097    1.528263
## 6   NOTCH1  1.236212   1.075533 3.589362e-03  1.245506  0.8519344    1.178879
## 7    CHEK2  1.742329   1.259089 1.595485e-02  1.788203  0.6323370    1.681057
## 8     RAC1  2.035006   1.443844 5.307194e-02  2.184745  0.5255353    2.430070
## 9   BCORL1  1.129793   1.107943 2.338423e-01  1.180828  0.8067284    1.242965
## 10   PPM1D  1.627261   1.251831 3.017594e-02  1.569327  0.4436815    1.839607
## 11  NOTCH2  1.104063   1.117818 3.740934e-01  1.127086  0.7556161    1.316816
## 12 ZFP36L2  1.058471   1.252465 8.007101e-01  1.074599  0.6026550    1.544145
##     perm_pval
## 1  0.00009999
## 2  0.00019998
## 3  0.00029997
## 4  0.00319968
## 5  0.00439956
## 6  0.00459954
## 7  0.01449855
## 8  0.04119588
## 9  0.06989301
## 10 0.10688931
## 11 0.21537846
## 12 0.36636336
```

We can then run an analogous analysis for the blood data.

```
# Object with putative drivers and synonymous mutations to be used for the VAF analyses
genes2test = genes2test_blood
cols = c("gene","chr","pos","mut","impact","duplex_vaf","bam_vaf","times_called","duplex_cov","bam_mut","bam_cov")
d = putativedrivmuts_blood[which(putativedrivmuts_blood$gene %in% genes2test & putativedrivmuts_blood$impact %in% c("Missense","Nonsense","Essential_Splice")), cols]
d = rbind(d, bloodmuts[which(bloodmuts$gene %in% genes2test & bloodmuts$impact=="Synonymous"), cols])
d$isdriv = (d$impact!="Synonymous")
d$mstr = paste(d$chr, d$pos, d$mut, sep=":")
d$bam_mut_adj = pmax(0,d$bam_mut-d$times_called)
d$bam_cov_adj = d$bam_cov-d$duplex_cov
d$bam_vaf_adj = d$bam_mut_adj / d$bam_cov_adj
min_cov = 500; d = d[d$bam_cov_adj>=min_cov,]
dsplit = split(d, f = d$gene)

# 1. Negative binomial regression test per gene: it seems to be more sensitive but it is based on stronger underlying assumptions that can be violated by the nature of the clonal expansions. We recommend the permutation test below as a more robust approach.
vafselection_glmnb_blood = data.frame(gene=names(dsplit), glm_coeff=NA, glm_stderr=NA, glm_pval=NA)
for (j in 1:length(dsplit)) {
  coeffs = summary(MASS::glm.nb(bam_mut_adj ~ offset(log(bam_cov_adj)) + isdriv, data=dsplit[[j]]))$coefficients # Negative binomial regression
  vafselection_glmnb_blood[j,2:4] = c(exp(coeffs[2,1:2]), coeffs[2,4])
}

# 2. Permutation test per gene
vafselection_perm_blood = data.frame(gene=names(dsplit), perm_mean=NA, perm_cilow=NA, perm_cihigh=NA, perm_pval=NA)
for (j in 1:length(dsplit)) {
  pass = dsplit[[j]]$bam_vaf_adj[dsplit[[j]]$isdriv==F]
  driv = dsplit[[j]]$bam_vaf_adj[dsplit[[j]]$isdriv==T]
  out = p.test(x=pass, y=driv, n=10000)
  vafselection_perm_blood[j,2:5] = c(out$st, out$randst95, out$pval)
}

vafsel = cbind(vafselection_glmnb_blood,vafselection_perm_blood[,-1])
vafsel = vafsel[order(vafsel$perm_pval, vafsel$glm_pval), ]; rownames(vafsel) = NULL
print(vafsel)
```

```
##     gene glm_coeff glm_stderr     glm_pval perm_mean perm_cilow perm_cihigh
## 1   TET2  4.569729   1.323429 5.885511e-08  4.665204 0.34857323    3.563338
## 2  CHEK2  7.351807   2.585877 3.574641e-02  7.026843 0.08418885   20.903839
## 3  PPM1D  4.786546   2.295590 5.952853e-02  4.643885 0.05324947   26.729821
## 4 DNMT3A  1.282120   1.525048 5.559537e-01  1.276610 0.30241837    4.674085
##    perm_pval
## 1 0.00369963
## 2 0.15588441
## 3 0.42105789
## 4 0.51634837
```

### Driver-generation potential for signature B (SBS16-like)

The epidemiological regressions suggest that smoking and alcohol contribute similar numbers of mutations to our dataset. However, on the basis of the glm p-values, pack years appear to be more strongly associated with driver densities than drink years. This could have multiple explanations, including a different selectogenic/promoter effect of smoking or alcohol, confounding effects not accounted for by the model, or other factors. One additional explanation is that signature B may have lower driver-generation potential than signature A. Since signature B mainly generates two types of SNVs (T>C at ATA and ATT trinucleotides), out of all possible 192 changes, and the signature is correlated with high gene expression, it is possible that its ability to generate non-synonymous mutations in some of the main driver genes may be lower. Here we explore this possibility using 2 analyses:

1. Comparing the frequency of signature B mutations in coding vs intronic/UTR regions.
2. Comparing the tendency (under neutrality) of signature A and B to generate missense, nonsense and essential splice site mutations in the top driver genes.

```
# 1. List of donors with high and low SigB
sb = metadata$sig_denovo_sigB/(metadata$sig_denovo_sigB+metadata$sig_denovo_sigA)
pds_low = metadata$pd[which(sb<=quantile(sb,0.05,na.rm=T))]
pds_high = metadata$pd[which(sb>=quantile(sb,0.95,na.rm=T))]

## 1. Fraction of coding vs intronic/UTR mutations that are in the 2 main SBS16 peaks
genes2test = genes2test_ref
#genes2test = c("NOTCH1","TP53","CHEK2")
trin = c("ATT>C","ATA>C")

# Function to output binomial confidence intervals for a T/F vector
binfrac = function(x) {
  bt = binom.test(sum(x),length(x))
  return(as.numeric(c(bt$estimate,bt$conf.int)))
}
fracmat = data.frame(test=c("Non-coding SNVs","Coding all genes","Coding top drivers","Non-coding SNVs","Coding all genes","Coding top drivers"), f=NA, cilow=NA, cihigh=NA)
# SigB-high samples
fracmat[1,2:4] = binfrac(mutations$tri[which(mutations$sampleID %in% pds_high & !is.na(mutations$tri) & is.na(mutations$impact))] %in% trin) # Non-coding SNVs
fracmat[2,2:4] = binfrac(mutations$tri[which(mutations$sampleID %in% pds_high & !is.na(mutations$tri) & !is.na(mutations$impact))] %in% trin) # All coding SNVs
fracmat[3,2:4] = binfrac(mutations$tri[which(mutations$sampleID %in% pds_high & !is.na(mutations$tri) & mutations$gene %in% genes2test)] %in% trin) # Coding SNVs in top drivers
# SigB-low samples
fracmat[4,2:4] = binfrac(mutations$tri[which(mutations$sampleID %in% pds_low & !is.na(mutations$tri) & is.na(mutations$impact))] %in% trin) # Non-coding SNVs
fracmat[5,2:4] = binfrac(mutations$tri[which(mutations$sampleID %in% pds_low & !is.na(mutations$tri) & !is.na(mutations$impact))] %in% trin) # All coding SNVs
fracmat[6,2:4] = binfrac(mutations$tri[which(mutations$sampleID %in% pds_low & !is.na(mutations$tri) & mutations$gene %in% genes2test)] %in% trin) # Coding SNVs in top drivers

if (runman) { dev.new(width=8, height=5) }
par(mfrow=c(1,2))

h = barplot(fracmat$f, ylim=c(0,0.22), col=c(rep("indianred",3),rep("lightslategray",3)), border = NA, las=2, names.arg = fracmat$test, 
            ylab = sprintf("Fraction %s",paste(c("ATT>C","ATA>C"), collapse = "|")))
segments(x0=h, y0=fracmat$cilow, y1=fracmat$cihigh)
segments(x0=h[c(1,4)], x1=h[c(3,6)], y0=c(0.2,0.1)); text(x=h[c(2,5)], y = c(0.21,0.11), labels = c("High SigB samples","Low SigB samples"), cex=0.8, adj=c(0.5,0.5))

## 2. Potential of Signature B peaks to generate non-synonymous mutations

Ldriv = apply(dndsout$L[,,dndsout$genemuts$gene_name %in% genes2test], c(1,2), sum) # Cumulative L matrix of the chosen genes

# Adding row names to the L matrix
nt = c("A","C","G","T")
trinucs = paste(rep(nt,each=16,times=1),rep(nt,each=4,times=4),rep(nt,each=1,times=16), sep="")
trinucsubs = NULL
for (j in 1:length(trinucs)) { trinucsubs = c(trinucsubs, paste(trinucs[j], paste(substr(trinucs[j],1,1), setdiff(nt,substr(trinucs[j],2,2)), substr(trinucs[j],3,3), sep=""), sep=">")) }
rownames(Ldriv) = trinucsubs

# Relative driver-generation abilities
allsites = colSums(Ldriv); allsites = allsites/sum(allsites)
sigBsites = colSums(Ldriv[c("ATA>ACA","ATT>ACT"),]); sigBsites = sigBsites/sum(sigBsites)

h = barplot(rbind(allsites,sigBsites), beside = T, las=2, names.arg = c("Synonymous","Missense","Nonsense","Splice"), col=c("grey30","grey70"), border=NA, ylab="Impact probability per mutation")
legend(x=5.5, y=0.7, legend=c("All trinucleotides","ATA>ACA & ATT>ACT"), box.col = NA, pch=15, col=c("grey30","grey70"))
```

```
if (runman) { dev.copy(pdf, file = "SigB_driver_generation_potential.pdf", width=8, height=5); dev.off() }
```

We can then generate mutational spectra for the non-exonic and drivergene-exonic mutations in high-sigB donors using the function below.

```
draw_cosmicsig = function(muts, outfilename="spectrum_cosmiclike.pdf", normvec=NULL) {
  
  muts = muts[which(muts$type=="snv"),]
  muts$sub = paste(substr(muts$tri,2,2),substr(muts$tri,5,5),sep=">")
  freqs = table(paste(muts$sub, paste(substr(muts$tri,1,1),substr(muts$tri,3,3),sep="-"),sep=","))
  
  sub_vec = c("C>A","C>G","C>T","T>A","T>C","T>G")
  ctx_vec = paste(rep(c("A","C","G","T"),each=4),rep(c("A","C","G","T"),times=4),sep="-")
  full_vec = paste(rep(sub_vec,each=16),rep(ctx_vec,times=6),sep=",")
  freqs_full = freqs[full_vec]; freqs_full[is.na(freqs_full)] = 0; names(freqs_full) = full_vec
  
  if (!is.null(normvec)) {
    freqs_full = freqs_full * normvec
  }
  
  xstr = paste(substr(full_vec,5,5), substr(full_vec,1,1), substr(full_vec,7,7), sep="")
  
  if (runman) { dev.new(width=10,height=4) }
  colvec = rep(c("dodgerblue","black","red","grey70","olivedrab3","plum2"),each=16)
  y = freqs_full; maxy = max(y)
  h = barplot(y, las=2, col=colvec, border=NA, ylim=c(0,maxy*1.5), space=1, cex.names=0.6, names.arg=xstr, ylab="Number mutations")
  for (j in 1:length(sub_vec)) {
    xpos = h[c((j-1)*16+1,j*16)]
    rect(xpos[1]-0.5, maxy*1.2, xpos[2]+0.5, maxy*1.3, border=NA, col=colvec[j*16])
    text(x=mean(xpos), y=maxy*1.3, pos=3, label=sub_vec[j])
  }    
  if (runman) { dev.copy(pdf,outfilename,width=10,height=4); dev.off(); dev.off() }
}
```

Noncoding mutations in high signature B samples.

```
muts = mutations[which(mutations$sampleID %in% pds_high & !is.na(mutations$tri) & is.na(mutations$impact)), ] # Non-coding SNVs
draw_cosmicsig(muts, "spectrum_high_signatureB_noncoding.pdf")
```

Exonic mutations in driver genes in high signature B samples.

```
muts = mutations[which(mutations$sampleID %in% pds_high & !is.na(mutations$tri) & mutations$gene %in% genes2test), ] # Coding SNVs in top drivers
draw_cosmicsig(muts, "spectrum_high_signatureB_exonic_drivergenes.pdf")
```

Noncoding mutations in low signature B samples.

```
muts = mutations[which(mutations$sampleID %in% pds_low & !is.na(mutations$tri) & is.na(mutations$impact)), ] # Non-coding SNVs
draw_cosmicsig(muts, "spectrum_low_signatureB_noncoding.pdf")
```

Exonic mutations in driver genes in low signature B samples.

```
muts = mutations[which(mutations$sampleID %in% pds_low & !is.na(mutations$tri) & mutations$gene %in% genes2test), ] # Coding SNVs in top drivers
draw_cosmicsig(muts, "spectrum_low_signatureB_exonic_drivergenes.pdf")
```

### Additional plots of mutational spectra

Signature A spectrum.

```
spectra_sigs = read.table(signatures_spectra_file, header=1, sep="\t", stringsAsFactors=F)

sub_vec = c("C>A","C>G","C>T","T>A","T>C","T>G")
ctx_vec = paste(rep(c("A","C","G","T"),each=4),rep(c("A","C","G","T"),times=4),sep="-")
full_vec = paste(rep(sub_vec,each=16),rep(ctx_vec,times=6),sep=",")
freqs_full = as.numeric(spectra_sigs[1,])
names(freqs_full) = full_vec
xstr = paste(substr(full_vec,5,5), substr(full_vec,1,1), substr(full_vec,7,7), sep="")
  
if (runman) { dev.new(width=10,height=4) }
colvec = rep(c("dodgerblue","black","red","grey70","olivedrab3","plum2"),each=16)
y = freqs_full; maxy = max(y)
h = barplot(y, las=2, col=colvec, border=NA, ylim=c(0,maxy*1.5), space=1, cex.names=0.6, names.arg=xstr, ylab="Number mutations")
for (j in 1:length(sub_vec)) {
  xpos = h[c((j-1)*16+1,j*16)]
  rect(xpos[1]-0.5, maxy*1.2, xpos[2]+0.5, maxy*1.3, border=NA, col=colvec[j*16])
  text(x=mean(xpos), y=maxy*1.3, pos=3, label=sub_vec[j])
}
```

```
if (runman) { dev.copy(pdf,"Signature_A_spectrum.pdf",width=10,height=4); dev.off(); dev.off() }
```

Signature B spectrum.

```
freqs_full = setNames(as.numeric(spectra_sigs[2,]), full_vec)

if (runman) { dev.new(width=10,height=4) }
y = freqs_full; maxy = max(y)
h = barplot(y, las=2, col=colvec, border=NA, ylim=c(0,maxy*1.5), space=1, cex.names=0.6, names.arg=xstr, ylab="Number mutations")
for (j in 1:length(sub_vec)) {
  xpos = h[c((j-1)*16+1,j*16)]
  rect(xpos[1]-0.5, maxy*1.2, xpos[2]+0.5, maxy*1.3, border=NA, col=colvec[j*16])
  text(x=mean(xpos), y=maxy*1.3, pos=3, label=sub_vec[j])
}
```

```
if (runman) { dev.copy(pdf,"Signature_B_spectrum.pdf",width=10,height=4); dev.off(); dev.off() }
```

Aggregate spectra for heavy-drinking and heavy-smoking donors.

```
# Calculating the correction vector (ratio of trinucleotide frequencies from whole-genome to targeted regions)
trinucs_tg = read.table(trinuc_frequencies_sbs96_TG_file, header=1, sep="\t", stringsAsFactors=F)
trinucs_wg = read.table(trinuc_frequencies_sbs96_WG_file, header=1, sep="\t", stringsAsFactors=F)
tg2wg_normvec = as.numeric(trinucs_wg)/as.numeric(trinucs_tg)
tg2wg_normvec = tg2wg_normvec/mean(tg2wg_normvec)

# Smokers and drinkers 
smoke_high = metadata$pd[which(metadata$pack_years>=20)]
smoke_never = metadata$pd[which(metadata$pack_years==0)]
drink_high = metadata$pd[which(metadata$drink_years>=180)] # Approx equivalent to 25 years drinking 50 units per week
drink_never = metadata$pd[which(metadata$drink_years==0)]

muts = mutations[which(mutations$sampleID %in% intersect(smoke_high,drink_high)), ] # Heavy smokers and heavy drinkers
draw_cosmicsig(muts, "spectrum_heavysmoker_heavydrinker.pdf", normvec = tg2wg_normvec)
```

Aggregate spectra for heavy-drinking and never-smoking donors.

```
muts = mutations[which(mutations$sampleID %in% intersect(smoke_never,drink_high)), ] # Never smokers and heavy drinkers
draw_cosmicsig(muts, "spectrum_neversmoker_heavydrinker.pdf", normvec = tg2wg_normvec)
```

Aggregate spectra for never-drinking and never-smoking donors. Notice, however, that never drinking is likely poorly estimated.

```
muts = mutations[which(mutations$sampleID %in% intersect(smoke_never,drink_never)), ] # Never smokers and never drinkers
draw_cosmicsig(muts, "spectrum_neversmoker_neverdrinker.pdf", normvec = tg2wg_normvec)
```

Aggregate spectra for never-drinking and heavy-smoking donors. Notice, however, that never drinking is likely poorly estimated.

```
muts = mutations[which(mutations$sampleID %in% intersect(smoke_high,drink_never)), ] # Never smokers and never drinkers
draw_cosmicsig(muts, "spectrum_heavysmoker_neverdrinker.pdf", normvec = tg2wg_normvec)
```

### Interaction between alcohol and smoking on Signature B rates

Multiple epidemiological studies have found significant synergistic interactions between smoking and alcohol consumption on the risk of multiple cancer types, including oral cancer, oesophageal cancer and head and neck cancers (e.g. PMID:19190158, PMID:17373408). In the absence of interaction terms, our regression models above found a significant association of both smoking and alcohol consumption with Signature B (SBS16) rate, with alcohol showing the strongest association. Somatic mutation studies of oesophageal squamous carcinomas (PMID:28548104) and normal oesophagus (PMID:30602793), have reported an association of SBS16 with germline polymorphisms in the ALDH2 gene (aldehyde dehydrogenase) suggesting a mechanistic link between alcohol (and its metabolite acetaldehyde) and SBS16. The association of smoking of with Signature B in our oral epithelium data could be caused by at least 3 non-mutually-exclusive explanations:

1. A direct effect of smoking on Signature B, perhaps through acetaldehyde or other mutagens present in tobacco smoke or its metabolites (PMID:17590988).
2. An interaction effect between smoking and alcohol, where smoking could increase the mutagenic effect of alcohol consumption.
3. Finally, the correlation of pack-years with Signature B could be partially contributed by the inaccuracy of our drink-year estimates, which are estimated by extrapolating recent consumption. Since smoking and alcohol consumption habits are strongly correlated in our data, inaccurate/incomplete alcohol consumption data could lead to a residual correlation of Signature B with smoking, independent of any direct or interaction effects of smoking.

To explore these possibilities, we run a number of additional analyses below. First, we use an LMER model with an interaction term between pack\_years and drink\_years. To do so, we apply Z-score normalisation of the variables in the model (dependent and independent). The analysis below does not find a significant interaction between pack\_years and drink\_years, but it could be affected by the skewness of both variables, and by possible nonlinear interaction effects.

```
outcomes = c("sig_denovo_sigB")
predictors = c("AGE","SEX","T2DM","BMI","missingteeth","ipaq_score","cancer","pack_years","drink_years")
inds = which(metadata$duplex_cov>=min_duplexcov & metadata$AGE>=min_age & !(metadata$donor %in% other_excluded_samples_from_regressions) 
             & metadata$hpv==0 & metadata$chemo==0 & rowSums(is.na(metadata[,c(outcomes,predictors)]))==0) # Samples to be considered in the model

regout = regfun(regdata=metadata[inds,], outcomes=outcomes, predictors=predictors, interactions=c("drink_years:pack_years"), random_effects = "(1|twin)", zscore = T)
print(data.frame(variable=rownames(regout$pvals),pvals=as.numeric(regout$pvals), qvals=as.numeric(regout$qvals)))
```

```
##                  variable        pvals        qvals
## 1                     AGE 5.452976e-02 1.363244e-01
## 2                     SEX 1.742180e-01 3.484361e-01
## 3                    T2DM 9.174735e-01 9.213847e-01
## 4                     BMI 9.213847e-01 9.213847e-01
## 5            missingteeth 3.140541e-01 5.234235e-01
## 6              ipaq_score 6.703703e-01 8.379628e-01
## 7                  cancer 5.854671e-01 8.363816e-01
## 8              pack_years 4.949675e-06 2.474837e-05
## 9             drink_years 6.013671e-15 6.013671e-14
## 10 pack_years:drink_years 4.940202e-02 1.363244e-01
```

As an alternative approach to explore an interaction between pack\_years and drink\_years, we discretise smokers into groups (never smokers -pack-years = 0-, light smokers -1-20- and moderate/heavy smokers ->20-), reducing the effect of extreme outliers and a reliance on linearity assumptions. This analysis finds evidence of a significant interaction effect, where the increase in Signature B per drink year is higher with smoking (and seemingly higher with heavy/moderate smoking than with light smoking). This interaction is stronger when not including pack\_years as an additional independent term, but is significant in both cases.

Whereas this analysis is consistent with an interaction between smoking and alcohol in causing Signature B, and could offer a mechanistic explanation for the observed interaction of both factors in oral cancer risk, we cannot rule out a true independent effect or a confounder effect of inaccurate metadata.

```
metadata$smoking_group = NA
metadata$smoking_group[metadata$pack_years==0] = "0"
metadata$smoking_group[metadata$pack_years>0 & metadata$pack_years<=20] = "0-20"
metadata$smoking_group[metadata$pack_years>20] = "20+"

# GLM with a different slope for the effect of drink_years for each smoker bin
model = glm(sig_denovo_sigB ~ AGE + SEX + T2DM + BMI + missingteeth + ipaq_score + cancer + pack_years + drink_years:smoking_group, data=metadata[inds,])
print(summary(model))
```

```
## 
## Call:
## glm(formula = sig_denovo_sigB ~ AGE + SEX + T2DM + BMI + missingteeth + 
##     ipaq_score + cancer + pack_years + drink_years:smoking_group, 
##     data = metadata[inds, ])
## 
## Coefficients:
##                                 Estimate Std. Error t value Pr(>|t|)    
## (Intercept)                    1.212e-08  1.028e-08   1.179   0.2388    
## AGE                            2.226e-10  9.654e-11   2.306   0.0214 *  
## SEXM                           4.945e-09  3.278e-09   1.509   0.1319    
## T2DM                          -2.872e-10  3.791e-09  -0.076   0.9396    
## BMI                            9.377e-12  2.311e-10   0.041   0.9676    
## missingteeth                   2.026e-09  1.806e-09   1.122   0.2623    
## ipaq_score                    -2.614e-10  1.808e-09  -0.145   0.8851    
## cancer                        -1.981e-09  2.835e-09  -0.699   0.4848    
## pack_years                     3.303e-10  1.162e-10   2.843   0.0046 ** 
## drink_years:smoking_group0     9.018e-11  2.111e-11   4.272 2.20e-05 ***
## drink_years:smoking_group0-20  1.556e-10  1.964e-11   7.922 8.81e-15 ***
## drink_years:smoking_group20+   1.672e-10  2.653e-11   6.303 5.06e-10 ***
## ---
## Signif. codes:  0 '***' 0.001 '**' 0.01 '*' 0.05 '.' 0.1 ' ' 1
## 
## (Dispersion parameter for gaussian family taken to be 9.153368e-16)
## 
##     Null deviance: 8.2054e-13  on 737  degrees of freedom
## Residual deviance: 6.6453e-13  on 726  degrees of freedom
## AIC: -23447
## 
## Number of Fisher Scoring iterations: 1
```

```
# LMER interaction test not including pack-years as an independent variable
model1 = lme4::lmer(sig_denovo_sigB ~ AGE + SEX + T2DM + BMI + missingteeth + ipaq_score + cancer + drink_years:smoking_group + (1|twin), data=metadata[inds,], REML=F)
model0 = lme4::lmer(sig_denovo_sigB ~ AGE + SEX + T2DM + BMI + missingteeth + ipaq_score + cancer + drink_years + (1|twin), data=metadata[inds,], REML=F)
print(anova(model0,model1))
```

```
## Data: metadata[inds, ]
## Models:
## model0: sig_denovo_sigB ~ AGE + SEX + T2DM + BMI + missingteeth + ipaq_score + cancer + drink_years + (1 | twin)
## model1: sig_denovo_sigB ~ AGE + SEX + T2DM + BMI + missingteeth + ipaq_score + cancer + drink_years:smoking_group + (1 | twin)
##        npar    AIC    BIC logLik deviance  Chisq Df Pr(>Chisq)    
## model0   11 -23447 -23396  11734   -23469                         
## model1   13 -23468 -23408  11747   -23494 24.466  2  4.867e-06 ***
## ---
## Signif. codes:  0 '***' 0.001 '**' 0.01 '*' 0.05 '.' 0.1 ' ' 1
```

```
# LMER interaction test including pack-years as an independent variable
model1 = lme4::lmer(sig_denovo_sigB ~ AGE + SEX + T2DM + BMI + missingteeth + ipaq_score + cancer + pack_years + drink_years:smoking_group + (1|twin), data=metadata[inds,], REML=F)
model0 = lme4::lmer(sig_denovo_sigB ~ AGE + SEX + T2DM + BMI + missingteeth + ipaq_score + cancer + pack_years + drink_years + (1|twin), data=metadata[inds,], REML=F)
print(anova(model0,model1))
```

```
## Data: metadata[inds, ]
## Models:
## model0: sig_denovo_sigB ~ AGE + SEX + T2DM + BMI + missingteeth + ipaq_score + cancer + pack_years + drink_years + (1 | twin)
## model1: sig_denovo_sigB ~ AGE + SEX + T2DM + BMI + missingteeth + ipaq_score + cancer + pack_years + drink_years:smoking_group + (1 | twin)
##        npar    AIC    BIC logLik deviance  Chisq Df Pr(>Chisq)   
## model0   12 -23468 -23413  11746   -23492                        
## model1   14 -23474 -23410  11751   -23502 10.241  2   0.005973 **
## ---
## Signif. codes:  0 '***' 0.001 '**' 0.01 '*' 0.05 '.' 0.1 ' ' 1
```

As a complementary test, the code below performs some analyses on how well our drink\_year estimate based on recent consumption recapitulates lifetime-consumption data available from a smaller subset of donors. The first analysis below is a regression including drink\_years (estimated from recent consumption) and total\_consumption (an ordinal lifetime estimate) to assess how well they explain Signature B rates. This shows that drink\_years is a much better predictor, with total\_consumption seemingly not capturing additional information. This, and the fact that total\_consumption was available for a minority of individuals, was the basis for using drink\_years in all our analyses.

```
# LMER using drink_years (extrapolated from recent consumption) and total_consumption (from lifetime alcohol consumption questions)
outcomes = c("burden_subs_passengers", "sig_denovo_sigA", "sig_denovo_sigB", "burden_indels_passengers", "dnv_burden")
predictors = c("AGE","SEX","pack_years","drink_years","total_consumption","T2DM","BMI","missingteeth","ipaq_score","cancer")
inds = which(metadata$duplex_cov>=min_duplexcov & metadata$AGE>=min_age & !(metadata$donor %in% other_excluded_samples_from_regressions) 
             & metadata$hpv==0 & metadata$chemo==0 & rowSums(is.na(metadata[,c(outcomes,predictors)]))==0) # Samples to be considered in the model
regout = regfun(regdata=metadata[inds,], outcomes=outcomes, predictors=predictors, interactions=NULL, random_effects = "(1|twin)")
regression_heatmap(pvals=regout$pvals, qvals=regout$qvals, coeffs=regout$coeffs, filename = "Epidemiological_regressions_lmer_drinkyears_vs_totalconsumption.pdf", maxval = 10, plotwidth = 4.5, plotheight = 3.5)
```

An additional analysis below shows a comparison between drink\_years and total\_consumption showing a reasonable level of concordance. In particular, only a small fraction of donors with moderate/high alcohol lifetime consumption (as recorded in the “total\_consumption” field) report no recent alcohol consumption and have an estimated drink\_years of 0.

```
if (runman) { dev.new(width=7, height=4.5) }
par(mfrow=c(1,2))

# Boxplot drink_years~total_consumption
metadata$total_consumption_binned = ceiling(metadata$total_consumption)
metadata$total_consumption_binned[metadata$total_consumption_binned>=4] = 4 # Capped to 4 as there are too few donors
boxplot(metadata$drink_years~metadata$total_consumption_binned, notch=T, las=1, outline=F,
        xlab="Lifetime consumption (binned)", ylab="Estimated drink-years from recent consumption")
warning("Boxplot plotted excluding outliers")
```

```
## Warning: Boxplot plotted excluding outliers
```

```
# Barplot fraction of drink-years=0 per total_consumption group
x0 = table(metadata$total_consumption_binned[metadata$drink_years==0])[as.character(1:4)]
xall = table(metadata$total_consumption_binned[!is.na(metadata$drink_years)])[as.character(1:4)]
ci = sapply(1:length(x0), function(j) binom.test(x=x0[j],n=xall[j])$conf.int)
h = barplot(x0/xall, col="grey60", border=NA, ylab="Fraction of donors with drink_years=0", xlab="Lifetime consumption (binned)", ylim=c(0,max(ci)), las=1)
segments(x0=h, y0=ci[1,], y1=ci[2,], col="black")
```

```
if (runman) { dev.copy(pdf, "Alcohol_drink_years_vs_total_consumption.pdf", width=7, height=4.5); dev.off() }
```

Finally, we repeat the regression analysis with an interaction term excluding individuals with drink\_years=0 AND total\_consumption>1, in an attempt to remove former drinkers or individuals with inconsistent reporting. This reduces our statistical power considerably as the number of donors with lifetime consumption information is limited. This analysis still finds a significant interaction effect, although it also detects an additional effect for pack-years as an independent variable. Collectively, the analyses above offer some support to the hypothesis that smoking may induce SBS16 by exacerbating the effects of alcohol, but caution should be exercised given the imperfect nature of the available metadata.

```
inds = which(metadata$duplex_cov>=min_duplexcov & metadata$AGE>=min_age & !(metadata$donor %in% other_excluded_samples_from_regressions) 
             & metadata$hpv==0 & metadata$chemo==0 & rowSums(is.na(metadata[,c(outcomes,predictors)]))==0) # Samples to be considered in the model
inds = setdiff(inds,which(metadata$drink_years==0 & metadata$total_consumption>1)) # Excluding inconsistent donors
message(sprintf("Analysis limited to %0.0f donors with sufficient metadata.",length(inds)))
```

```
## Analysis limited to 202 donors with sufficient metadata.
```

```
# LMER interaction test  including pack-years as an independent variable
model1 = lme4::lmer(sig_denovo_sigB ~ AGE + SEX + T2DM + BMI + missingteeth + ipaq_score + cancer + pack_years + drink_years:smoking_group + (1|twin), data=metadata[inds,], REML=F)
model0 = lme4::lmer(sig_denovo_sigB ~ AGE + SEX + T2DM + BMI + missingteeth + ipaq_score + cancer + pack_years + drink_years + (1|twin), data=metadata[inds,], REML=F)
```

```
## boundary (singular) fit: see help('isSingular')
```

```
print(anova(model0,model1))
```

```
## Data: metadata[inds, ]
## Models:
## model0: sig_denovo_sigB ~ AGE + SEX + T2DM + BMI + missingteeth + ipaq_score + cancer + pack_years + drink_years + (1 | twin)
## model1: sig_denovo_sigB ~ AGE + SEX + T2DM + BMI + missingteeth + ipaq_score + cancer + pack_years + drink_years:smoking_group + (1 | twin)
##        npar     AIC     BIC logLik deviance  Chisq Df Pr(>Chisq)  
## model0   12 -6365.8 -6326.1 3194.9  -6389.8                       
## model1   14 -6370.3 -6324.0 3199.2  -6398.3 8.4701  2    0.01448 *
## ---
## Signif. codes:  0 '***' 0.001 '**' 0.01 '*' 0.05 '.' 0.1 ' ' 1
```

```
packyear_pval = drop1(model1, test = "Chisq", scope = "pack_years")
message(sprintf("Pack year P-value: %0.3g",packyear_pval[-1,4]))
```

```
## Pack year P-value: 0.46
```

Of note, analysis of the mutational spectra of non-drinking smokers, annotated on the basis of both drink\_years and total\_consumption, also shows limited evidence of Signature B, further supporting the hypothesis that smoking may contribute to Signature B through an interaction with alcohol.

```
drink_never_total = intersect(drink_never, metadata$pd[which(metadata$total_consumption==1)])
smoke_moderatehigh = metadata$pd[which(metadata$pack_years>=5)]
muts = mutations[which(mutations$sampleID %in% intersect(smoke_moderatehigh,drink_never_total)), ] # Never smokers and never drinkers
draw_cosmicsig(muts, "spectrum_heavymoderatesmoker_neverdrinkertotalconsumption_and_drinkyears.pdf", normvec = tg2wg_normvec)
```

## 3. Heritability analyses using residuals

As an initial approach to understand whether MZ, DZ and random age-matched pairs of individuals are more similar to one another than expected by chance, we use the residuals of the glm regression models. This is complemented by more formal heritability analyses in a separate analysis.

```
# Function to calculate the residuals for variables of interest (burdencov indicates whether burden should be used as a covariate, eg. to remove the effect of mutation on driver heritability)
get_residuals = function(md, varname, burdencov=F, isblood = F, min_duplexcov = 50, min_age = 0) {
  # Regression model
  inds = which(md$duplex_cov>=min_duplexcov & md$AGE>=min_age & !(md$donor %in% other_excluded_samples_from_regressions)
               & !is.na(md$pack_years) & !is.na(md$drink_years) & !is.na(md$AGE) & !is.na(md[,varname]))
  if (burdencov==T) {
    if (isblood==F) {
      model = glm(md[inds,varname] ~ AGE + SEX + pack_years + drink_years + burden_subs_passengers, data = md[inds,]) # Using buccal burden as a covariate
    } else {
      model = glm(md[inds,varname] ~ AGE + SEX + pack_years + drink_years + burden_subs_passengers_blood, data = md[inds,]) # Using blood burden as a covariate
    }
  } else {
    model = glm(md[inds,varname] ~ AGE + SEX + pack_years + drink_years, data = md[inds,])
  }
  #model = glm(md[inds,varname] ~ AGE, data = md[inds,]) # Correcting only for age
  # Residuals
  res = model$residuals
  md[names(res),sprintf("%s_res",varname)] = res
  return(md)
}

# Function to calculate the average adsolute distance of residuals for donors of the same age but not twins
absdiff_sameage = function(md, varname) {
  
  md = md[which(!duplicated(md$twin)),] # Selecting the first twin of each pair
  md = md[!is.na(md[,varname]), ]
  sameage = split(md, f=paste(md$AGE,md$SEX)) # Grouping unrelated donors by age and sex
  sameage = sameage[names(which(sapply(sameage, nrow)>1))] # Selecting only ages with multiple donors
  
  #diffs = unlist(sapply(sameage, function(x) as.vector(dist(x[,varname], method = "manhattan")))) # All-against-all pairwise differences (this multiple counts each data point when the group size is >2)
  diffs = unlist(sapply(sameage, function(x) abs(diff(sample(x[,varname]))[seq(1,nrow(x)-1,by=2)]))) # Random pairs of unrelated donors, randomly using each donor in only one pair
  diffs = diffs[!is.na(diffs)]
  
  # Returning the vector of diffs
  return(diffs)
  
  # Returning the median and CI95% of the median (using the boxplot equation: median +/- 1.58*IQR/sqrt(n))
  #iqr = abs(diff(quantile(diffs, c(0.25, 0.75)))) # Interquantile range
  #return(c(median(diffs), median(diffs)-1.58*iqr/sqrt(length(diffs)), median(diffs)+1.58*iqr/sqrt(length(diffs)))) 
}

# Calculating residuals for several variables of interest
aux = metadata[which(metadata$FIXED_ZYGOSITY %in% c("DZ","MZ")), ]
aux = aux[-which(aux$chemo),]

aux = get_residuals(md = aux, varname = "burden_subs_passengers")
aux = get_residuals(md = aux, varname = "sig_denovo_sigA")
aux = get_residuals(md = aux, varname = "sig_denovo_sigB")
aux = get_residuals(md = aux, varname = "NOTCH1", burdencov=T)
aux = get_residuals(md = aux, varname = "TP53", burdencov=T)
aux = get_residuals(md = aux, varname = "burden_subs_passengers_blood")
aux = get_residuals(md = aux, varname = "DNMT3A_blood", burdencov=T, isblood=T)
aux = get_residuals(md = aux, varname = "TET2_blood", burdencov=T, isblood=T)

aux = get_residuals(md = aux, varname = "duplex_cov")
aux = get_residuals(md = aux, varname = "pct_selected_bases")
aux = get_residuals(md = aux, varname = "inpanel_f_eff")
aux = get_residuals(md = aux, varname = "BMI")
aux = get_residuals(md = aux, varname = "pack_years")
aux = get_residuals(md = aux, varname = "weight")
aux = get_residuals(md = aux, varname = "height")

# Creating twins object for pairs of MZ and DZ twins
twins = split(aux, f=aux$twin)
#twins = twins[names(which(unlist(sapply(twins, function(x) nrow(x)==2))))]
twins = twins[names(which(unlist(sapply(twins, function(x) nrow(x)==2 & length(unique(x$SEX))==1))))] # Same sex twin pairs with regression information from both twins
#twins = twins[names(which(unlist(sapply(twins, function(x) nrow(x)==2 & length(unique(x$SEX))==1 & sum(x$pack_years)==0))))] # Excluding smokers
```

Heritability plots for MZ, DZ and unrelated same-age pairs, for several somatic mutation variables.

```
# Plotting the absolute differences for the residuals 
if (runman) { dev.new(width=16, height=3) }
par(mfrow=c(1,8))
x = sapply(twins, function(x) unique(x$FIXED_ZYGOSITY))

y = sapply(twins, function(x) abs(diff(x$burden_subs_passengers_res)))
y1 = absdiff_sameage(aux, "burden_subs_passengers_res")
y2 = y[which(x=="DZ")]
y3 = y[which(x=="MZ")]
b = boxplot(list(y1,y2,y3), notch=T, xlab="", ylab="", outline=F, las=1, main="Mutation burden", names=c("Unrel","DZ","MZ"))
segments(x0=c(1.2,2.2,1.2), x1=c(1.8,2.8,2.8), y0=max(b$stats[5,])*c(0.8,0.8,0.9), col="cadetblue")
pvals = c(wilcox.test(y1,y2)$p.value, wilcox.test(y2,y3)$p.value, wilcox.test(y1,y3)$p.value)
text(x=c(1.2,2.2,1.2), y=max(b$stats[5,])*c(0.81,0.81,0.91), labels=sprintf("%0.2g",pvals), adj = c(0,0), cex=0.7, col="cadetblue")

y = sapply(twins, function(x) abs(diff(x$sig_denovo_sigA_res)))
y1 = absdiff_sameage(aux, "sig_denovo_sigA_res")
y2 = y[which(x=="DZ")]
y3 = y[which(x=="MZ")]
b = boxplot(list(y1,y2,y3), notch=T, xlab="", ylab="", outline=F, las=1, main="Signature A burden", names=c("Unrel","DZ","MZ"))
segments(x0=c(1.2,2.2,1.2), x1=c(1.8,2.8,2.8), y0=max(b$stats[5,])*c(0.8,0.8,0.9), col="cadetblue")
pvals = c(wilcox.test(y1,y2)$p.value, wilcox.test(y2,y3)$p.value, wilcox.test(y1,y3)$p.value)
text(x=c(1.2,2.2,1.2), y=max(b$stats[5,])*c(0.81,0.81,0.91), labels=sprintf("%0.2g",pvals), adj = c(0,0), cex=0.7, col="cadetblue")

y = sapply(twins, function(x) abs(diff(x$sig_denovo_sigB_res)))
y1 = absdiff_sameage(aux, "sig_denovo_sigB_res")
y2 = y[which(x=="DZ")]
y3 = y[which(x=="MZ")]
b = boxplot(list(y1,y2,y3), notch=T, xlab="", ylab="", outline=F, las=1, main="Signature B burden", names=c("Unrel","DZ","MZ"))
segments(x0=c(1.2,2.2,1.2), x1=c(1.8,2.8,2.8), y0=max(b$stats[5,])*c(0.8,0.8,0.9), col="cadetblue")
pvals = c(wilcox.test(y1,y2)$p.value, wilcox.test(y2,y3)$p.value, wilcox.test(y1,y3)$p.value)
text(x=c(1.2,2.2,1.2), y=max(b$stats[5,])*c(0.81,0.81,0.91), labels=sprintf("%0.2g",pvals), adj = c(0,0), cex=0.7, col="cadetblue")

y = sapply(twins, function(x) abs(diff(x$NOTCH1_res)))
y1 = absdiff_sameage(aux, "NOTCH1_res")
y2 = y[which(x=="DZ")]
y3 = y[which(x=="MZ")]
b = boxplot(list(y1,y2,y3), notch=T, xlab="", ylab="", outline=F, las=1, main="NOTCH1 fraction", names=c("Unrel","DZ","MZ"))
segments(x0=c(1.2,2.2,1.2), x1=c(1.8,2.8,2.8), y0=max(b$stats[5,])*c(0.8,0.8,0.9), col="cadetblue")
pvals = c(wilcox.test(y1,y2)$p.value, wilcox.test(y2,y3)$p.value, wilcox.test(y1,y3)$p.value)
text(x=c(1.2,2.2,1.2), y=max(b$stats[5,])*c(0.81,0.81,0.91), labels=sprintf("%0.2g",pvals), adj = c(0,0), cex=0.7, col="cadetblue")

y = sapply(twins, function(x) abs(diff(x$TP53_res)))
y1 = absdiff_sameage(aux, "TP53_res")
y2 = y[which(x=="DZ")]
y3 = y[which(x=="MZ")]
b = boxplot(list(y1,y2,y3), notch=T, xlab="", ylab="", outline=F, las=1, main="TP53 fraction", names=c("Unrel","DZ","MZ"))
segments(x0=c(1.2,2.2,1.2), x1=c(1.8,2.8,2.8), y0=max(b$stats[5,])*c(0.8,0.8,0.9), col="cadetblue")
pvals = c(wilcox.test(y1,y2)$p.value, wilcox.test(y2,y3)$p.value, wilcox.test(y1,y3)$p.value)
text(x=c(1.2,2.2,1.2), y=max(b$stats[5,])*c(0.81,0.81,0.91), labels=sprintf("%0.2g",pvals), adj = c(0,0), cex=0.7, col="cadetblue")

y = sapply(twins, function(x) abs(diff(x$burden_subs_passengers_blood_res)))
y1 = absdiff_sameage(aux, "burden_subs_passengers_blood_res")
y2 = y[which(x=="DZ")]
y3 = y[which(x=="MZ")]
b = boxplot(list(y1,y2,y3), notch=T, xlab="", ylab="", outline=F, las=1, main="Mutation burden blood", names=c("Unrel","DZ","MZ"))
segments(x0=c(1.2,2.2,1.2), x1=c(1.8,2.8,2.8), y0=max(b$stats[5,])*c(0.8,0.8,0.9), col="cadetblue")
pvals = c(wilcox.test(y1,y2)$p.value, wilcox.test(y2,y3)$p.value, wilcox.test(y1,y3)$p.value)
text(x=c(1.2,2.2,1.2), y=max(b$stats[5,])*c(0.81,0.81,0.91), labels=sprintf("%0.2g",pvals), adj = c(0,0), cex=0.7, col="cadetblue")

y = sapply(twins, function(x) abs(diff(x$DNMT3A_blood_res)))
y1 = absdiff_sameage(aux, "DNMT3A_blood_res")
y2 = y[which(x=="DZ")]
y3 = y[which(x=="MZ")]
b = boxplot(list(y1,y2,y3), notch=T, xlab="", ylab="", outline=F, las=1, main="DNMT3A blood fraction", names=c("Unrel","DZ","MZ"))
segments(x0=c(1.2,2.2,1.2), x1=c(1.8,2.8,2.8), y0=max(b$stats[5,])*c(0.8,0.8,0.9), col="cadetblue")
pvals = c(wilcox.test(y1,y2)$p.value, wilcox.test(y2,y3)$p.value, wilcox.test(y1,y3)$p.value)
text(x=c(1.2,2.2,1.2), y=max(b$stats[5,])*c(0.81,0.81,0.91), labels=sprintf("%0.2g",pvals), adj = c(0,0), cex=0.7, col="cadetblue")

y = sapply(twins, function(x) abs(diff(x$TET2_blood_res)))
y1 = absdiff_sameage(aux, "TET2_blood_res")
y2 = y[which(x=="DZ")]
y3 = y[which(x=="MZ")]
b = boxplot(list(y1,y2,y3), notch=T, xlab="", ylab="", outline=F, las=1, main="TET2 blood fraction", names=c("Unrel","DZ","MZ"))
segments(x0=c(1.2,2.2,1.2), x1=c(1.8,2.8,2.8), y0=max(b$stats[5,])*c(0.8,0.8,0.9), col="cadetblue")
pvals = c(wilcox.test(y1,y2)$p.value, wilcox.test(y2,y3)$p.value, wilcox.test(y1,y3)$p.value)
text(x=c(1.2,2.2,1.2), y=max(b$stats[5,])*c(0.81,0.81,0.91), labels=sprintf("%0.2g",pvals), adj = c(0,0), cex=0.7, col="cadetblue")
```

```
if (runman) { dev.copy(pdf, file = "Heritability_using_glm_residuals.pdf", width=16, height=3); dev.off() }
```

Repeating the plots for positive control variables and some technical variables.

```
if (runman) { dev.new(width=6, height=4) }
par(mfrow=c(1,3))
x = sapply(twins, function(x) unique(x$FIXED_ZYGOSITY))

y = sapply(twins, function(x) abs(diff(x$BMI_res)))
y1 = absdiff_sameage(aux, "BMI_res")
y2 = y[which(x=="DZ")]
y3 = y[which(x=="MZ")]
b = boxplot(list(y1,y2,y3), notch=T, xlab="", ylab="", outline=F, las=1, main="BMI_res", names=c("Unrel","DZ","MZ"))
segments(x0=c(1.2,2.2,1.2), x1=c(1.8,2.8,2.8), y0=max(b$stats[5,])*c(0.8,0.8,0.9), col="cadetblue")
pvals = c(wilcox.test(y1,y2)$p.value, wilcox.test(y2,y3)$p.value, wilcox.test(y1,y3)$p.value)
text(x=c(1.2,2.2,1.2), y=max(b$stats[5,])*c(0.81,0.81,0.91), labels=sprintf("%0.2g",pvals), adj = c(0,0), cex=0.7, col="cadetblue")

y = sapply(twins, function(x) abs(diff(x$height_res)))
y1 = absdiff_sameage(aux, "height_res")
y2 = y[which(x=="DZ")]
y3 = y[which(x=="MZ")]
b = boxplot(list(y1,y2,y3), notch=T, xlab="", ylab="", outline=F, las=1, main="height_res", names=c("Unrel","DZ","MZ"))
segments(x0=c(1.2,2.2,1.2), x1=c(1.8,2.8,2.8), y0=max(b$stats[5,])*c(0.8,0.8,0.9), col="cadetblue")
pvals = c(wilcox.test(y1,y2)$p.value, wilcox.test(y2,y3)$p.value, wilcox.test(y1,y3)$p.value)
text(x=c(1.2,2.2,1.2), y=max(b$stats[5,])*c(0.81,0.81,0.91), labels=sprintf("%0.2g",pvals), adj = c(0,0), cex=0.7, col="cadetblue")

y = sapply(twins, function(x) abs(diff(x$weight_res)))
y1 = absdiff_sameage(aux, "weight_res")
y2 = y[which(x=="DZ")]
y3 = y[which(x=="MZ")]
b = boxplot(list(y1,y2,y3), notch=T, xlab="", ylab="", outline=F, las=1, main="weight_res", names=c("Unrel","DZ","MZ"))
segments(x0=c(1.2,2.2,1.2), x1=c(1.8,2.8,2.8), y0=max(b$stats[5,])*c(0.8,0.8,0.9), col="cadetblue")
pvals = c(wilcox.test(y1,y2)$p.value, wilcox.test(y2,y3)$p.value, wilcox.test(y1,y3)$p.value)
text(x=c(1.2,2.2,1.2), y=max(b$stats[5,])*c(0.81,0.81,0.91), labels=sprintf("%0.2g",pvals), adj = c(0,0), cex=0.7, col="cadetblue")
```

```
if (runman) { dev.copy(pdf, file = "Heritability_using_glm_residuals_dummy.pdf", width=6, height=4); dev.off() }
```

Using the function above we can add residuals for major driver genes to the metadata table.

```
genes2test = genes2test_ref
for (j in 1:length(genes2test)) {
  aux = get_residuals(md = aux, varname = genes2test[j], burdencov=T)
}

# Adding the columns of interest to the full metadata data frame
vars2save = c(genes2test,"burden_subs_passengers","sig_denovo_sigA","sig_denovo_sigB","BMI","weight","height")
aux = aux[,c("donor",paste(vars2save,"_res",sep=""))]
metadata = metadata[,c("donor",setdiff(colnames(metadata),colnames(aux)))] # We remove previous versions of the columns that we want to add (not necessary if running the code from the start)
metadata = merge(metadata, unique(aux[,c("donor",setdiff(colnames(aux),colnames(metadata)))]), by="donor", all.x=TRUE)
write.table(metadata, file = new_metadata_file, row.names=F, col.names=T, sep="\t", quote=F)

# We can also use the residuals to generate a pairwise correlation matrix between driver genes to see which genes co-vary after removing confounding variables
resmat = as.matrix(aux[,paste(genes2test,"_res",sep="")])
resmat = resmat[!is.na(rowSums(resmat)),]
paircorr_drivers_r = paircorr_drivers_pval = array(NA, dim=rep(ncol(resmat),2), dimnames = list(colnames(resmat),colnames(resmat))) # Initialising
for (j in 1:ncol(resmat)) {
  for (h in 1:ncol(resmat)) {
    corrdriv = cor.test(resmat[,j],resmat[,h],method="spearman")
    paircorr_drivers_r[j,h] = corrdriv$estimate
    paircorr_drivers_pval[j,h] = corrdriv$p.value
  }
}
paircorr_drivers_qval = array(p.adjust(paircorr_drivers_pval, method = "BH"), dim=dim(paircorr_drivers_pval))

write.table(paircorr_drivers_r, file = "Drivers_correlation_matrix_spearman.txt", row.names=T, col.names=T, sep="\t", quote=F)
write.table(paircorr_drivers_pval, file = "Drivers_correlation_matrix_pvals.txt", row.names=T, col.names=T, sep="\t", quote=F)
```

We can then study co-variation in driver frequency across individuals using the residuals (akin to a partial correlation analysis).

```
logp = -log10(paircorr_drivers_pval)
maxval = 18 # Maximum value for -log(pval) [=30 caps p-values to a minimum of 1e-25]
logp[logp>maxval] = maxval
diag(logp) = NA
colnames(logp) = rownames(logp) = gsub("_res","",colnames(logp)) # Removing the res suffix
logp[which(paircorr_drivers_r<0)] = -logp[which(paircorr_drivers_r<0)]
blueredpal = colorRampPalette(c("steelblue4","steelblue2","white","peachpuff2","indianred4"))(n = 101)
if (runman) { dev.new(width=8, height=8) }
heatmap.2(x=logp, col=blueredpal, density.info="none", trace="none", margins = c(6,6), scale = "none")
```

```
if (runman) { dev.copy(pdf, file = "Drivers_correlation_heatmap_logp.pdf", width=5, height=5); dev.off() }
```

## 4. Within-gene selection analyses

The high density of mutations in this dataset allow us to quantify selection at specific sites or groups of sites within driver genes, including in non-coding regions with sufficient duplex coverage. To enable this, in this study we introduce a new function in the dNdScv package called *withingenednds*. Below we include code to generate some of the summary plots in Figure 3 of the manuscript.

Plotting the *withingenednds* q-values across all the features and genes tested.

```
# Loading the withingenednds output files in a single table
wdnds = NULL
for (j in 1:length(targetgenes)) {
  w = read.table(sprintf("withingenednds_files/%s_dnds_nbfix.tsv",targetgenes[j]), header=1, sep="\t", stringsAsFactors=F)
  w = cbind(data.frame(gene=rep(targetgenes[j],nrow(w))),w)
  wdnds = rbind(wdnds, w)
}
write.table(wdnds, file="withingenednds_nbfix_results.tsv", col.names=T, row.names = F, sep = "\t", quote = F) # Saving the concatenated table

# Calculating q-values
feat2plot = c("wmis","wnon","wspl","wexfl","winfl","wstartloss","wstoploss","wcorepromoter","wdownstreampolyA") # Features to plot
wdnds$qval.lrt = NA
for (j in 1:length(feat2plot)) {
  rowind = (wdnds$name==feat2plot[j] & !is.na(wdnds$pval.lrt)) # vector positions for the p-values to correct
  wdnds$qval.lrt[rowind] = p.adjust(wdnds$pval.lrt[rowind], method="BH")
}

# Function to generate the q-value plots using a concatenated withingenednds output table (based on the code from PMID:29056346, Fig 2)

drawlist = function(w, qcuts=c(1e-5,1e-3,0.01), qcutscol = c("firebrick3","orange","lightgoldenrod"), valrange=c(0.1,100), ticks=c(0.1,0.2,0.5,1,2,5,10,20,50,100), xstart=0, plotaxis=F, greygenes = NULL, plotname = NULL) {
    
  if (length(qcuts)!=length(qcutscol)) {
    error("Please ensure that qcuts and qcutscol have the same length")
  }
  
  if (is.null(plotname)) {
    wname = unique(w$name)
  } else {
    wname = plotname
  }
  # Excluding non-significant genes
  w = w[which(w$qval.lrt<max(qcuts)), ]
  
  # Function to map a value into the plotting range
  maptorange = function(x, xmin, xmax) {
    sapply(x, function(v) (pmin(pmax(v,xmin),xmax)-xmin) / (xmax-xmin))
  }
  
  # y-axis position for each gene in the range of plotting values
  w$mlelog = log10(w$mle) # Using log10(dN/dS) as the plotting metric
  miny = log10(min(valrange))
  maxy = log10(max(valrange))
  w$y = maptorange(w$mlelog, xmin=miny, xmax=maxy)
  w = w[order(w$y,decreasing = T), ]
  
  # Position of the gene name (iterative optimisation to avoid overlaps)
  w$yname = w$y
  mindist = (maxy-miny)/50 # Minimum distance between genes for plotting
  while( any((-diff(w$yname))<mindist) ) {
    ydiff = as.numeric(-diff(w$yname))
    runs = rle(ydiff<mindist)
    aux = which(ydiff<mindist)[1]
    l = runs$lengths[which(runs$values)[1]]
    cl = seq(aux,aux+l)
    # New suggested values for the cluster chosen
    centr = median(w$yname[cl])
    w$yname[cl] = seq(mindist*(length(cl)-1)+0.0001, 0, length.out=length(cl))
    w$yname[cl] = w$yname[cl] - median(w$yname[cl]) + centr
  }
  
  # Shifting gene names upwards
  #w$yname = w$yname - min(w$yname)
  
  # Significance class (dot colour)
  w$cut = cut(w$qval.lrt, breaks = c(-Inf,sort(qcuts)))
  w$classcol = setNames(qcutscol,levels(w$cut))[w$cut]
  
  # Plot
  tck = 0.08/dev.size("in")[1]
  tck2 = 0.05/dev.size("in")[1]
  aux = maptorange(log10(ticks), xmin=miny, xmax=maxy)
  if (plotaxis) { axis(2, at=aux, labels=ticks, las=1, tck=-0.02) }
  rect(xstart, 0, xstart+tck, 1, col="slategray3", border=NA)
  segments(xstart, aux, xstart+tck, aux, lwd=2, col="white")
  
  segments(xstart, w$y, xstart+tck, w$y)
  segments(xstart+tck, w$y, xstart+tck+tck2, w$yname)
  segments(xstart+tck+tck2, w$yname, xstart+tck+tck2+tck2, w$yname)
  
  w$genecol = "black"
  if (length(greygenes>0)) {
    w$genecol[w$gene %in% greygenes] = "grey60"
  }
  
  text(x = xstart+tck+tck+tck2, y = w$yname, labels = w$gene, cex=0.6, pos=4, offset=0.3, font=3, col=w$genecol)
  points(x = rep(xstart+tck+tck+tck2+tck2/2,nrow(w)), y=w$yname, pch=20, col=w$classcol, cex=0.6)
  text(x = xstart, y = 1.02, labels=sprintf("%s",wname), cex=0.6, pos=4, offset=0)
}

notdrivers = setdiff(targetgenes, drivers)

if (runman) { dev.new(width=6,height=4.5) }
par(xpd=NA); plot.new()
drawlist(wdnds[which(wdnds$name=="wmis" & wdnds$mle>1),], valrange=c(1,100), ticks=c(1,2,5,10,20,50,100), greygenes=notdrivers, xstart=0, plotaxis=T, plotname="Missense")
drawlist(wdnds[which(wdnds$name=="wnon" & wdnds$mle>1),], valrange=c(1,100), ticks=c(1,2,5,10,20,50,100), greygenes=notdrivers, xstart=0.2, plotname="Nonsense")
drawlist(wdnds[which(wdnds$name=="wspl" & wdnds$mle>1),], valrange=c(1,100), ticks=c(1,2,5,10,20,50,100), greygenes=notdrivers, xstart=0.4, plotname="Essential splice")
drawlist(wdnds[which(wdnds$name=="wcorepromoter" & wdnds$mle>1),], valrange=c(1,100), ticks=c(1,2,5,10,20,50,100), greygenes=notdrivers, xstart=0.6, plotname="Core promoter")
drawlist(wdnds[which(wdnds$name=="winfl" & wdnds$mle>1),], valrange=c(1,100), ticks=c(1,2,5,10,20,50,100), greygenes=notdrivers, xstart=0.8, plotname="Intron flank")
drawlist(wdnds[which(wdnds$name=="wstoploss" & wdnds$mle>1),], valrange=c(1,100), ticks=c(1,2,5,10,20,50,100), greygenes=notdrivers, xstart=1, plotname="Stop loss")
```

```
if (runman) { dev.copy(pdf,"withingenednds_signifgenes.pdf",width=6,height=4.5); dev.off() }
```

Plotting the number of mutations in TwinsUK vs COSMIC (all whole-genome or whole-exome sequencing studies or restricted to squamous cell carcinomas).

```
# Loading COSMIC files
cosmic = read.table(cosmic_mutation_file, header=1, sep="\t", stringsAsFactors=F)
cosmic_samples = read.table(cosmic_samples_file, header=1, sep="\t", stringsAsFactors = F, quote="", fill = T)
wgswes_samples = cosmic_samples$COSMIC_SAMPLE_ID[which(cosmic_samples$WHOLE_GENOME_SCREEN=="y" | cosmic_samples$WHOLE_EXOME_SCREEN=="y")] # All WGS/WES samples in the COSMIC file
scc_samples = cosmic_samples$COSMIC_SAMPLE_ID[which((cosmic_samples$WHOLE_GENOME_SCREEN=="y" | cosmic_samples$WHOLE_EXOME_SCREEN=="y") & cosmic_samples$HISTOLOGY_SUBTYPE_1=="squamous_cell_carcinoma")]
# Counting the number of mutations per gene
genes2plot = genes2test_ref
n1 = table(mutations$gene)
n2 = table(cosmic$gene[cosmic$sampleID %in% wgswes_samples])
n3 = table(cosmic$gene[cosmic$sampleID %in% scc_samples])
nummuts_gene = data.frame(gene=genes2plot, TwinsUK=as.numeric(n1[genes2plot]), cosmic_all=as.numeric(n2[genes2plot]), cosmic_scc=as.numeric(n3[genes2plot]))
print(nummuts_gene)
```

```
##       gene TwinsUK cosmic_all cosmic_scc
## 1   NOTCH1   20734       2433        488
## 2     TP53    8079      12219       2614
## 3     FAT1    8765       2762        540
## 4    CHEK2    2773        477         70
## 5    PPM1D    2499        297         35
## 6    ASXL1    3089        927         89
## 7   NOTCH2    5010       1305        180
## 8  ZFP36L2    2182        556         55
## 9     RAC1    1389        203         34
## 10  BCORL1    2257       1029         96
```

```
# Summary barplot
if (runman) { dev.new(width=6,height=6) }

colvec = c("cadetblue","grey30","grey70")
barplot(t(as.matrix(nummuts_gene[,-1])), beside=T, las=2, col=colvec, border=NA, names.arg = nummuts_gene$gene,
        ylab="Number of mutations per gene")
legend(x=6, y=max(nummuts_gene[,-1]), legend = c("This study",sprintf("COSMIC (n=%0.0f WGS+WES)",length(wgswes_samples)),sprintf("COSMIC (n=%0.0f SCC)",length(scc_samples))), pch=15, col=colvec, cex=0.7, box.col = NA)
```

```
if (runman) { dev.copy(pdf,"Number_mutations_TwinsUK_vs_COSMIC.pdf",width=6,height=6); dev.off() }
```

Evaluating the relationship between site-dN/dS values and ClinVar annotations for 3 commonly mutated genes.

```
if (runman) { dev.new(width=7,height=3) }
par(mfrow=c(1,length(clinvar_files)))
impacts2plot = c("Missense","Nonsense","Essential_Splice")

for (j in 1:length(clinvar_files)) {
  gene = names(clinvar_files)[j]
  w = read.table(clinvar_files[[j]], header=1, sep="\t", stringsAsFactors=F)
  w$clinvar_class = NA
  w$clinvar_class[w$clinvar %in% c("Benign")] = "1. Benign"
  w$clinvar_class[w$clinvar %in% c("Uncertain_significance")] = "2. Uncertain"
  w$clinvar_class[w$clinvar %in% c("Pathogenic")] = "3. Pathogenic"
  w = w[which(!is.na(w$clinvar_class) & w$impact %in% impacts2plot), ]
  w$w = pmax(pmin(w$obs/w$rnorm,200),0.1)
  w$log10w = log10(w$w)
  
  # Annotating sites significant by sitednds
  sigsite = hotspots_allsites$recursites[which(hotspots_allsites$recursites$gene==gene & hotspots_allsites$recursites$qval<0.20),]
  sigsite$mstr = paste(sigsite$chr, sigsite$pos, sigsite$mut, sep=":")
  w$mstr = paste(w$chr, w$pos, w$mut, sep=":")
  w$qval = setNames(sigsite$qval, sigsite$mstr)[w$mstr]

  # Boxplot
  b = boxplot(w$log10w~w$clinvar_class, notch=F, ylab="log10 (site-dN/dS)", xlab="", outline=F, las=2, main=gene, names=c("Benign","Uncertain","Pathogenic"))
  w$x = jitter(setNames(1:3,c("1. Benign","2. Uncertain","3. Pathogenic"))[w$clinvar_class])
  points(w$x[-which(w$qval<0.15)], w$log10w[-which(w$qval<0.15)], col="grey30", cex=0.7)
  points(w$x[which(w$qval<0.15)], w$log10w[which(w$qval<0.15)], col="indianred", cex=0.8)
  
  # Outputting a message on the number of dN/dS-significant sites in the "Uncertain significance" class
  message(sprintf("%s: %0.0f mutations annotated as Uncertain-significance in ClinVar are significant by sitednds", gene, length(which(w$qval[w$clinvar=="Uncertain_significance"]<0.2))))
  
  # Saving a file with the ClinVar results
  write.table(w[which(w$qval<0.2),], file = sprintf("ClinVar_sitednds_signifsites_%s.tsv",gene), row.names=F, col.names=T, sep="\t", quote=F)
}
```

```
## NOTCH1: 35 mutations annotated as Uncertain-significance in ClinVar are significant by sitednds
```

```
## TP53: 86 mutations annotated as Uncertain-significance in ClinVar are significant by sitednds
```

```
## PPM1D: 5 mutations annotated as Uncertain-significance in ClinVar are significant by sitednds
```

```
if (runman) { dev.copy(pdf,"ClinVar_and_sitedNdS.pdf",width=7,height=3); dev.off() }
```

## 5. Nanoseq error rates and summary plots

Plots showing the error rates of duplex sequencing and Nanoseq protocols, including the sonication and enzymatic versions, when applied to cord blood and a set of FFPE samples.

```
rates = read.table("Error_rates_new_Nanoseq_protocols.txt", header=1, sep="\t", stringsAsFactors=F)
rates = rates[order(rates$Type, rates$Plot_order),]
embryonic_correction_factor = 1.32 # Estimated correction factor for missing embryonic variants, as described in Abascal et al. 2021 (PMID:33911282)

if (runman) { dev.new(width=8, height=4) }
par(mfrow=c(1,2))

b = barplot(rates$Burden[rates$Type=="cordblood"], las=2, ylab="Mutation burden", names.arg = rates$Public_name[rates$Type=="cordblood"], border = NA,
            ylim = c(0, max(rates$UCI[rates$Type=="cordblood"])), col = c("wheat4","wheat4","wheat3","wheat3","cadetblue4","cadetblue4","cadetblue3","cadetblue3"))
segments(x0=b, y0=rates$LCI[rates$Type=="cordblood"], y1=rates$UCI[rates$Type=="cordblood"])
abline(h=109/6e9/embryonic_correction_factor, col="grey40", lty=1)
abline(h=c(95,125)/6e9/embryonic_correction_factor, col="grey40", lty=2)

inds = (rates$Type=="ffpe" & !(rates$Public_name %in% c("NanoEZ_Control","NanoSH_Control")))
b = barplot(rates$Burden[inds], las=2, ylab="Mutation burden", names.arg = rates$Public_name[inds], ylim = c(0, max(rates$UCI[inds])), border = NA,
            col=c("wheat4","wheat4","wheat3","wheat3","cadetblue4","cadetblue4","cadetblue3","cadetblue3"))
segments(x0=b, y0=rates$LCI[inds], y1=rates$UCI[inds])
abline(h=rates$Burden[rates$Type=="ffpe" & rates$Public_name %in% c("NanoEZ_Control","NanoSH_Control")], col="grey40")
```

```
if (runman) { dev.copy(pdf, "Fig1_Nanoseq_error_rates.pdf", width=7, height=4); dev.off() }
```

Generating a few plots to summarise the buccal dataset:

1. Histogram of duplex depth.
2. Adjusted BAM vaf for all the mutations called
3. Ranked dot plot for the number of SNVs, coding SNVs, indels and DNVs per sample.
4. Scatter plot SNV burden vs age.

```
if (runman) { dev.new(width=9, height=6) }
par(mfrow = c(2,3))

# a. Histogram of duplex depth
hist(metadata$duplex_cov, breaks = seq(0,max(metadata$duplex_cov+100), by=100), las=2, ylab="Number donors", xlab="Mean duplex coverage", main="")
abline(v=mean(metadata$duplex_cov), col="cadetblue", lwd=2)
print(mean(metadata$duplex_cov))
```

```
## [1] 665.2666
```

```
# b. Adjusted BAM vaf for all the mutations called
if (1) { # Standard VAF histogram
  
  minbamdepth = 2000 # We will only plot the VAF of mutations with this minimum depth
  isdeep = (mutations$bam_cov-mutations$duplex_cov) >= minbamdepth
  mutations$bam_vaf_corr = (mutations$bam_mut-mutations$times_called)/(mutations$bam_cov-mutations$duplex_cov)
  aux = pmax(1e-5,mutations$bam_vaf_corr) # Replacing 0s with a minimum value for representation purposes
  hist(log10(aux[isdeep]), las=1, main="", xlab="Unbiased VAF (log10)", col="grey60", border=NA)
  message(sprintf("Fraction of unbiased VAFs (for sites with >%0.0fx): <1%% %0.3g, <0.1%% %0.3g", minbamdepth, 
                  mean(mutations$bam_vaf_corr[isdeep]<0.01), mean(mutations$bam_vaf_corr[isdeep]<0.001)))
  
} else {
  
  # Here we plot the unbiased bam VAF for mutations with at least 1 mutant read
  minbamdepth = 2000 # We will only plot the VAF of mutations with this minimum depth
  isdeep = (mutations$bam_cov-mutations$duplex_cov) >= minbamdepth
  bam_vaf_corr_mutationsobserved = ((mutations$bam_mut-mutations$times_called)/(mutations$bam_cov-mutations$duplex_cov))
  bam_vaf_corr_notobserved = (1/(mutations$bam_cov-mutations$duplex_cov)) # Upper bound unbiased VAF estimate for sites without mutant reads not used for calling
  zeromut = (mutations$bam_mut-mutations$times_called) <= 0 # Sites with no mutant reads observed outside the duplex reads
  
  # Calculating the frequencies
  h1 = hist(log10(bam_vaf_corr_mutationsobserved[isdeep & !zeromut]), plot=F, breaks=seq(-4.625,-0.375,by=0.25))
  h2 = hist(log10(bam_vaf_corr_notobserved[isdeep & zeromut]), plot=F, breaks=seq(-4.625,-0.375,by=0.25))
  barplot(rbind(h2$counts,h1$counts), col=c("grey51","grey71"), border=NA, names.arg=h1$mids, las=2, main="", xlab="Unbiased VAF (log10)", ylab=sprintf("Number of mutations (>%0.0fx)",minbamdepth))
  legend(x=8, y=1e5, pch=15, col=c("grey51","grey71"), legend=c("Mutations only seen in duplex reads", "Mutations seen outside duplex read"), box.col = NA)
}
```

```
## Fraction of unbiased VAFs (for sites with >2000x): <1% 0.997, <0.1% 0.938
```

```
# c. Ranked dot plot for the number of SNVs, coding SNVs, indels and DNVs per sample
v = sort(table(mutations$sampleID))
nmuts = data.frame(sampleID=names(v), allmuts=as.numeric(v))
nmuts$cod_snv = table(mutations$sampleID[mutations$impact %in% c("Synonymous","Missense","Nonsense","Essential_Splice","Stop_loss")])[nmuts$sampleID] # Counting coding SNVs per sample
nmuts$cod_snv[is.na(nmuts$cod_snv)] = 0
nmuts$cod_ind = table(mutations$sampleID[mutations$impact=="no-SNV" & nchar(mutations$ref)!=nchar(mutations$mut)])[nmuts$sampleID] # Counting coding indels per sample
nmuts$cod_ind[is.na(nmuts$cod_ind)] = 0
nmuts$cod_dnv = table(mutations$sampleID[mutations$impact=="no-SNV" & nchar(mutations$ref)==2 & nchar(mutations$mut)==2])[nmuts$sampleID] # Counting coding DNVs per sample
nmuts$cod_dnv[is.na(nmuts$cod_dnv)] = 0
nmuts$age = setNames(metadata$AGE, metadata$pd)[nmuts$sampleID]

plot(1:nrow(nmuts), nmuts$allmuts, pch=20, col="black", las=2, xlab="Donors (ranked by total mutations)", ylab="Mutations per donor", log="y", cex=0.7, ylim=c(3,max(nmuts$allmuts)))
points(1:nrow(nmuts), nmuts$cod_snv, col="cadetblue", pch=20, cex=0.7)
points(1:nrow(nmuts), nmuts$cod_ind, col="plum4", pch=20, cex=0.7)
#points(1:nrow(nmuts), nmuts$cod_dnv, col="mistyrose3", pch=20, cex=0.7)
legend(x=0, y=max(nmuts$allmuts), legend = c("All mutations","Coding SNVs","Coding indels"), pch=20, col=c("black","cadetblue","plum4"), cex=0.7, box.col = NA)

# d. Ranked dot plot
genes2plot = c("NOTCH1","FAT1","TP53","NOTCH2")
ginds = which(colnames(nmuts_ns) %in% genes2plot)
#selected_samples = metadata$pd[metadata$duplex_cov>=1000 & metadata$AGE>=65 & metadata$AGE<=85]
selected_samples = intersect(metadata$pd,rownames(nmuts_ns)) # All samples (for the dataset description)
colvec = c("hotpink4","darkorchid3","chocolate","darkslategray3")
plot(1:length(selected_samples), sort(nmuts_ns[selected_samples,ginds[1]]), col=colvec[ginds[1]], xlab="Donors (ranked per gene)", ylab="Non-synonymous per donor", log="y", pch=20, cex=0.7, las=2, ylim=c(1,max(nmuts_ns[selected_samples,ginds])))
```

```
## Warning in xy.coords(x, y, xlabel, ylabel, log): 27 y values <= 0 omitted from
## logarithmic plot
```

```
for (j in 2:length(ginds)) {
  points(1:length(selected_samples), sort(nmuts_ns[selected_samples,ginds[j]]), col=colvec[ginds[j]], pch=20, cex=0.7)
}
legend(x=1, y=max(max(nmuts_ns[selected_samples,ginds])), legend=colnames(nmuts_ns)[ginds], pch=16, border=NA, box.col=NA, col=colvec, cex=0.7, bg="transparent")

# e. Scatter plot SNV burden vs age
plot(metadata$AGE, metadata$burden_subs_passengers*genome_length, xlab="Donor age", ylab="SNV burden (per diploid cell)", las=1, cex=0.6)
model = glm((metadata$burden_subs_passengers*genome_length)~metadata$AGE)
abline(model, col="darkslategray4")
cimod = confint(model)
```

```
## Waiting for profiling to be done...
```

```
leg = sprintf("Intercept: %0.1f (%0.1f-%0.1f)\nSlope: %0.1f (%0.1f-%0.1f)",
              model$coefficients[1],cimod[1,1],cimod[1,2], model$coefficients[2],cimod[2,1],cimod[2,2])
text(x=min(metadata$AGE), y=max(metadata$burden_subs_passengers,na.rm=T)*genome_length, labels=leg, adj=c(0,1))

# f. Scatter plot indel burden vs age
plot(metadata$AGE, metadata$burden_indels_passengers*genome_length, xlab="Donor age", ylab="Indel burden (per diploid cell)", las=1, cex=0.6)
model = glm((metadata$burden_indels_passengers*genome_length)~metadata$AGE)
abline(model, col="darkslategray4")
cimod = confint(model)
```

```
## Waiting for profiling to be done...
```

```
leg = sprintf("Intercept: %0.1f (%0.1f-%0.1f)\nSlope: %0.1f (%0.1f-%0.1f)",
              model$coefficients[1],cimod[1,1],cimod[1,2], model$coefficients[2],cimod[2,1],cimod[2,2])
text(x=min(metadata$AGE), y=max(metadata$burden_indels_passengers,na.rm=T)*genome_length, labels=leg, adj=c(0,1))
```

```
if (runman) { dev.copy(pdf,"Dataset_description.pdf",width=9,height=6); dev.off() }
```

Analogous plots for blood:

1. Histogram of duplex depth.
2. Adjusted BAM vaf for all the mutations called.
3. Ranked dot plot for the number of SNVs, coding SNVs, indels and DNVs per sample.

```
if (runman) { dev.new(width=9, height=4) }
par(mfrow = c(1,3))

# a. Histogram of duplex depth
hist(bb$mean_duplex_cov, breaks = seq(0,max(bb$mean_duplex_cov+100), by=100), las=2, ylab="Number donors", xlab="Mean duplex coverage", main="")
abline(v=mean(bb$mean_duplex_cov), col="cadetblue", lwd=2)
print(mean(bb$mean_duplex_cov))
```

```
## [1] 676.4074
```

```
# b. Adjusted BAM vaf for all the mutations called
bloodmuts$bam_vaf_corr = (bloodmuts$bam_mut-bloodmuts$times_called)/(bloodmuts$bam_cov-bloodmuts$duplex_cov)
aux = pmax(1e-5,bloodmuts$bam_vaf_corr) # Replacing 0s with a minimum value for representation purposes
hist(log10(aux), las=1, main="", xlab="Unbiased VAF (log10)")

# c. Ranked dot plot for the number of SNVs, coding SNVs, indels and DNVs per sample
v = sort(table(bloodmuts$sampleID))
nmuts = data.frame(sampleID=names(v), allmuts=as.numeric(v))
nmuts$cod_snv = table(bloodmuts$sampleID[bloodmuts$impact %in% c("Synonymous","Missense","Nonsense","Essential_Splice","Stop_loss")])[nmuts$sampleID] # Counting coding SNVs per sample
nmuts$cod_snv[is.na(nmuts$cod_snv)] = 0
nmuts$cod_ind = table(bloodmuts$sampleID[bloodmuts$impact=="no-SNV" & nchar(bloodmuts$ref)!=nchar(bloodmuts$mut)])[nmuts$sampleID] # Counting coding indels per sample
nmuts$cod_ind[is.na(nmuts$cod_ind)] = 0
nmuts$cod_dnv = table(bloodmuts$sampleID[bloodmuts$impact=="no-SNV" & nchar(bloodmuts$ref)==2 & nchar(bloodmuts$mut)==2])[nmuts$sampleID] # Counting coding DNVs per sample
nmuts$cod_dnv[is.na(nmuts$cod_dnv)] = 0
nmuts$age = setNames(bb$AGE, bb$sample)[nmuts$sampleID]

plot(1:nrow(nmuts), nmuts$allmuts, pch=20, col="black", las=2, xlab="Donors (ranked)", ylab="Mutations per donor", log="y", cex=0.7, ylim=c(3,max(nmuts$allmuts)))
points(1:nrow(nmuts), nmuts$cod_snv, col="cadetblue", pch=20, cex=0.7)
points(1:nrow(nmuts), nmuts$cod_ind, col="plum4", pch=20, cex=0.7)
#points(1:nrow(nmuts), nmuts$cod_dnv, col="mistyrose3", pch=20, cex=0.7)
legend(x=0, y=max(nmuts$allmuts), legend = c("All mutations","Coding SNVs","Coding indels"), pch=20, col=c("black","cadetblue","plum4"), cex=0.7, box.col = NA)
```

```
if (runman) { dev.copy(pdf,"Dataset_description_blood.pdf",width=9,height=3); dev.off() }
```

We can test whether the mutation burden in blood and buccal swabs shows some correlation. We can do this before and after regressing out the effect of age and other confounders (akin to a partial correlation analysis).

```
if (runman) { dev.new(width=7, height=4) }
par(mfrow=c(1,2))

plot(metadata$burden_subs_passengers, metadata$burden_subs_passengers_blood, las=1, xlab="Burden buccal swabs (passenger SNVs)", ylab="Burden blood (passenger SNVs)", cex=0.7, cex.lab=0.8, cex.axis=0.8)
corr = cor.test(metadata$burden_subs_passengers, metadata$burden_subs_passengers_blood)
text(x=max(metadata$burden_subs_passengers,na.rm=T), y=max(metadata$burden_subs_passengers_blood,na.rm=T), adj=c(1,1), cex=0.8,
     label = sprintf("Pearson r=%0.2f\nP=%0.3g",corr$estimate,corr$p.value))

metadata = get_residuals(md = metadata, varname = "burden_subs_passengers")
metadata = get_residuals(md = metadata, varname = "burden_subs_passengers_blood")
plot(metadata$burden_subs_passengers_res, metadata$burden_subs_passengers_blood_res, las=1, xlab="Residuals burden buccal swabs (passenger SNVs)", ylab="Residuals burden blood (passenger SNVs)", cex=0.7, cex.lab=0.8, cex.axis=0.8)
corr = cor.test(metadata$burden_subs_passengers_res, metadata$burden_subs_passengers_blood_res)
text(x=max(metadata$burden_subs_passengers_res,na.rm=T), y=max(metadata$burden_subs_passengers_blood_res,na.rm=T), adj=c(1,1), cex=0.8,
     label = sprintf("Pearson r=%0.2f\nP=%0.3g",corr$estimate,corr$p.value))
```

```
if (runman) { dev.copy(pdf,"Burden_correlation_buccal_vs_blood.pdf",width=7,height=4); dev.off() }
```

Driver plot for the blood data.

```
blood_sample_selection = read.table(blooddonorcriteria_file, header=1, sep="\t", stringsAsFactors=F)
unbiased_bloods = rownames(blood_sample_selection)[rowSums(blood_sample_selection[,c("mfDZpair","high_burden","low_burden","high_driver_frac","low_driver_frac","high_chip","high_PPM1D","high_ASXL1","high_NOTCH3")], na.rm=T)==0 & rowSums(blood_sample_selection[,c("twin_picked","male_and_dz_balance")],na.rm=T)==1] # Selecting samples that were chosen for blood sequencing not on the basis of their buccal sequencing results 
aux = metadata$pd[which(metadata$chemo==F & metadata$AGE>=65 & metadata$AGE<=85 & !is.na(metadata$burden_subs_passengers_blood))] # Samples of the right ages and with blood sequencing
unbiased_bloods2 = bb$sample[substr(bb$sample,1,7) %in% substr(intersect(unbiased_bloods,aux),1,7)] # Using sampleIDs
driverdens_blood = driverplot(genes2plot=drivers_blood, muts=bloodmuts, dndsout=dndsout_blood, dndsout_tcga=NULL, max_genes = Inf, sortbyfreq = T, onlysignifdnds = T, vafdnds = T, driverdensity_sampleIDs = unbiased_bloods2, gene2dc = gene2dc, plotwidth = 4, plotheight = 10, logscale = T, plotfilename = "Fig1_driver_plot_blood.pdf", plotmutsperdonor = T)
```

Comparing the driver density in blood vs buccal (restricted to the same donors).

```
driverdens_buccal_unbiasedblood = driverplot(genes2plot=drivers, muts=mutations, dndsout=dndsout, dndsout_tcga=NULL, max_genes = Inf, sortbyfreq = T, onlysignifdnds = T, vafdnds = T, driverdensity_sampleIDs = unbiased_bloods, gene2dc = gene2dc, plotwidth = 10, plotheight = 10, logscale = T, plotfilename = "kk.pdf") # Recalculating
d1 = driverdens_buccal_unbiasedblood[intersect(rownames(driverdens_blood),rownames(driverdens_buccal_unbiasedblood)),]
d2 = driverdens_blood[intersect(rownames(driverdens_blood),rownames(driverdens_buccal_unbiasedblood)),]
```

```
if (runman) { dev.new(width=6, height=3) }
barplot((d1/d2)[,1], ylab="Ratio driver % buccal/blood", xlab="", log="y", col="cadetblue", border=NA, ylim=c(0.1,20), las=2)
abline(h=1)
```

```
if (runman) { dev.copy(pdf,"Ratio_driver_percentage_buccal_vs_blood.pdf",width=7,height=4); dev.off() }
```

Analysing the VAFs of blood driver clones in the matched buccal swabs.

```
# Buccal VAFs of blood clones (VAF>1% in blood)
bloodclones = read.table(bloodclones_inbuccal_file, header=1, sep="\t", stringsAsFactors = F)
bloodclones$AGEdiff = setNames(metadata$AGE-metadata$AGE_blood, metadata$pd)[bloodclones$sampleID]

selected_clones = which(bloodclones$blood_unbiased_vaf>=0.01 & bloodclones$sampleID %in% unbiased_bloods & bloodclones$AGEdiff<=3 & !is.na(bloodclones$gene))
est_cont = median(bloodclones$vaf_buccal[selected_clones] / bloodclones$blood_unbiased_vaf[selected_clones], na.rm=T)
message(sprintf("Samples and mutations used for analysis: %0.3g, %0.3g. Median estimated contamination: %0.3g", length(unique(bloodclones$sampleID[selected_clones])), length(selected_clones), est_cont))
```

```
## Samples and mutations used for analysis: 43, 58. Median estimated contamination: 0.0763
```

```
if (runman) { dev.new(width=8.5,height=5) }
par(mfrow=c(1,2))

plot(bloodclones$blood_unbiased_vaf[selected_clones], pmax(1e-4,bloodclones$vaf_buccal[selected_clones]), xlab="VAF blood", ylab="VAF buccal", log="xy", las=2)
abline(a=0, b=1)
abline(a=log10(est_cont), b=1, col="cadetblue")

y = (bloodclones$vaf_buccal / bloodclones$blood_unbiased_vaf)
x = pmin(7,bloodclones$AGEdiff)
boxplot(y~x, notch=T, xlab="Age difference years (buccal-blood)", ylab="VAF ratio buccal/blood", las=1, ylim=c(0,1.5))
abline(h=est_cont, col="cadetblue", lty=2)
```

```
warning(sprintf("%0.0f outliers have values >1.5 and will not be plotted",sum(y>1.5)))

if (runman) { dev.copy(pdf,"Blood_clones_in_buccal_VAFs.pdf",width=8.5,height=5); dev.off() }
```

### Saturation driver discovery analyses

The high density of driver mutations identified in some genes provides a form of in vivo saturation mutagenesis. The extent of saturation achieved by the current dataset can be measured in different ways. First, a useful value is the average mutation rate per site, calculated for neutral sites. This rate is expected to vary greatly for different sites depending on their mutability. We can use the maximum-likelihood estimates of the substitution model in dNdScv as an estimate of the density of neutral mutations per site for different trinucleotide contexts. In the buccal swab dataset, the highest average neutral mutation rate per site was ~0.43-0.60 mutations/site for C>T changes in all four possible CpG contexts, and the lowest rates per site were ~0.007-0.008 mutations/site for A>C mutations at certain contexts (vector shown below). The mean rate across all 192 possible trinucleotide changes was ~0.056 mutations/site. These rates refer to the neutral mutation rate for each possible trinucleotide change. When considering SNVs, each base can change to three other bases (e.g. A can change to C, G or T), and each codon can change to nine other codons, and so the average neutral mutation density per base pair or per codon will increase accordingly. This analysis reveals that ~2 or ~9-times higher aggregate depth than currently achieved will be required to obtain an average of one mutation per neutral codon or base pair, respectively.

```
mlerates = c(setNames(dndsout$mle_submodel[,2], dndsout$mle_submodel[,1]), "TTT>TGT"=1) 
mlerates = sort((mlerates * mlerates["t"])[setdiff(names(mlerates),c("wmis","wnon","wspl","t"))], decreasing = T) # Normalised MLE rates for the 192 possible trinucleotide substitutions
print(mean(mlerates))
```

```
## [1] 0.05561794
```

```
print(mlerates)
```

```
##     CGT>CAT     ACG>ATG     GCG>GTG     CCG>CTG     CGC>CAC     TCG>TTG 
## 0.596952215 0.540979567 0.445192870 0.434637602 0.417731611 0.368789975 
##     CGA>CAA     CGG>CAG     TAT>TGT     AAT>AGT     TGC>TAC     CGA>CTA 
## 0.357919790 0.350129890 0.258277899 0.223967028 0.139474631 0.134852546 
##     TCA>TTA     TCC>TTC     GGC>GAC     CAT>CGT     CGT>CTT     TGT>TAT 
## 0.133501020 0.132665066 0.117047586 0.109424525 0.105407457 0.100920689 
##     GGG>GAG     GGT>GAT     CCA>CTA     AAC>AGC     TCT>TTT     CCT>CTT 
## 0.099767079 0.099631468 0.099081410 0.097660723 0.094866790 0.093953997 
##     CCC>CTC     TAC>TGC     GCA>GTA     ATT>ACT     AGG>AAG     TGC>TTC 
## 0.091306489 0.088734723 0.085474480 0.082257967 0.080097770 0.079863624 
##     TCT>TGT     TGA>TAA     CGC>CTC     GCC>GTC     GAT>GGT     ACC>ATC 
## 0.078490269 0.075048823 0.074642534 0.072168202 0.071921508 0.071276276 
##     GGA>GAA     AGT>AAT     GGT>GTT     GGA>GTA     AGC>AAC     TGG>TAG 
## 0.070931931 0.070501267 0.069667976 0.069500471 0.066170855 0.064981194 
##     TGT>TTT     ATA>ACA     ACA>ATA     GCT>GTT     ACT>ATT     TCG>TAG 
## 0.064735908 0.063957817 0.063811695 0.058892756 0.058173686 0.057567548 
##     AGA>AAA     CGG>CTG     GGC>GTC     GGG>GTG     CCG>CGG     CCT>CGT 
## 0.055554618 0.054788413 0.048517271 0.047804814 0.047627481 0.047026790 
##     ACG>AAG     GCG>GAG     TCC>TAC     GAT>GTT     TCG>TGG     CCG>CAG 
## 0.046547282 0.045609202 0.043809447 0.043058250 0.041740525 0.041180471 
##     ATG>ACG     GAC>GGC     TAG>TGG     TGA>TTA     CAT>CTT     TCA>TGA 
## 0.040769278 0.040178151 0.040080611 0.039961644 0.039326295 0.038536541 
##     AGT>ATT     AGA>ATA     AAG>AGG     CTT>CGT     TAT>TTT     CTG>CAG 
## 0.038117666 0.037793525 0.036910508 0.036214236 0.035938523 0.035224560 
##     GTT>GCT     ACT>AGT     ACC>AAC     GCG>GGG     CCC>CAC     TCT>TAT 
## 0.034987500 0.034868227 0.034803962 0.034001448 0.033453770 0.032489926 
##     TAA>TGA     TCC>TGC     AGA>ACA     CAC>CGC     CGT>CCT     GCC>GAC 
## 0.031750351 0.031682861 0.031324892 0.031258091 0.030855313 0.030325144 
##     GTA>GCA     CCC>CGC     AAT>ATT     CGA>CCA     TCA>TAA     GTG>GCG 
## 0.030285172 0.030108393 0.030031692 0.029826649 0.029627458 0.028880410 
##     ACG>AGG     CTG>CCG     CAG>CGG     CTA>CCA     GTC>GGC     AGC>ATC 
## 0.028758534 0.027988137 0.027710234 0.027225856 0.026485772 0.026050846 
##     ATC>ACC     ACA>AAA     GCC>GGC     AAA>AGA     TGG>TTG     GCA>GAA 
## 0.025991610 0.025739488 0.025564623 0.025305019 0.025281784 0.024615023 
##     CTA>CAA     TTT>TGT     CGG>CCG     CCT>CAT     GAG>GTG     CTC>CAC 
## 0.024433460 0.023817497 0.023253827 0.023125686 0.023079016 0.022935759 
##     CAA>CGA     GCT>GGT     GTC>GCC     GAG>GGG     CGC>CCC     CTC>CGC 
## 0.022933486 0.022876296 0.022804351 0.022667612 0.022126180 0.021926362 
##     TTT>TCT     ATC>AAC     GTG>GGG     AGT>ACT     ATT>AGT     ACT>AAT 
## 0.021457985 0.021371373 0.021364974 0.021166189 0.021112421 0.021043086 
##     AGG>ATG     GAC>GTC     AGG>ACG     TGA>TCA     GGA>GCA     GCT>GAT 
## 0.021001307 0.020898048 0.020882248 0.020815943 0.020707066 0.020661398 
##     CCA>CAA     CTC>CCC     CCA>CGA     CTG>CGG     CTT>CCT     CAG>CTG 
## 0.020443879 0.020412265 0.020290742 0.020249310 0.019986916 0.019834491 
##     ACA>AGA     GTC>GAC     TTA>TCA     GGG>GCG     TGT>TCT     ACC>AGC 
## 0.019751752 0.019648728 0.019572974 0.019481155 0.018832264 0.018540854 
##     GGT>GCT     ATG>AAG     CTT>CAT     TGC>TCC     TTC>TGC     GTG>GAG 
## 0.018468019 0.018457442 0.017921086 0.017876892 0.017379813 0.017123743 
##     GTT>GGT     TTG>TCG     GAA>GGA     TTA>TAA     TAG>TTG     GCA>GGA 
## 0.017121238 0.017007756 0.016984056 0.016745116 0.016624132 0.016377364 
##     GTA>GGA     AAG>ATG     TTG>TGG     TTC>TCC     TTA>TGA     AAC>ATC 
## 0.015946285 0.015751689 0.015742155 0.015612395 0.015078001 0.014981736 
##     ATA>AAA     TTT>TAT     AAA>ACA     CAA>CCA     TAC>TTC     TAA>TCA 
## 0.014789362 0.013866567 0.013734527 0.013698819 0.013563730 0.013466014 
##     TAA>TTA     CTA>CGA     TTG>TAG     GTA>GAA     GAA>GTA     AAG>ACG 
## 0.013418177 0.013263878 0.013232979 0.013192536 0.013167624 0.013134796 
##     AAT>ACT     GGC>GCC     ATC>AGC     GAA>GCA     TTC>TAC     CAC>CTC 
## 0.012950470 0.012845817 0.012834763 0.012804467 0.012095290 0.011926686 
##     AAA>ATA     TGG>TCG     CAA>CTA     ATG>AGG     TAT>TCT     AGC>ACC 
## 0.011514134 0.011505327 0.011476622 0.011134858 0.010906716 0.010848153 
##     CAT>CCT     CAC>CCC     CAG>CCG     ATT>AAT     AAC>ACC     GAT>GCT 
## 0.010811761 0.010739670 0.010708872 0.010616878 0.010481728 0.009858073 
##     GTT>GAT     TAG>TCG     ATA>AGA     TAC>TCC     GAG>GCG     GAC>GCC 
## 0.009726069 0.008993604 0.008694845 0.008120959 0.007985184 0.007224754
```

The description above refers to the neutral mutation density per base change, base pair, and codon. However, we note that lower aggregate depths will be needed to find the most important sites under strong positive selection (e.g. with site-dN/dS > 100), while much higher depths will be required to find individual sites under negative selection. To explore the extent to which the landscape of driver mutations is approaching saturation in our dataset, we studied the number of genes and sites under significant positive selection for progressively larger random subsets of our dataset.

```
if (file.exists("saturation_subsampling.rda")) {
  
  load("saturation_subsampling.rda")
  
} else { # If the file has not previously generated, we rerun the subsampling analysis (this will take a few hours in the buccal dataset)
  
  # Running the subsampling analyses
  selcv_subsampl = sitednds_subsampl = list() # Initialising
  subsampl_sizes = rep(seq(100,1000,by=100), each = 3)
  
  for (j in 1:nrow(sat)) {
    
    # Running dndscv
    s = sample(x=unique(mutations$sampleID), size=subsampl_sizes[j,1]) # Randomly selected samples
    m = unique(mutations[mutations$sampleID %in% s,1:5])
    d = dndscv(m, gene_list = targetgenes, max_muts_per_gene_per_sample = Inf, max_coding_muts_per_sample = Inf, constrain_wnon_wspl = T, mingenecovs = 0, dc = gene2dc/mean(gene2dc), kc = newkc, outmats = T, onesided = T, cv = dndscovs, refdb = RefCDS, maxcovs = 10, use_indel_sites = use_indel_sites)
    selcv_subsampl[[j]] = d$sel_cv
    
    # Running sitednds
    d_nondc = dndscv(m, gene_list = targetgenes, max_muts_per_gene_per_sample = Inf, max_coding_muts_per_sample = Inf, constrain_wnon_wspl = T, mingenecovs = 0, kc = newkc, outmats = T, onesided = T, cv = dndscovs, refdb = RefCDS, maxcovs = 10, use_indel_sites = use_indel_sites)
    h = sitednds(d_nondc, gene_list = targetgenes, method = "LNP")
    sitednds_subsampl[[j]] = h$recursites[h$recursites$qval<0.01,]
  
    print(j/length(subsampl_sizes))
  }
  save(selcv_subsampl, sitednds_subsampl, subsampl_sizes, file = "saturation_subsampling.rda") # Saving the output objects
}

## Calculating the saturation metrics

# Reference driver and hotspot lists
ref_drivers = drivers
ref_drivers_nsmuts = table(mutations$gene[mutations$impact!="Synonymous"])[ref_drivers] # Number of non-synonymous mutations per gene for weighting
hs = hotspots_allsites$recursites[hotspots_allsites$recursites$qval<0.01 & hotspots_allsites$recursites$impact!="Synonymous",]
ref_hotspots = paste(hs$chr,hs$pos,hs$ref,hs$mut,sep=":")

genes2plot = c("NOTCH1","TP53","RAC1","PPM1D","CHEK2","NOTCH2")
sat = data.frame(size = subsampl_sizes, genes = NA, sites = NA, genesoverlap = NA, weightedgenes = NA, sitesoverlap = NA, weightedsites = NA) # Initialising data.frame with saturation metrics
satgenes_ol = satgenes_wol = as.data.frame(array(NA, dim=c(length(subsampl_sizes),length(genes2plot)), dimnames = list(NULL,genes2plot))) # Initialising data.frame with saturation metrics

for (j in 1:nrow(sat)) {

  driv_ss = selcv_subsampl[[j]][selcv_subsampl[[j]]$qsubpos_cv<0.01, "gene_name"] # Vector of significant driver genes in this subsample
  hots_ss = paste(sitednds_subsampl[[j]]$chr,sitednds_subsampl[[j]]$pos,sitednds_subsampl[[j]]$ref,sitednds_subsampl[[j]]$mut,sep=":")  # Vector of significant sites in this subsample
  sat[j,2:7] = c(length(driv_ss), length(hots_ss), mean(ref_drivers %in% driv_ss), 
                 sum(ref_drivers_nsmuts[ref_drivers %in% driv_ss])/sum(ref_drivers_nsmuts),
                 mean(ref_hotspots %in% hots_ss), sum(hs$freq[ref_hotspots %in% hots_ss]) / sum(hs$freq))
  
  for (h in 1:length(genes2plot)) {
     satgenes_ol[j,h] = mean(ref_hotspots[hs$gene==genes2plot[h]] %in% hots_ss) # Fraction of sites discovered in the subsample
     satgenes_wol[j,h] = sum(hs$freq[(ref_hotspots %in% hots_ss) & (hs$gene==genes2plot[h])]) / sum(hs$freq[hs$gene==genes2plot[h]]) # Fraction of sites discovered in the subsample weighted by freq
  }
}

# Plotting the saturation results across all genes

if (runman) { dev.new(width=10,height=3) }
par(mfrow=c(1,4))

plot(sat$size, sat$genesoverlap, las=1, xlim=c(0,1042), ylim=c(0,1), pch=20, xlab="Number of samples", ylab="Fraction of significant genes found")
plot(sat$size, sat$weightedgenes, las=1, xlim=c(0,1042), ylim=c(0,1), pch=20, xlab="Number of samples", ylab="Fraction of mutations in significant genes")
plot(sat$size, sat$sitesoverlap, las=1, xlim=c(0,1042), ylim=c(0,1), pch=20, xlab="Number of samples", ylab="Fraction of significant sites found")
plot(sat$size, sat$weightedsites, las=1, xlim=c(0,1042), ylim=c(0,1), pch=20, xlab="Number of samples", ylab="Fraction of mutations in significant sites")
```

```
if (runman) { dev.copy(pdf,"Driver_saturation_analyses.pdf",width=10,height=3); dev.off() }
```

Plotting the saturation of driver site discovery for individual genes.

```
# Plotting the saturation results per gene

if (runman) { dev.new(width=15,height=6) }
par(mfrow=c(2,length(genes2plot)))

for (h in 1:length(genes2plot)) {
  plot(sat[,1], satgenes_ol[,h], main=genes2plot[h], las=1, xlim=c(0,1042), ylim=c(0,1), pch=20, xlab="Number of samples", ylab="Fraction of significant sites found")
}
for (h in 1:length(genes2plot)) {
  plot(sat[,1], satgenes_wol[,h], main=genes2plot[h], las=1, xlim=c(0,1042), ylim=c(0,1), pch=20, xlab="Number of samples", ylab="Fraction of mutations in significant sites")
}
```

```
if (runman) { dev.copy(pdf,"Driver_saturation_analyses_per_gene.pdf",width=15,height=6); dev.off() }
```

## 6. Simulations of clonal expansions

To better understand how driver density is expected to increase with age as a function of different growth models, we run some simple simulations with three different growth models:

1. Exponential growth: simple unconstrained growth with a fixed growth advantage over wild-type cells.
2. Quadratic growth: border growth model for 2D epithelia (Fisher et al, Nature 1958).
3. Logistic growth: constrained growth due to spatial constraints or cell-intrinsic carrying capacity.

To simplistically account for clonal competition and ensure that the total population size remains fixed, we apply a normalisation factor by dividing the sum of all mutant clones by the sum of mutant and wild-type cells.

```
### 1. Exponential growth

age = 90 # Age at sampling
r = 0.1 # Growth rate per year
mu = 1e3*4e-9 # Driver rate per year (4e-9 * 6e9 = 24 mutations per cell per year)
N0 = 1e8 # Number of cells
mutcells = N0*mu*age # Number of mutant clones throughout entire life
clonebirth = runif(n = mutcells, min = 0, max = age) # Birth time of each clone
clonesizes = exp(r*(age-clonebirth)) # Exponential function

# Calculating the increase in driver density with age
Nt = sapply(1:age, function(x) sum(exp(r*(x-clonebirth[clonebirth<x]))))
wtcells = N0 - sapply(1:age, function(x) sum(clonebirth<x)) # WT cells at each age from the simulation
dtsim = Nt / (Nt + wtcells) # Simulation

Nt = mu / r * (exp((1:age)*r) - 1)
wtcells2 = (1-((1:age)*mu)) # Analytical calculation of WT cells for each age of the simulation
dt = Nt / (Nt + wtcells2) # Analytical calculation of dt

if (runman) { dev.new(width=7,height=3) }
par(mfrow=c(1,3))
plot(1:age, dtsim, main = "Exponential model", las=1, ylab="Fraction of cells with driver", xlab="Age", col="grey50")
lines(1:age, dt, col="blue")


### 2. Quadratic growth
###    Fisher 1958 Nature. Multistage models with quadratic clonal expansions to model clonal growth in a 2D epithelium (border growth).

N0 = 1e8 # Number of cells
mu = 1e3*4e-9 # Driver rate per year (4e-9 * 6e9 = 24 mutations per cell per year)
mutcells = N0*mu*age
clonebirth = runif(n = mutcells, min = 0, max = age) # Birth time of each clone
r = 0.2

# Calculating the increase in driver density with age
getclonesizes_quad = function(clonebirth, samplingage) {
  cloneages = samplingage-clonebirth[clonebirth<samplingage]
  sizes = r*(cloneages^2)
  return(sum(sizes))
}

Nt = sapply(1:age, function(x) getclonesizes_quad(clonebirth, x))
wtcells = N0 - sapply(1:age, function(x) sum(clonebirth<x)) # WT cells at each age from the simulation
dtsim = Nt / (Nt + wtcells) # Simulation

Nt = mu * r / 3 * (1:age)^3
wtcells2 = (1-((1:age)*mu)) # Analytical calculation of WT cells for each age of the simulation (for N0=1)
dt = Nt / (Nt + wtcells2) # Analytical calculation of dt (for N0=1)

plot(1:age, dtsim, main = "Quadratic model", las=1, ylab="Fraction of cells with driver", xlab="Age", col="grey50")
lines(1:age, dt, col="blue")


### 3. Logistic growth (cell-intrinsic carrying capacity or spatial constraints)
###    We use the ecology growth function where "t" is the age of the clone. See https://en.wikipedia.org/wiki/Logistic_function#Applications

L = 500 # Maximum clone size (carrying capacity per clone)
N0 = 1e8 # Number of cells
mu = 1e3*4e-9 # Driver rate per year (4e-9 * 6e9 = 24 mutations per cell per year)
mutcells = N0*mu*age
clonebirth = runif(n = mutcells, min = 0, max = age) # Birth time of each clone
r = 0.5

# Calculating the increase in driver density with age
getclonesizes = function(L, clonebirth, samplingage) {
  cloneages = samplingage-clonebirth[clonebirth<samplingage]
  sizes = L*exp(r*cloneages) / (L - 1 + exp(r*cloneages)) # Ecological logistic growth function (Wikipedia)
  return(sum(sizes))
}

Nt = sapply(1:age, function(x) getclonesizes(L, clonebirth, x))
wtcells = N0 - sapply(1:age, function(x) sum(clonebirth<x)) # WT cells at each age from the simulation
dtsim = Nt / (Nt + wtcells)

Nt = mu * (L / r * log(L - 1 + exp(r*(1:age))) - (L/r*log(L))) # Notice that the subtraction term is needed for the definite integral (area) as it is the value of the function at t=0.
wtcells2 = (1-((1:age)*mu)) # Analytical calculation of WT cells for each age of the simulation (for N0=1)
dt = Nt / (Nt + wtcells2) # Analytical calculation of dt (for N0=1)

Nt2 = mu * (1:age) * L
dt2 = Nt2 / (Nt2 + (1-(1:age)*mu))

plot(1:age, dtsim, main = "Logistic model", las=1, ylab="Fraction of cells with driver", xlab="Age", col="grey50")
lines(1:age, dt, col="blue")
lines(1:age, dt2, col="cadetblue", lty=3)
```

```
if (runman) { dev.copy(pdf,"Clonal_growth_models.pdf",width=7,height=3); dev.off() }
```

## 7. Additional analyses during revision

To study the differential selection of key driver genes in buccal swabs vs HNSC from TCGA, we first run dndscv separately on them and plot the dN/dS ratios per gene. This reveals several interesting patterns. For example, we find that mutations in CDKN2A, NFE2L2, PTEN, HLA-B, B2M and RB1, among others, are remarkably neutral (or very weakly selected for) in normal oral epithelium despite being common drivers in HNSC. This suggests that selection on these genes is likely a later event in HNSC development, potentially in combination with other mutations. It is interesting that this list includes genes like HLA-A and B2M, which are believed to be immune escape genes, and so may be expected to be selected later in carcinogenesis, and not as first hits. Also interestingly, CDKN2A loss (which may enable growing clones to escape cellular senescence) and SMAD4 loss are also known as relatively late events in colorectal, pancreatic and oesophageal adenocarcinoma evolution, rather than as first hits (e.g. PMID: 24952744, 21245094, 24445769). Thus, the high depth of our study not only has identified many novel drivers in normal epithelium, but it provides suggestive information on whether key cancer drivers may act as first or later hits.

```
genes2plot = c(head(setdiff(drivers,c("DNMT3A","TET2")),25),head(setdiff(tcga_drivers,c(drivers,"CDKN2A.p14arf","PIK3CA")),25)) # Top 15 drivers from oral epithelium and from cancers

# Subfunction: plotting the dN/dS ratios for a group of genes
plotdnds = function(dndsout, genes = genes2plot, onlysignifdnds = T, negsign = F, ymax = Inf) {
  obsw = as.matrix(dndsout$sel_cv[,c("wmis_cv","wnon_cv","wind_cv")])
  rownames(obsw) = dndsout$sel_cv$gene_name
  colnames(obsw) = c("Missense","Nonsense+splice","Indels")
  obsw = obsw[genes2plot,]
  obsw = pmin(obsw, ymax) # Capping dN/dS values to ymax
  if (negsign) {
    obsw = -obsw
  }
  if (onlysignifdnds) {
    obsp = as.matrix(dndsout$sel_cv[,c("pmis_cv","ptrunc_cv","pindpos_cv")]); rownames(obsp) = dndsout$sel_cv$gene_name; obsp = obsp[genes2plot,]; obsw[obsp>0.01] = NA # Masking out P>0.05
  }
  pos = barplot(t(obsw), beside=T, las=2, col=c("cadetblue","darkorchid3","chocolate3"), border=NA, ylim=c(pmin(0,min(obsw,na.rm=T)),pmax(0,max(obsw,na.rm=T))), ylab="dN/dS ratios")
  if (negsign == F) { 
    abline(h=1, col="grey")
    legend("topright",y=max(apply(obsw,2,sum))*1.09,legend=colnames(obsw),fill=c("cadetblue","darkorchid3","chocolate3"),border=NA,box.col=NA)
  } else {
    abline(h=-1, col="grey")
  }
  return(pos)
}

if (runman) { dev.new() }
par(mfrow=c(2,1))

# dN/dS ratios from the buccal swabs
pos = plotdnds(dndsout, genes2plot, onlysignifdnds = F); abline(v = 25*4+0.5)
# dN/dS ratios from TCGA
plotdnds(dndsout_tcga, genes2plot, onlysignifdnds = F, negsign = T, ymax = 150); abline(v = 25*4+0.5)
```

```
##      [,1] [,2] [,3] [,4] [,5] [,6] [,7] [,8] [,9] [,10] [,11] [,12] [,13] [,14]
## [1,]  1.5  5.5  9.5 13.5 17.5 21.5 25.5 29.5 33.5  37.5  41.5  45.5  49.5  53.5
## [2,]  2.5  6.5 10.5 14.5 18.5 22.5 26.5 30.5 34.5  38.5  42.5  46.5  50.5  54.5
## [3,]  3.5  7.5 11.5 15.5 19.5 23.5 27.5 31.5 35.5  39.5  43.5  47.5  51.5  55.5
##      [,15] [,16] [,17] [,18] [,19] [,20] [,21] [,22] [,23] [,24] [,25] [,26]
## [1,]  57.5  61.5  65.5  69.5  73.5  77.5  81.5  85.5  89.5  93.5  97.5 101.5
## [2,]  58.5  62.5  66.5  70.5  74.5  78.5  82.5  86.5  90.5  94.5  98.5 102.5
## [3,]  59.5  63.5  67.5  71.5  75.5  79.5  83.5  87.5  91.5  95.5  99.5 103.5
##      [,27] [,28] [,29] [,30] [,31] [,32] [,33] [,34] [,35] [,36] [,37] [,38]
## [1,] 105.5 109.5 113.5 117.5 121.5 125.5 129.5 133.5 137.5 141.5 145.5 149.5
## [2,] 106.5 110.5 114.5 118.5 122.5 126.5 130.5 134.5 138.5 142.5 146.5 150.5
## [3,] 107.5 111.5 115.5 119.5 123.5 127.5 131.5 135.5 139.5 143.5 147.5 151.5
##      [,39] [,40] [,41] [,42]
## [1,] 153.5 157.5 161.5 165.5
## [2,] 154.5 158.5 162.5 166.5
## [3,] 155.5 159.5 163.5 167.5
```

```
if (runman) { dev.copy(pdf,"Differential_dNdS_buccal_vs_HNSC.pdf",width=8,height=8); dev.off() }
```

As a complementary test, we can use a dN/dS function designed to compare dN/dS ratios per gene between two datasets. However, we note that dN/dS ratios have a subtly different physical meaning in both cases, so caution should be used when interpreting these differences (see Supplementary Material). Thus, we present the analysis below simply to highlight that certain genes are significantly under or overrepresented in buccal swabs compared to cancer genomes, but caution should be used when interpreting these differences in terms of selection.

```
pairwisednds = function(dnds1, dnds2, genestotest) {

  pvec = rmisvec = rtruvec = rep(NA, length(genestotest)) # Initialising vectors for p-values and for the ratios of wmis and wtru between dataset 1 and 2
  w1 = dnds1$globaldnds$mle; names(w1) = dnds1$globaldnds$name
  w2 = dnds2$globaldnds$mle; names(w2) = dnds2$globaldnds$name
    
  for (g in 1:length(genestotest)) {
        
    # We can implement a simple LRT model based on the uniform dNdS model
    # This is different from the Fisher test in that it uses synonymous mutations (i.e. dN/dS ratios)
    # instead of comparing the contribution of nonsyn muts of a gene *relative* to other genes.
    # Being a uniform model it assumes no considerable changes in the mutation rate variation or coverage
    # across genes in both datasets. But takes into account signature and rate variation between two
    # datasets.
    # H0: wmis1==wmis2 & wtru1==wtru2
    # H1: wmis1!=wmis2 & wtru1!=wtru2
    # This is simply done using obs1, exp1, obs2, exp2 (y1 and y2 vectors below)
        
    y1 = as.numeric(dnds1$genemuts[dnds1$genemuts$gene==genestotest[g],])
    y2 = as.numeric(dnds2$genemuts[dnds2$genemuts$gene==genestotest[g],])
        
    # Global dN/dS ratios from all other genes (to normalise the differences for the gene being tested)        
    ind1 = dnds1$genemuts$gene!=genestotest[g]
    ind2 = dnds2$genemuts$gene!=genestotest[g]
    wmis1_global = sum(dnds1$genemuts$n_mis[ind1])/sum(dnds1$genemuts$exp_mis[ind1])
    wmis2_global = sum(dnds2$genemuts$n_mis[ind2])/sum(dnds2$genemuts$exp_mis[ind2])
    wtru1_global = sum(dnds1$genemuts$n_non[ind1]+dnds1$genemuts$n_spl[ind1])/sum(dnds1$genemuts$exp_non[ind1]+dnds1$genemuts$exp_spl[ind1])
    wtru2_global = sum(dnds2$genemuts$n_non[ind2]+dnds2$genemuts$n_spl[ind2])/sum(dnds2$genemuts$exp_non[ind2]+dnds2$genemuts$exp_spl[ind2])
        
    # MLE dN/dS ratios using the uniform model under H0 and H1
    wmis_mle0 = (y1[3]+y2[3])/(y1[7]*wmis1_global+y2[7]*wmis2_global)
    wtru_mle0 = sum(y1[4:5]+y2[4:5])/sum(y1[8:9]*wtru1_global+y2[8:9]*wtru2_global)
    wmis_mle1 = c(y1[3],y2[3])/c(y1[7]*wmis1_global,y2[7]*wmis2_global)
    wtru_mle1 = c(sum(y1[4:5]),sum(y2[4:5]))/c(sum(y1[8:9]*wtru1_global),sum(y2[8:9]*wtru2_global))
        
    # Observed and predicted counts under H0 and H1
    obs = as.numeric(c(y1[3], sum(y1[4:5]), y2[3], sum(y2[4:5])))
    exp0 = as.numeric(c(y1[7]*wmis1_global*wmis_mle0, sum(y1[8:9])*wtru1_global*wtru_mle0, y2[7]*wmis2_global*wmis_mle0, sum(y2[8:9])*wtru2_global*wtru_mle0))
    exp1 = as.numeric(c(y1[7]*wmis1_global*wmis_mle1[1], sum(y1[8:9])*wtru1_global*wtru_mle1[1], y2[7]*wmis2_global*wmis_mle1[2], sum(y2[8:9])*wtru2_global*wtru_mle1[2])) # Note that exp1 == obs (we only have this line here for confirmation purposes)
    ll0 = c(sum(dpois(x=obs[c(1,3)], lambda=exp0[c(1,3)], log=T)), sum(dpois(x=obs[c(2,4)], lambda=exp0[c(2,4)], log=T)))
    ll1 = c(sum(dpois(x=obs[c(1,3)], lambda=exp1[c(1,3)], log=T)), sum(dpois(x=obs[c(2,4)], lambda=exp1[c(2,4)], log=T)))
    
    # One-sided p-values
    pvals = (1-pchisq(2*(ll1-ll0), df=1))
    if (wmis_mle1[1]<wmis_mle1[2]) { pvals[1] = 1 } else { pvals[1] = pvals[1]/2 }
    if (wtru_mle1[1]<wtru_mle1[2]) { pvals[2] = 1 } else { pvals[2] = pvals[2]/2 }
    
    # Saving the results
    pvec[g] = 1 - pchisq(-2 * sum(log(pvals)), df = 4) # Fisher combined p-value
    rmisvec[g] = wmis_mle1[1]/wmis_mle1[2]
    rtruvec[g] = wtru_mle1[1]/wtru_mle1[2]
      
  }
  out = data.frame(genestotest,pvec,rmisvec,rtruvec)
  return(out)
}

# Running pairwisednds in our dataset
pair_dndsout = pairwisednds(dndsout, dndsout_tcga, genestotest = targetgenes)
pair_dndsout$qval = p.adjust(pair_dndsout$pvec, method = "BH")
pair_dndsout = pair_dndsout[order(pair_dndsout$qval), ]
print(pair_dndsout[pair_dndsout$qval<0.05,])
```

```
##     genestotest         pvec   rmisvec   rtruvec         qval
## 13       NOTCH2 0.000000e+00  5.189729  6.330552 0.000000e+00
## 113      NOTCH1 0.000000e+00  4.501626  7.859195 0.000000e+00
## 216       CHEK2 0.000000e+00 19.115768       Inf 0.000000e+00
## 181       PPM1D 2.629008e-13  2.843831 32.164027 1.570832e-11
## 130         ATM 1.982636e-12  2.836845       Inf 9.477001e-11
## 227       RBM10 3.216760e-08       Inf  7.155056 1.281343e-06
## 236      BCORL1 5.157502e-08  1.562733 18.679251 1.760919e-06
## 156         MGA 7.824048e-07  2.045894  8.163221 2.337434e-05
## 208       ASXL1 2.284377e-06  1.257071  5.794190 6.066291e-05
## 21      ZFP36L2 2.862025e-06 12.385429       Inf 6.840240e-05
## 230       KDM5C 8.131421e-06  3.693712  7.826309 1.766736e-04
## 161        PKD1 1.398402e-04  2.027129       Inf 2.785151e-03
## 235       STAG2 1.886340e-04  5.340723  4.383487 3.467963e-03
## 62         TET2 7.029145e-04  3.898691  2.044233 1.199975e-02
## 142       LATS2 1.413194e-03  8.103126       Inf 2.251689e-02
## 88         RAC1 1.578910e-03  2.313125       Inf 2.358497e-02
## 5        ARID1A 1.716033e-03  1.835265  2.672014 2.412541e-02
## 239         UTY 3.357584e-03       Inf       NaN 4.458126e-02
## 112        TSC1 3.969609e-03  5.678237       Inf 4.993351e-02
```

#### Analyses on the robustness of driver discovery

To provide additional support for our driver results, we first look at how many of the driver genes that we found in the buccal swabs are found under nominal significance in TCGA HNSC.

```
nomdriv = sum(dndsout_tcga$sel_cv$pglobalpos_cv[dndsout_tcga$sel_cv$gene_name %in% drivers]<0.05)
print(sprintf("Number of nominally significant drivers in TCGA HNSC: %0.0f out of %0.0f", nomdriv, length(drivers)))
```

```
## [1] "Number of nominally significant drivers in TCGA HNSC: 21 out of 49"
```

Since this buccal swab dataset is the largest of its kind, we do not expect all driver genes to have support in previous studies. However, we can use internal cross-validation to assess the robustness of our driver discovery approach. To do so, we ran dNdScv on a random 50% of the samples in the study and checked how many significant genes are found and what fraction of them are found to be nominally significant in the other half of the dataset.

```
numsims = 10
holdback = round(0.5 * length(unique(mutations$sampleID))) # Number of samples to be held back (half of the dataset)
crossval_vec = NULL

for (j in 1:numsims) {
  
  holdback_samples = sample(unique(mutations$sampleID), size=holdback, replace = F)
  m1 = unique(mutations[mutations$sampleID %in% holdback_samples, 1:5]) # Split 1
  m2 = unique(mutations[!(mutations$sampleID %in% holdback_samples), 1:5]) # Split 2
  
  # dNdScv on each subset
  dndsout_split1 = dndscv(m1, gene_list = targetgenes, max_muts_per_gene_per_sample = Inf, max_coding_muts_per_sample = Inf, constrain_wnon_wspl = T, mingenecovs = 0, dc = gene2dc/mean(gene2dc), kc = newkc, outmats = T, onesided = T, cv = dndscovs, refdb = RefCDS, maxcovs = 10, use_indel_sites = use_indel_sites)
  dndsout_split2 = dndscv(m2, gene_list = targetgenes, max_muts_per_gene_per_sample = Inf, max_coding_muts_per_sample = Inf, constrain_wnon_wspl = T, mingenecovs = 0, dc = gene2dc/mean(gene2dc), kc = newkc, outmats = T, onesided = T, cv = dndscovs, refdb = RefCDS, maxcovs = 10, use_indel_sites = use_indel_sites)

  # Reporting consistency metrics
  p1 = dndsout_split1$sel_cv$gene_name[dndsout_split1$sel_cv$psubpos_cv<0.05]
  q1 = dndsout_split1$sel_cv$gene_name[dndsout_split1$sel_cv$qsubpos_cv<0.01]
  p2 = dndsout_split2$sel_cv$gene_name[dndsout_split2$sel_cv$psubpos_cv<0.05]
  q2 = dndsout_split2$sel_cv$gene_name[dndsout_split2$sel_cv$qsubpos_cv<0.01]
  print(sprintf("Cross-validation 1 vs 2: %0.3g (%0.0f/%0.0f)", mean(q1 %in% p2), sum(q1 %in% p2), length(q1)))
  print(sprintf("Cross-validation 2 vs 1: %0.3g (%0.0f/%0.0f)", mean(q2 %in% p1), sum(q2 %in% p1), length(q2)))
  crossval_vec = c(crossval_vec, mean(q1 %in% p2), mean(q2 %in% p1))
}
```

```
## [1] "Cross-validation 1 vs 2: 0.907 (39/43)"
## [1] "Cross-validation 2 vs 1: 0.95 (38/40)"
## [1] "Cross-validation 1 vs 2: 0.884 (38/43)"
## [1] "Cross-validation 2 vs 1: 0.952 (40/42)"
## [1] "Cross-validation 1 vs 2: 0.905 (38/42)"
## [1] "Cross-validation 2 vs 1: 0.951 (39/41)"
## [1] "Cross-validation 1 vs 2: 0.974 (38/39)"
## [1] "Cross-validation 2 vs 1: 0.974 (38/39)"
## [1] "Cross-validation 1 vs 2: 0.974 (37/38)"
## [1] "Cross-validation 2 vs 1: 0.917 (44/48)"
## [1] "Cross-validation 1 vs 2: 0.881 (37/42)"
## [1] "Cross-validation 2 vs 1: 0.977 (42/43)"
## [1] "Cross-validation 1 vs 2: 0.93 (40/43)"
## [1] "Cross-validation 2 vs 1: 0.949 (37/39)"
## [1] "Cross-validation 1 vs 2: 0.951 (39/41)"
## [1] "Cross-validation 2 vs 1: 0.93 (40/43)"
## [1] "Cross-validation 1 vs 2: 0.974 (37/38)"
## [1] "Cross-validation 2 vs 1: 0.909 (40/44)"
## [1] "Cross-validation 1 vs 2: 0.947 (36/38)"
## [1] "Cross-validation 2 vs 1: 0.907 (39/43)"
```

```
print(sprintf("Mean cross-validation: %0.3g",mean(crossval_vec)))
```

```
## [1] "Mean cross-validation: 0.937"
```

#### Analyses on the variation of driver density across individuals for major driver genes

To explore this question further, we perform two new analyses:

1. Variation in driver density across donors: To better visualise the variation in driver density across donors, we can generate two additional plots showing the estimated mutant cell fraction per donor, both on aggregate across clones (i.e. 2\*sum(duplexVAF)), and for each clone individually (bamVAF, restricted to sites with high coverage).
2. Quantifying the extent of variation across genes: To explore whether certain driver genes show a more extreme variation across donors than others, while accounting for the higher sampling variation of less frequent driver genes, we can use a negative binomial regression.

To start with, we plot the variation in: (a) the estimated mutant cell fraction per donor (aggregated across clones using 2\*dVAF), and (b) the mutant cell fraction per clone for each gene.

```
#genes2plot = topdrivers
genes2plot = c(topdrivers[1:10],"DNMT3A","TET2")
nonsynmuts = mutations[which((mutations$gene %in% genes2plot) & (mutations$impact!="Synonymous")), ] # Non-synonymous mutations in the selected driver genes

# Filtering out mutations with significantly discrepant duplex and bam VAFs, as these VAFs appear unreliable
mcounts = as.matrix(nonsynmuts[,c("times_called","duplex_cov","bam_mut_adj","bam_cov_adj")])
pvec = apply(mcounts, 1, function(x) fisher.test(array(x, dim=c(2,2)))$p.value) # p-value for each mutation
qvec = p.adjust(pvec, method="BH") # q-value for each mutation
maux = nonsynmuts[which(qvec>0.05), ] # Table of non-synonymous mutations excluding those with discrepant duplex and bam VAFs

# Drivers per donor
drivperdonor = split(maux, f=maux$sampleID) # List to calculate the driver density per donor
ids = metadata$pd[metadata$AGE>=65 & metadata$AGE<=85] # SampleIDs for donors 65-85 years old
drivperdonor = drivperdonor[ids]
message(sprintf("Number of samples considered for this analysis: %0.0f", length(drivperdonor)))
```

```
## Number of samples considered for this analysis: 589
```

```
cellfrac_perdonor = array(NA, dim=c(length(drivperdonor),length(genes2plot)), dimnames = list(names(drivperdonor),genes2plot))
cellfrac_perclone = setNames(vector("list", length(genes2plot)), genes2plot)

for (j in 1:length(genes2plot)) {
  
  # 1. 2*sum(dVAF) per donor
  for (h in 1:length(drivperdonor)) {
    cellfrac_perdonor[h,j] = sum(2*drivperdonor[[h]]$duplex_vaf[drivperdonor[[h]]$gene==genes2plot[j]])
  }
  
  # 2. bamVAFs for sites of high bam coverage
  #cellfrac_perclone[[j]] = mutations$bam_adj_cellfraction[which((mutations$sampleID %in% ids) & mutations$gene==genes2plot[j] & mutations$bam_cov>=1000 & mutations$type=="snv")] # Using cell fractions (i.e. 2*adjVAF for genes in diploid chromosomes)
  cellfrac_perclone[[j]] = mutations$bam_vaf_adj[which((mutations$sampleID %in% ids) & mutations$gene==genes2plot[j] & mutations$bam_cov>=1000 & mutations$type=="snv")] # Using adjVAF for simplicity (we opt for this as VAFs may be easier to understand)
}

## Plotting the variation in driver density across donors and the variation in VAF across clones

if (runman) { dev.new() }
par(mfrow=c(1,2))

# a. Aggregate cell fractions per donor
#aux = apply(cellfrac_perdonor, 2, sort) # y-coordinate of each point
aux = cellfrac_perdonor * 100 # y-coordinate of each point
aux[aux==0] = 0.01 # Minimum value (axis will be broken at this value)
xpos = array(seq(-0.35,0.35,length.out=nrow(aux)), dim=dim(aux)) + array(rep(1:ncol(aux),each=nrow(aux)), dim=dim(aux)) # x-coordinate of each point
plot(xpos, aux, log="y", las=1, cex=0.3, ylab = "Aggregate % of mutant cells per donor (65-85 yo)", xaxt="n", xlab = "", ylim=c(0.01,50))
axis(1, at=seq(1,length(genes2plot)), labels=genes2plot, las=2)
abline(h=c(0.02, 0.05, 0.1, 0.2, 0.5, 1, 2, 5, 10, 20, 50), col="grey", lty=2)
ymed = apply(aux, 2, median)
segments(x0=(1:length(genes2plot))-0.35, x1=(1:length(genes2plot))+0.35, y0=ymed, col="coral3", lwd=3)

# b. Individual clones
aux = pmax(0.01, unlist(cellfrac_perclone) * 100) # VAF as percentage, with 0.05% as the minimum based on the typical VAFs
xpos = NULL; for (j in 1:length(cellfrac_perclone)) { xpos = c(xpos, runif(n=length(cellfrac_perclone[[j]]),-0.35,0.35) + j) }
#plot(xpos, aux, log="y", las=1, cex=0.3, ylab = "Estimated % mutant cells per clone", xaxt="n", xlab = "", ylim=c(0.02,50))
plot(xpos, aux, log="y", las=1, cex=0.3, ylab = "Unbiased VAF % per clone", xaxt="n", xlab = "", ylim=c(0.01,50))
axis(1, at=seq(1,length(genes2plot)), labels=genes2plot, las=2)
abline(h=c(0.02, 0.05, 0.1, 0.2, 0.5, 1, 2, 5, 10, 20, 50), col="grey", lty=2)
ymed = sapply(cellfrac_perclone, function(x) median(pmax(x,0.0002))) * 100
segments(x0=(1:length(genes2plot))-0.35, x1=(1:length(genes2plot))+0.35, y0=ymed, col="coral3", lwd=3)
```

```
if (runman) { dev.copy(pdf,"Variation_driver_density_across_donors.pdf",width=10,height=6); dev.off() }
```

To quantify the extent of variation in non-synonymous mutation density per gene across donors, we then use a simple negative binomial regression using duplex coverage per gene per donor as an offset.

```
dcpergeneperdonor = read.table(dcpergene_file, header=1, sep="\t", stringsAsFactors=F)
genetheta1 = genetheta2 = setNames(rep(NA,length(genes2plot)), genes2plot)

for (j in 1:length(genes2plot)) {
  nsperdonor = as.numeric(table(nonsynmuts$sampleID[nonsynmuts$gene == genes2plot[j]])[ids])
  nsperdonor[is.na(nsperdonor)] = 0
  dcperdonor = as.numeric(dcpergeneperdonor[genes2plot[j], ids])
  model = MASS::glm.nb(nsperdonor ~ offset(log(dcperdonor)) + 1)
  genetheta1[j] = summary(model)$theta
}

for (j in 1:length(genes2plot)) {
  aux = nonsynmuts[nonsynmuts$gene == genes2plot[j], ]
  nsperdonor = unlist(sapply(split(aux, f = aux$sampleID), function(x) sum(x$times_called))[ids])
  nsperdonor[is.na(nsperdonor)] = 0
  dcperdonor = as.numeric(dcpergeneperdonor[genes2plot[j], ids])
  model = MASS::glm.nb(nsperdonor ~ offset(log(dcperdonor)) + 1)
  genetheta2[j] = summary(model)$theta
}

print(genetheta2)
```

```
##    NOTCH1      TP53      FAT1    NOTCH2     CHEK2       ATM     ASXL1   ZFP36L2 
## 3.8415678 2.6751461 4.1110324 3.7654447 3.0763272 5.9671523 2.2114947 6.3102895 
##     PPM1D      RAC1    DNMT3A      TET2 
## 1.3243543 2.2082303 0.4707324 1.2049779
```

These estimates are the overdispersion parameters (size parameters of the Gamma distribution in the Poisson-Gamma interpretation), with lower values indicating higher inter-individual heterogeneity. This analysis confirms that clonal haematopoiesis genes (DNMT3A, TET2, PPM1D) have considerably larger inter-individual variation than buccal drivers, particularly when accounting for clone sizes (consistent with the semi-exponential growth of blood clones exacerbating the inter-individual heterogeneity). However, the analysis above also confirms that there is considerable inter-individual variation in driver density in the buccal drivers. TP53 in particular seems to have larger interindividual variation than the other non-blood drivers explored in this analysis.

#### Additional figures on negative selection

In response to a reviewer’s question, the code below shows the dN/dS ratios estimated for each gene for missense and truncating mutations, including confidence intervals (obtained by profile likelihood).

```
ymin = 0.01
ci95 = geneci(dndsout) # Calculating confidence intervals for all genes
```

```
## Calculating CI95 across all genes...
```

```
ci95[,-1] = apply(ci95[,-1], 2, function(x) pmax(x,ymin))
ci95mis = ci95[,c("gene","mis_mle","mis_low","mis_high")]
ci95mis = ci95mis[order(ci95mis[,2]), ]
ci95tru = ci95[,c("gene","tru_mle","tru_low","tru_high")]
ci95tru = ci95tru[order(ci95tru[,2]), ]

if (runman) { dev.new() }
par(mfrow=c(2,1))

# Missense
plot(1:nrow(ci95), ci95mis[,2], xaxt="n", xlab = "", ylab = "Missense dN/dS per gene", pch=16, cex=0.5, log="y", ylim = c(ymin,100), las=1)
segments(x0=1:nrow(ci95), y0=ci95mis[,3], y1=ci95mis[,4], col="grey40")
abline(h=1, col="grey70")
abline(h=c(0.1,10), col="grey70", lty=2)
axis(1, at=seq(1,nrow(ci95)), labels=ci95mis[,1], las=2, cex.axis=0.15)

# Truncating
plot(1:nrow(ci95), ci95tru[,2], xaxt="n", xlab = "", ylab = "Truncating dN/dS per gene", pch=16, cex=0.5, log="y", ylim = c(ymin,100), las=1)
segments(x0=1:nrow(ci95), y0=ci95tru[,3], y1=ci95tru[,4], col="grey40")
abline(h=1, col="grey70")
abline(h=c(0.1,10), col="grey70", lty=2)
axis(1, at=seq(1,nrow(ci95)), labels=ci95tru[,1], las=2, cex.axis=0.15)
```

```
if (runman) { dev.copy(pdf,"dNdSratios_per_gene.pdf",width=10,height=6); dev.off() }
```

#### Power simulations

The statistical power to detect associations between risk factors (exposures, lifestyle or germline SNPs) and changes in the mutational and clonal landscape depends on several factors, including: (1) the cohort size, (2) the frequency of the exposure or risk allele, (3) the effect size (the strength of the effect of the exposure on mutation rates or selection), and (4) the shape of the distribution of interest. To estimate our power to detect associations with different outcome variables in our dataset and for a range of effect sizes and exposure or SNP frequencies, the code below uses bootstrapping. This reveals that our dataset is better powered to detect associations with mutation burden (i.e. mutagenic effects) than with selection at the level of individual genes (i.e. selectogenic effects). The code below can use GLM or LMER tests. The results are similar (slightly more conservative with LMER), but are considerably faster with GLM.

```
lmer_flag = 0 # 0 = glm. 1 = lmer (much slower).

gwaspower = function(outcome, m, effectfold = 2, carrier = 0.2, nsim = 100, alpha = 0.05, N = NULL) {
  
  if (is.null(N)) { # If N (sample size) is not provided, we assume N = nrow(m)
    N = nrow(m)
  }
  
  pvals = rep(NA, nsim)
  m$out = m[, outcome]
  
  for (j2 in 1:nsim) {
    
    msim = m[sample(1:nrow(m), size = N, replace=T), ]
    msim$group = 0
    carrier_inds = sample(1:nrow(msim), size = round(nrow(msim)*carrier))
    msim$group[carrier_inds] = 1
    msim$out[carrier_inds] = msim$out[carrier_inds] * effectfold
    
    if (length(carrier_inds) > 0) {
      if (lmer_flag == 1) {
        # LMER
        lmermodel0 = lme4::lmer("out ~ AGE + SEX + pack_years + drink_years + missingteeth + (1|twin)", data = msim, REML=F)
        lmermodel1 = lme4::lmer("out ~ AGE + SEX + pack_years + drink_years + missingteeth + group + (1|twin)", data = msim, REML=F)
        pvals[j2] = pchisq(-2 * (as.numeric(logLik(lmermodel0))-as.numeric(logLik((lmermodel1)))), df = 1, lower.tail = FALSE) # LRT
      } else {
        # GLM
        pvals[j2] = coefficients(summary(glm("out ~ AGE + SEX + pack_years + drink_years + missingteeth + group", data = msim)))["group",4]
      }
    }
  }
  return(mean(pvals<alpha)) # Power
}

# Grid power calculation for SNV burden

plotgrid = function(outcome, metadata, pcvec, efvec, plotname = NULL, nsim = 100, alpha = 0.05, N = NULL) {
  
  if (is.null(plotname)) { plotname = outcome }
  
  powergrid = array(NA, dim=c(length(pcvec),length(efvec)), dimnames = list(pcvec, efvec))
  for (j in 1:length(pcvec)) {
    for (h in 1:length(efvec)) {
      powergrid[j,h] = gwaspower(outcome = outcome, m = metadata, effectfold = efvec[h], carrier = pcvec[j], nsim = nsim, alpha = alpha, N = N)
    }
    print(j/length(pcvec))
  }
  
  # Contour plot
  filled.contour(x = log10(pcvec), y = efvec, z = powergrid, xlab = "Carrier frequency (log10)", ylab = "Fold increase in carriers", las = 1, main = plotname,
    plot.axes = {
    axis(1)
    axis(2)
    contour(x = log10(pcvec), y = efvec, z = powergrid, add = TRUE, lwd = 2, levels = 0.8)
  })
  return(powergrid)
}

# 1. Preparing the metadata table for power analysis

outcomes = c("burden_subs_passengers","NOTCH1","TP53","PPM1D") # List of outcome variables to explore in this power analysis
predictors = c("AGE","SEX","pack_years","drink_years","missingteeth")
min_duplexcov = 200
min_age = 0
other_excluded_samples_from_regressions = ""
inds = which(metadata$duplex_cov>=min_duplexcov & metadata$AGE>=min_age & !(metadata$donor %in% other_excluded_samples_from_regressions) 
             & metadata$hpv==0 & metadata$chemo==0 & rowSums(is.na(metadata[,c(outcomes,predictors)]))==0) # Samples to be considered in the model
metasim = metadata[inds, c(predictors,outcomes,"twin")]

# LMER/GLM on SNV burden

if (runman) { dev.new() }
pcvec = 10^seq(-2.1,log10(0.5),length.out=15)
efvec = seq(1,2,by=0.05)
powergrid_burden = plotgrid(outcome="burden_subs_passengers", metadata=metasim, pcvec=pcvec, efvec=efvec, plotname = NULL, nsim = 250, alpha = 0.05)
```

```
## [1] 0.06666667
## [1] 0.1333333
## [1] 0.2
## [1] 0.2666667
## [1] 0.3333333
## [1] 0.4
## [1] 0.4666667
## [1] 0.5333333
## [1] 0.6
## [1] 0.6666667
## [1] 0.7333333
## [1] 0.8
## [1] 0.8666667
## [1] 0.9333333
## [1] 1
```

```
if (runman) { dev.copy(pdf,"Power_simulations_burden_p05.pdf",width=5,height=4.5); dev.off() }
```

Power simulations on SNV burden using p<5e-8 as a genome-wide significance cutoff for a GWAS SNP.

```
# LMER/GLM on burden
if (runman) { dev.new() }
powergrid_burden = plotgrid(outcome="burden_subs_passengers", metadata=metasim, pcvec=pcvec, efvec=efvec, plotname = NULL, nsim = 250, alpha = 5e-8)
```

```
## [1] 0.06666667
## [1] 0.1333333
## [1] 0.2
## [1] 0.2666667
## [1] 0.3333333
## [1] 0.4
## [1] 0.4666667
## [1] 0.5333333
## [1] 0.6
## [1] 0.6666667
## [1] 0.7333333
## [1] 0.8
## [1] 0.8666667
## [1] 0.9333333
## [1] 1
```

```
if (runman) { dev.copy(pdf,"Power_simulations_burden_p5e8.pdf",width=5,height=4.5); dev.off() }
```

Power simulations on NOTCH1 driver density with p<0.05 cutoff.

```
# LMER/GLM on NOTCH1
if (runman) { dev.new() }
powergrid_burden = plotgrid(outcome="NOTCH1", metadata=metasim, pcvec=pcvec, efvec=efvec, plotname = NULL, nsim = 250, alpha = 0.05)
```

```
## [1] 0.06666667
## [1] 0.1333333
## [1] 0.2
## [1] 0.2666667
## [1] 0.3333333
## [1] 0.4
## [1] 0.4666667
## [1] 0.5333333
## [1] 0.6
## [1] 0.6666667
## [1] 0.7333333
## [1] 0.8
## [1] 0.8666667
## [1] 0.9333333
## [1] 1
```

```
if (runman) { dev.copy(pdf,"Power_simulations_NOTCH1.pdf",width=5,height=4.5); dev.off() }
```

Power simulations on TP53 driver density with p<0.05 cutoff.

```
# LMER/GLM on TP53
if (runman) { dev.new() }
powergrid_burden = plotgrid(outcome="TP53", metadata=metasim, pcvec=pcvec, efvec=efvec, plotname = NULL, nsim = 250, alpha = 0.05)
```

```
## [1] 0.06666667
## [1] 0.1333333
## [1] 0.2
## [1] 0.2666667
## [1] 0.3333333
## [1] 0.4
## [1] 0.4666667
## [1] 0.5333333
## [1] 0.6
## [1] 0.6666667
## [1] 0.7333333
## [1] 0.8
## [1] 0.8666667
## [1] 0.9333333
## [1] 1
```

```
if (runman) { dev.copy(pdf,"Power_simulations_TP53.pdf",width=5,height=4.5); dev.off() }
```
